# Ecological and socioeconomic factors associated with the human burden of environmentally mediated pathogens: a global analysis

**DOI:** 10.1016/S2542-5196(22)00248-0

**Published:** 2022-11-10

**Authors:** Susanne H Sokolow, Nicole Nova, Isabel J Jones, Chelsea L Wood, Kevin D Lafferty, Andres Garchitorena, Skylar R Hopkins, Andrea J Lund, Andrew J MacDonald, Christopher LeBoa, Alison J Peel, Erin A Mordecai, Meghan E Howard, Julia C Buck, David Lopez-Carr, Michele Barry, Matthew H Bonds, Giulio A De Leo

**Affiliations:** a Woods Institute for the Environment, Stanford University, Stanford, CA, USA; b Department of Biology, Stanford University, Stanford, CA, USA; c Emmett Interdisciplinary Program in Environment and Resources (E-IPER), Stanford University, Stanford, CA, USA; d Center for Innovation in Global Health, Stanford University, Stanford, CA, USA; e Marine Science Institute, University of California Santa Barbara, Santa Barbara, CA, USA; f Earth Research Institute, University of California Santa Barbara, Santa Barbara, CA, USA; g Department of Geography, University of California Santa Barbara, Santa Barbara, CA, USA; h High Meadows Environmental Institute, Princeton University, Princeton, NJ, USA; i Hopkins Marine Station, Stanford University, Pacific Grove, CA, USA; j School of Aquatic and Fishery Sciences, University of Washington, Seattle, WA, USA; k US Geological Survey, Western Ecological Research Center, c/o Marine Science Institute, University of California Santa Barbara, Santa Barbara, CA, USA; l MIVEGEC, Université Montpellier, Centre National de la Recherche Scientifique, Institut de Recherche pour le Développement, Montpellier, France; m PIVOT, Division of Global Health Equity, Brigham and Women's Hospital, Boston, MA, USA; n North Carolina State University, Raleigh, NC, USA; o Centre for Planetary Health and Food Security, Griffith University, Nathan, QLD, Australia; p Department of Biology and Marine Biology, University of North Carolina Wilmington, Wilmington, NC, USA; q Department of Global Health and Social Medicine, Harvard Medical School, Boston, MA

## Abstract

**Background:**

Billions of people living in poverty are at risk of environmentally mediated infectious diseases—that is, pathogens with environmental reservoirs that affect disease persistence and control and where environmental control of pathogens can reduce human risk. The complex ecology of these diseases creates a global health problem not easily solved with medical treatment alone.

**Methods:**

We quantified the current global disease burden caused by environmentally mediated infectious diseases and used a structural equation model to explore environmental and socioeconomic factors associated with the human burden of environmentally mediated pathogens across all countries.

**Findings:**

We found that around 80% (455 of 560) of WHO-tracked pathogen species known to infect humans are environmentally mediated, causing about 40% (129 488 of 359 341 disability-adjusted life years) of contemporary infectious disease burden (global loss of 130 million years of healthy life annually). The majority of this environmentally mediated disease burden occurs in tropical countries, and the poorest countries carry the highest burdens across all latitudes. We found weak associations between disease burden and biodiversity or agricultural land use at the global scale. In contrast, the proportion of people with rural poor livelihoods in a country was a strong proximate indicator of environmentally mediated infectious disease burden. Political stability and wealth were associated with improved sanitation, better health care, and lower proportions of rural poverty, indirectly resulting in lower burdens of environmentally mediated infections. Rarely, environmentally mediated pathogens can evolve into global pandemics (eg, HIV, COVID-19) affecting even the wealthiest communities.

**Interpretation:**

The high and uneven burden of environmentally mediated infections highlights the need for innovative social and ecological interventions to complement biomedical advances in the pursuit of global health and sustainability goals.

**Funding:**

Bill & Melinda Gates Foundation, National Institutes of Health, National Science Foundation, Alfred P. Sloan Foundation, National Institute for Mathematical and Biological Synthesis, Stanford University, and the US Defense Advanced Research Projects Agency.

## Introduction

**Figure 1:**
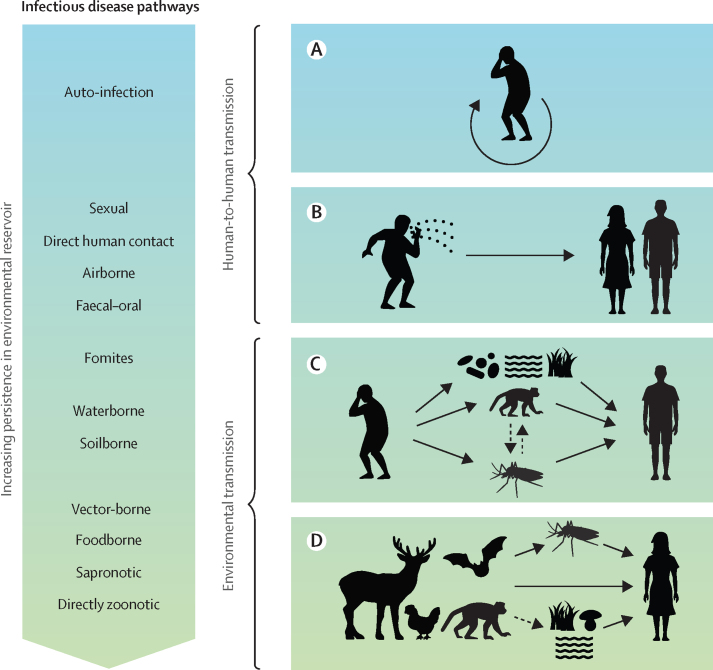
Common transmission pathways that fall along a gradient of direct and environmental transmission Direct-contact transmission strategies include (A) auto-infection, as occurs with many hospital or iatrogenic infections, and (B) human-to-human horizontal transmission by direct contact, whereas environmentally mediated transmission encompasses (C) transmission cycles whereby humans indirectly infect other humans via environmental pathways, such as food, vectors, alternative hosts, fomites, and abiotic reservoirs (soil, water) and (D) one-way spillover from environmental sources to people (with humans as dead-end hosts in the cycle). Artwork credit: N Nova.

Contact with pathogens in the environment, through water, food, waste, animals, or insect vectors, causes a major burden of human disease that is often under-recognised. Some environmentally mediated infectious diseases, such as malaria and diarrhoeal disease, cause substantial morbidity and mortality globally. Others are rare but severe or deadly, including Valley fever (*Coccidiodes* spp), caused by a soil fungus carried on dust in the wind;^1^ the free-living amoebae *Naegleria fowleri,* which can cause primary amoebic meningoencephalitis, contracted through swimming in lakes;^2^ and Nipah virus, contracted by eating fruit or drinking tree sap contaminated with infected bat urine.^3^ These examples illustrate diverse environmental transmission pathways and reservoirs (eg, fomites, soil, water, or surfaces contaminated with infective stages), vectors (eg, mosquitoes), food (eg, by contamination or trophic transmission), or non-human hosts (eg, rabies or Nipah virus from bats; figure 1). Some environmentally mediated infectious diseases have evolved human-to-human spread, as demonstrated by the recent adaptation of COVID-19 (and its pathogen SARS-CoV-2)^4^ from animal reservoirs to spread among people. Many people are now asking where the next pandemic might come from.

When the ongoing source of a human infection is environmental, biomedical and pharmaceutical treatments have limited ability to prevent new infections. Instead, reducing spread requires reducing reservoirs or exposure to environmental pathways, which falls more to ecology and sociology than medicine. To address this need, we assessed how global burdens of all human pathogens varied by transmission pathway. By contrasting patterns for directly transmitted and environmentally mediated diseases, we can gain new insights into how to reduce global disease burdens with socioecological approaches.^5^, ^6^

**Research in context**

**Evidence before this study**

We performed two extensive literature searches on the links between human infectious disease and the environment and socioeconomic context (Hopkins et al *Front Public Health* 2022; Hopkins et al *Lancet Planet Health* 2022). Our combined search indicated that most previous studies have focused on regional or country-level associations, which vary from country to country. However, WHO curates a dataset that tracks the global burden of disease attributable to environmental and occupational risks (termed the Global Health Estimates), finding 24% of global deaths are due to modifiable environmental factors. The existing research does not, however, examine the influence of most aspects of the natural environment and how they compare to that of various socioeconomic factors. Further, existing research only investigates a few selected infectious diseases, which may obscure general global patterns across all known diseases that are environmentally mediated.

**Added value of this study**

This work expands our understanding of health–environment linkages for human infectious diseases by building on the concept of environmentally mediated infections—those caused by pathogens transmitted to people via diverse environmental transmission pathways. We categorised all pathogens tracked by WHO and a random subset of all known human pathogens and found a very high fraction that are environmentally mediated. We further examined environmental and socioeconomic variables associated with higher environmentally mediated disease burdens, finding strong associations with rural poor livelihoods, and only weak associations with climatic variables, agricultural land use, or biodiversity at the global scale.

**Implications of all the available evidence**

Environmentally mediated infections represent a substantial fraction of human infectious disease burdens and have an inequitable distribution globally. Stronger focus on socioenvironmental interventions and sustainable development in parallel with patient care can help address the large and uneven global burden, contributing to better human and planetary health.

Here we define directly transmitted diseases (eg, HIV, measles, COVID-19, human influenza, human tuberculosis) as those spread primarily via person-to-person contacts, via short distance airborne or droplet spread, or through sexual transmission, vertical transmission, or autoinfection. In contrast, here we focus on environmentally mediated infectious agents that pass primarily through the environment to infect people (figure 1).

Many pathogens fall along a continuum from brief to indefinite environmental persistence (figure 1).^7^ When environmental reservoirs are not present, or are very short-lived, we categorised a pathogen as directly transmitted. Sometimes, but not always, even directly transmitted pathogens can persist on surfaces long enough to warrant a focus on environmental stages (eg, methicillin-resistant *Staphylococcus aureus* transmitted in a hospital or social distancing practices to reduce transmission of SARS-CoV-2). As defined in our study, environmentally mediated pathogens can persist in their environmental reservoirs for moderate to long periods, either free living, or as spores or cysts, or infecting a non-human biotic reservoir.

Directly transmitted pathogens can evolve from predecessors that are environmentally mediated. For example, the initial transmission (spillover) of HIV and SARS-CoV-2 from a non-human vertebrate to humans would be considered environmentally mediated by our definition (figure 1D). However, because current strains are highly effective at passing from human to human directly, we consider these pathogens directly transmitted and acknowledge that the environmental pathway to humans has now become minor, or negligible. Although environmental interventions are warranted to prevent future emergence of novel human infectious diseases, they bear less impact on current directly transmitted infections (figure 1B).

Where environmental reservoirs exist, classical biomedical disease control interventions such as drugs can treat sick patients, but in most cases, this does not prevent reinfection from environmental sources. For instance, the parasitic worm that causes river blindness (onchocerciasis) is transmitted to humans by black fly vectors. Treatment with ivermectin can eliminate the parasitic larvae from infected people, but treatment must be repeated every 6–12 months due to frequent reinfection from new black fly bites in the environment.^8^, ^9^ Complementing ivermectin campaigns with spraying for black fly vectors was key in the success of onchocerciasis control programmes across Africa in the 20th century.^10^, ^11^ As for many neglected diseases, onchocerciasis has no currently approved vaccine.^12^ Even though new technologies are slowly improving the vaccine outlook for many of these diseases,^13^, ^14^ environmentally mediated pathogens have proven difficult to control through biomedical approaches alone.

In cases such as onchocerciasis, humans play a role in maintaining transmission, but for some other environmentally mediated pathogens (eg, *Borrelia* spp causing Lyme disease), animals (eg, deer, mice, and squirrels) are the primary reservoirs. Infected people are dead-end hosts, and therefore are not involved in onward transmission. We, thus, further divided environmentally mediated pathogens by characterising whether humans are competent hosts for transmission. In other words, we account for pathogens that pass from infected people to other people through environmental pathways (eg, many human diarrhoeal pathogens and schistosomes; figure 1C), versus those that pass via unidirectional spillover from wildlife or domestic animals residing in the environment to people acting as sinks or dead-end hosts (eg, rabies virus, *Toxoplasma*; figure 1D). This distinction is important because, when a disease primarily spills over from the environment, human treatment reduces morbidity but does not impact transmission risk to other people.

Sapronoses (eg, *Clostridium tetani* bacteria that cause tetanus, and *Coccidioides* fungi that cause coccidioidomycosis) are a subset of the unidirectional spillover agents that can persist and reproduce in the environment without any host, typically obtaining their nutrition by consuming detritus or other organic matter.^15^ Although sapronoses are only rarely and opportunistically parasitic, they nevertheless remain common among the described human pathogens we studied.^15^

Environmentally mediated infections by our definition are sometimes, but not always, zoonoses. Zoonoses are defined as infectious diseases that are naturally transmissible between human and non-human vertebrate hosts. Yet, zoonotic pathogens often infect both humans and other vertebrate animals through the same environmentally mediated pathways (eg, foodborne, waterborne, vector-borne, fomites; figure 1).

Here, we were interested in the distribution, environmental drivers, and control options for the diverse array of environmentally mediated human infections. Past work has suggested that environmental, socioeconomic, and demographic factors can explain variation in disease burden from country to country, but the specific drivers vary.^16^ To explore disease drivers in the context of direct versus environmentally mediated disease transmission, we assembled a dataset characterising the main and alternative transmission pathways of the most burdensome, WHO-tracked^17^, ^18^ human pathogens and a random subset of all described human pathogens.^19^ In the following sections we: (1) quantify the distribution and burden of environmentally mediated human infections, (2) use a structural equation model to examine the direct and indirect drivers of environmentally mediated infectious disease burdens, and (3) outline recent challenges in control of environmentally mediated infectious diseases and prevention of emergence of new human-to-human strains. Although many environmentally mediated pathogen species pose a major challenge to global health, they are rarely studied as a single category. In contrast to the biomedical focus for controlling directly transmitted diseases, a human–environment systems approach might be key for controlling environmentally mediated diseases.

## Methods

### Categorising environmentally mediated and directly transmitted pathogens

We focused on pathogens whose dominant infectious pathways are environmental: that is, environmental exposures would need to be interrupted to reduce disease prevalence or persistence in humans. In classifying pathogens, we acknowledge that many pathogens have multiple pathways by which they infect their hosts. For example, cholera can pass directly, through faecal–oral pathways, or through consumption of contaminated water or food. Ebola can spill over from environmental reservoirs, which can prompt human-to-human epidemics.^20^ By focusing on the dominant pathway, we thus defined environmentally mediated diseases narrowly and avoided classifying all diseases with any environmental component under our definition. As a result, we were conservative in our definition. We categorised Ebola virus as primarily having direct human-to-human transmission; similarly, adenoviruses A–F and some rotaviruses were designated as directly transmitted because of their high faecal–oral contagion, which often limits the amount of time they spend in the environment, even though they can sometimes be found in water or wastewater (see appendix pp 3–13 for the list of all pathogens assessed and their designations as environmentally mediated or not).

### Data collection

We started with a full list of 560 named pathogens (from 197 genera) associated with WHO's tracked pathogens within category I.A: “Communicable, maternal, perinatal and nutritional conditions: Infectious and parasitic diseases” of WHO's Global Health Estimates (appendix p 2).^17^, ^18^ Next, to account for potential biases that result from the selection of pathogens that WHO tracks, we also examined the transmission strategies of a random subsample of 250 pathogens (using a random number generator to select pathogens from the full list of 1415 described human pathogen species) compiled by Taylor and colleagues in 2001,^19^ which is dominated by rare, opportunistic pathogens (appendix pp 3–13). By chance, 87 pathogens (from 57 genera) ended up in both the WHO and Taylor subsets (<15% overlap) with the remaining pathogens unique to each list. Across both datasets, we assessed 723 unique human pathogen species (from 292 genera) in total (appendix pp 3–13).

To quantify the global burden caused by environmentally mediated infections, we examined data for all countries around the world using disability-adjusted life years (DALYs), a standard metric for measuring the impact of disease on human wellbeing. DALYs are calculated as the sum of years of life lost due to mortality and years of healthy life lost due to disability.^21^ Burden data were available for a subset of 153 WHO pathogens, categorised into 51 tracked disease categories (appendix p 40).

### Data analysis

We hypothesised that their environmental affiliations predisposed environmentally mediated human infectious diseases to be more sensitive to ecological and climatic shifts along latitudinal gradients, such as shifts in biodiversity, land conversion to agriculture, or temperature, compared with direct-contact transmitted human diseases, which we hypothesised would be driven by human-centric predictors such as health-care access and political stability. We also hypothesised that rural livelihoods would put people into closer contact with their environments, potentially predisposing them to higher burdens of environmentally mediated diseases. To examine the social, economic, environmental, and ecological indicators most associated with environmentally mediated, compared with direct-contact transmitted, disease burdens across the globe, we followed the approach of Wood and colleagues^16^ in using partial least-squares structural equation modelling (PLS-SEM). PLS-SEM path modelling is a statistical method for partitioning complex covariance relationships that is particularly suited (more suited than linear regression modelling) to disentangling complex webs of predictors and outcomes that are all highly correlated (see appendix p 1). To reduce the possibility of overfitting, we constrained the initial model to a priori hypothesised drivers (summarised in appendix p 41) in the software package SmartPLS (SmartPLS, Boenningstedt, Germany). In brief, we hypothesised that the following environmental and social variables would be involved in the causal web leading to environmentally mediated or directly transmitted infectious disease burdens (appendix pp 40–41): political stability (World Bank indicator PV.EST: the perceptions of the likelihood of political instability or politically motivated violence, including terrorism); land area in agriculture (measured as a composite of World Bank indicators: permanent cropland [percentage of land area] and agricultural land [percentage of land area]); wealth (gross national income per capita, purchasing power parity); rural poor livelihood (percentage of people using at least basic sanitation services, fertility rate [total births per woman], and rurality [percentage of population living rurally, which is by World Bank definition the percentage of the population not living in urban areas]); biodiversity (measured as a composite variable made up by: area-adjusted mammal, bird, and amphibian species richness, plus percentage of forested area and percentage of protected area in each country);^16^ access to and investment in health care (a composite of current health-care expenditure per capita, measles immunisation [percentage of children ages 12–23 months], and WHO composite coverage index [%]); average lifespan; malnutrition (prevalence of undernourishment); food production (UN Food and Agriculture Organization food balance sheet); altitude (percentage of total population living in areas where elevation is below 5 metres); and climate (a composite of percentage of the 1995 population in Koeppe-Geiger temperate and tropical zones, mean precipitation, and mean temperature for 1961–99). We first assembled the a priori model (appendix p 41) based on our hypotheses about all plausible latent variables we expected might be directly or indirectly correlated with our outcomes of interest: for example, we hypothesised that land area under agricultural use might correlate with biodiversity, and biodiversity might then correlate with environmentally mediated disease burdens. After assembling all the variables in logical networks by linking them through proposed paths, we used PLS-SEM to estimate which paths were supported by the data and to what degree (path coefficients weights). Then, to further reduce overfitting, we used bootstrapped p values to retain only the significant or marginally significant correlations (p<0·1) in the final model (appendix p 41).

### Role of the funding source

The funders of the study had no role in study design, data collection, data analysis, data interpretation, or writing of the report.

## Results

At least 80% (455 of 560) of human pathogens that are tracked by WHO primarily used environmentally mediated transmission (table 1, figure 1, appendix pp 3–13). Moreover, considering a random subset of all human pathogens in the Taylor dataset corroborates this high percentage, with an estimate that 74% (186 of 250) of all human infectious agents exhibit environmentally mediated transmission (table 1).

**Table 1:** Frequency of environmentally mediated human pathogens (with transmission details)

	**Random subset of all human pathogens (n=250)**	**WHO-tracked human pathogens (n=560)**	**Example pathogen**
**Environmentally mediated human infectious diseases**			
Sapronotic	67 (27%)	160 (29%)	*Histoplasma*
Foodborne	31 (12%)	120 (21%)	*Salmonella*
Vector-borne	33 (13%)	85 (15%)	*Plasmodium*
Environmental contact (water, soil, nosocomial, etc)	23 (9%)	52 (9%)	*Schistosoma*
Zoonotic (direct contact: wildlife)	5 (2%)	19 (3%)	Rabies virus, SARS-CoV-2 (initial spillover)
Zoonotic (direct contact: domestic species)	6 (2%)	9 (1%)	*Pasteurella*
Transmission unclear	21 (9%)	10 (2%)	*Rhodococcus*
Subtotal: environmentally mediated	186 (74%)	455 (80%)	..
**Non-environmentally mediated human infectious diseases**			
Direct-contact transmitted (direct, sexual, etc)	18 (7%)	80 (14%)	HIV, SARS-CoV-2 (post-spillover)
Opportunistic (auto-infection with normal flora)	26 (10%)	14 (4%)	*Staphylococcus*
Transmission unclear	3 (1%)	1 (0·2%)	*Selenomonas* (gingivitis-causing bacterium)
Subtotal: non-environmentally transmitted	47 (19%)	95 (18%)	..
**Unknown**			
Insufficient data	17 (7%)	10 (2%)	..

Data are n (%). Species identified as environmentally mediated among a random subset (250) of the 1415 described human pathogens described by Taylor and colleagues (2001)^19^ and among the 560 human pathogens tracked by WHO for the Global Health Estimates (2015), in the category I.A: “Communicable, maternal, perinatal and nutritional conditions: Infectious and parasitic diseases.”^17^, ^18^

We summed estimated DALYs for all those human pathogens in the WHO database that were classifiable as environmentally mediated or not, which estimated 40% (129 488 of 359 341 DALYs) of the total global infectious disease burden was due to environmentally mediated infections (appendix p 40). Among these, malaria and environmentally transmitted diarrhoeal diseases (eg, shigellosis, cholera) collectively carried the highest burdens of DALYs in 2015, followed by environmentally mediated neglected tropical diseases (eg, schistosomiasis, Chagas disease, leishmaniasis), and fungal and parasitic meningeal infections. In sum, death and disability from environmentally mediated diseases cost humans nearly 130 million years of healthy life per year, based on the 2015 data we analysed (appendix p 40).

**Figure 2:**
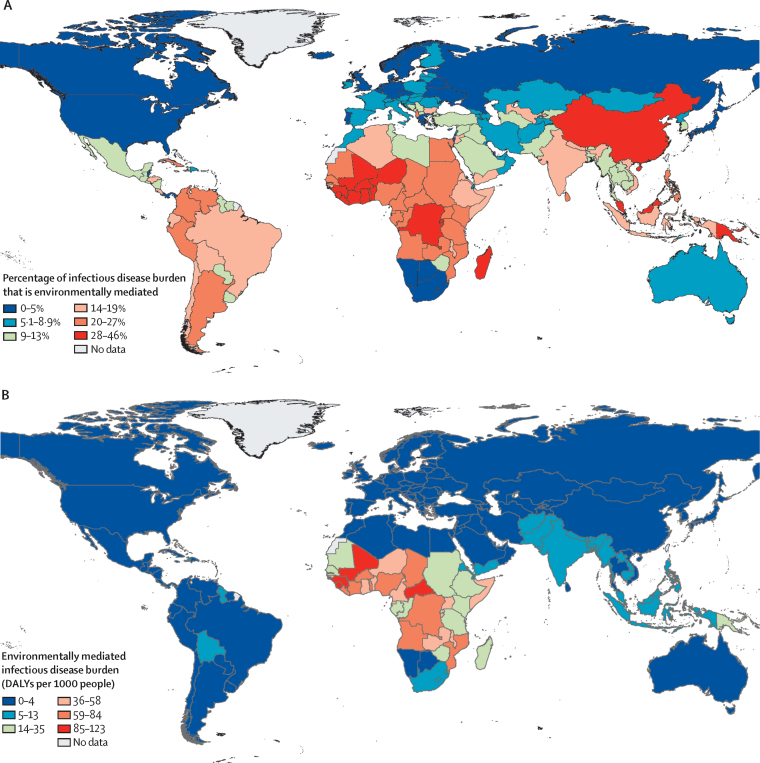
Global distribution of environmentally mediated human infectious disease burdens The maps show the uneven global distribution of environmentally mediated human infectious disease burdens; (A) as a proportion of all category I.A: “Communicable, maternal, perinatal and nutritional conditions: Infectious and parasitic diseases” in WHO's Global Health Estimates (ie, proportion of DALYs attributable to environmentally mediated infections per country out of total DALYs attributable to infectious and parasitic diseases); and (B) as total global per capita environmentally mediated infectious disease DALYs in each country. DALY=disability-adjusted life year.

**Figure 3:**
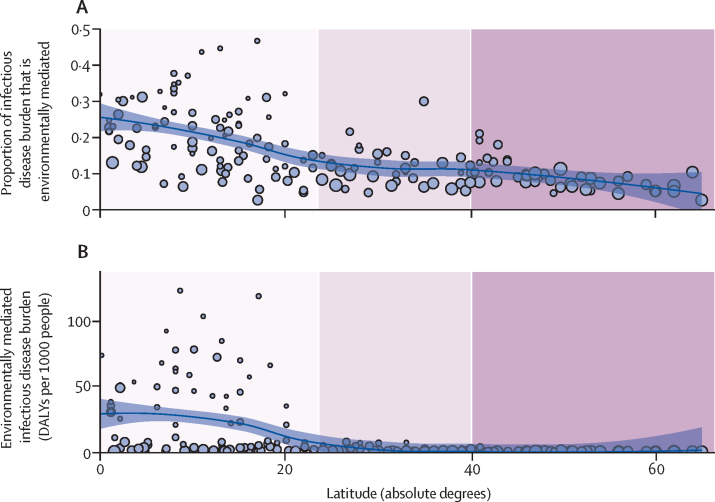
Environmentally mediated infectious disease burden by latitude (A) Latitudinal gradients in environmentally mediated infectious disease DALYs as a proportion of all category I.A: “Communicable, maternal, perinatal and nutritional conditions: Infectious and parasitic diseases” DALYs tracked by WHO's Global Health Estimates study in 2015. Countries at lower latitudes have a higher proportion of their disease burdens caused by environmental pathogens. (B) Latitudinal gradients in total environmentally mediated infectious disease DALYs per 1000 people in 2015. Each circle represents one country and the size of the circle is proportional to each country's per capita gross domestic product (sourced from World Bank 2015 World Bank Open Data). Poorer countries in all latitudinal bands (smaller dots) carry higher (A) proportions as well as (B) total burdens of environmentally mediated infectious disease. The purple bands represent three groupings of latitude (absolute degrees): tropical (0 to 23·5 degrees), subtropical (23·5 to 40 degrees), and temperate areas (over 40 degrees). DALY=disability-adjusted life year.

Environmentally mediated human infectious diseases followed a strong latitudinal gradient, even stronger than that seen for the background latitudinal gradient in all human infectious diseases: burdens declined away from the equator, such that the tropics accounted for the vast majority of the total global burden of environmentally mediated human infectious diseases, and the poorest countries carried the highest proportions of their total DALY burdens from environmentally mediated infections (Figure 2, Figure 3).

**Figure 4:**
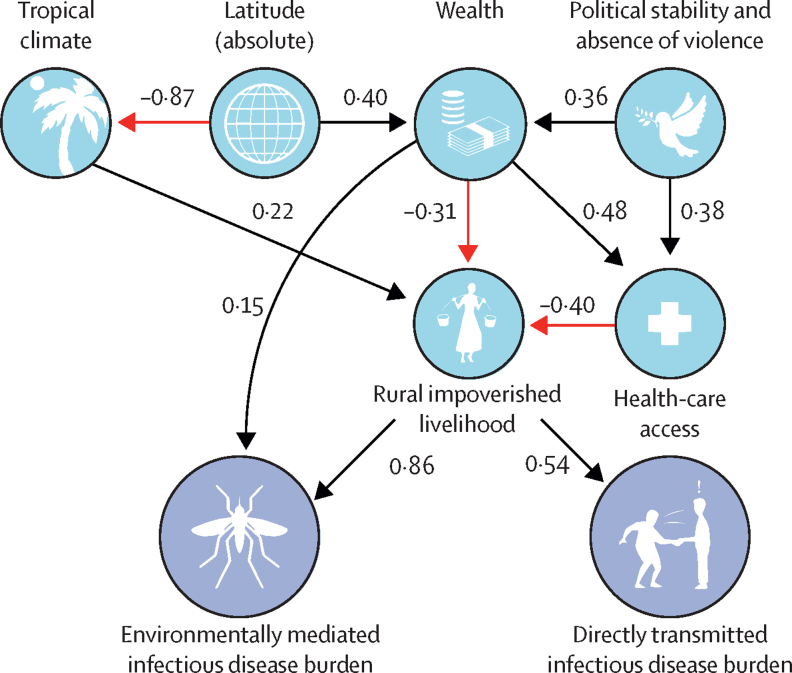
Results of partial least squares structural equation model Statistically significant paths links to total per capita burden of all classifiable directly transmitted (eg, via handshake or coughing) versus environmentally mediated infectious diseases globally are shown, with symbols representing the relevant latent variables (definitions, sample sizes, and measurement indicators for each latent variable are given in appendix pp 38–39). Red lines represent negative associations, and black lines positive associations, among the variables linked by those lines. Numbers along paths (and also path thickness) correspond to the weighted correlation coefficients which signify the strength of the association between two linked variables; total effects can be estimated by multiplying path coefficients along one or more segments, and summing across all possible paths. Total significant effects on disease burdens are summarised in appendix p 40; paths with p>0·1 were removed from the full model to produce the final model shown here (see appendix pp 42, 44). Artwork credit: N Nova.

The overall model fit of the PLS-SEM captured 41% (R^2^ adjusted=0·405) of the variation in directly transmitted disease burdens and 62% (R^2^ adjusted=0·624) of the variation in environmentally mediated disease burdens (appendix p 42). We found that, counter to our hypotheses, agricultural land use and biodiversity variables were only weakly correlated with environmentally mediated disease burdens (appendix p 41, table 2, figure 4). In contrast, human-centric variables, in particular the presence of rural poor livelihoods, were strongly associated with burden of human infectious diseases (ie, with largest total effect sizes; table 2, figure 4), with the direct effect greater for environmentally mediated disease (standardised path coefficient 0·86) versus for directly transmitted disease (standardised path coefficient 0·54). This finding was further supported by the fact that a higher proportion of infectious disease DALYs are caused by environmentally mediated diseases in the poorer countries of the global south (Figure 3, Figure 4). In general, socioeconomic variables such as wealth, rural livelihood, and health-care access had large total effect sizes, compared with smaller effects of environmental variables such as biodiversity, climate, and agricultural predictors (table 2). Strong latitudinal effects were mediated indirectly, mostly through the tropical distribution of rural poor livelihoods (as measured by the proportion of that country's population living in rural areas, lack of access to improved sanitation, and the average fertility rate; table 2, figure 4, appendix p 43).

**Table 2:** Results of the reduced (final) PLS-SEM path modelling analysis: standardised coefficients of direct paths versus indirect paths, and total effects (the sum of the previous two)

	**Directly transmitted disease burdens**			**Environmentally mediated disease burdens**		
	Direct effects (p value)	Indirect effects (p value)	Total effects (p value)	Direct effects (p value)	Indirect effects (p value)	Total effects (p value)
Biodiversity	..	..	..	0·07 (0·18)	..	0·07 (0·18)
Health-care access	..	−0·25 (<0·0001)	−0·25 (<0·0001)	..	−0·34 (<0·0001)	−0·34 (<0·0001)
Latitude	..	−0·25 (<0·0001)	−0·25 (<0·0001)	..	−0·31 (<0·0001)	−0·34 (<0·0001)
More tropical climate	..	0·14 (0·00045)	0·14 (0·00045)	..	0·23 (<0·0001)	0·23 (<0·0001)
Malnutrition	0·16 (0·079)	..	0·16 (0·079)	..	..	..
Political stability and lack of violence	..	−0·21 (<0·0001)	−0·21 (<0·001)	..	−0·23 (<0·0001)	−0·23 (<0·0001)
Rural livelihood	0·54 (<0·0001)	0·09 (0·086)	0·63 (<0·0001)	0·86 (<0·0001)		0·86 (<0·0001)
Wealth	..	−0·32 (<0·0001)	−0·32 (<0·0001)	0·15 (0·0015)	−0·43 (<0·0001)	−0·28 (<0·0001)

See figure 4, and appendix p 41, for more detail on the reduced model structure, including direct and indirect paths to disease burdens. Blank cells indicate that a coefficient was not applicable due to no possible path to disease burden, despite its inclusion in the reduced model. Note that hypothesised, but non-significant, predictors such as land area in agriculture, elevation, and total land area were not included in the reduced model (therefore not included in this results table). PLS-SEM=partial least-squares structural equation modelling.

## Discussion

The strongest country-level indicator of environmentally mediated human infectious disease burden was living in rural-poor contexts. This highlights a global health disparity and runs counter to our initial hypothesis that environmental variables largely drive the environmentally mediated infections. Socioeconomic drivers likely interact strongly with the environmental components of risk: for high burdens of environmentally mediated diseases to occur, both the social and environmental components need to be present and to align in space and time. The distribution of rural poor livelihoods was strongly associated with both environmental risk and high human burdens, supporting the importance of the often overlooked (in ecological analyses) human exposure and vulnerability dimensions to the risks of environmentally mediated pathogens.^22^ In other words, the toll of environmentally mediated pathogens is highest where humans rely on, and interact frequently with, natural ecosystems where reservoirs, vectors, and intermediate (non-human) hosts reside. Furthermore, these results support the disease-driven poverty trap hypothesis,^23^, ^24^, ^25^ which posits that poor people can become entangled in a reinforcing cycle of poverty and disease in which they are more exposed and more vulnerable to environmentally mediated infections.

In addition, political stability, wealth, and health-care effects were found to be strongly but indirectly correlated with environmentally mediated disease burdens: political stability was correlated with increased wealth, and wealth led to improved access to sanitation, clean water, health care, and other factors influencing rural, poor livelihoods (appendix pp 41, 44; table 2, figure 4). This finding suggests that direct investment in health care and development will need specific allocation to the rural poor populations that are most vulnerable (figure 4) in order to impact environmentally mediated infections.^26^

Our results support previous theoretical,^27^, ^28^, ^29^ empirical,^16^ and meta-analytic^30^ studies that have found variable effects of land-use and biodiversity on human infectious disease. Hypothesised drivers of disease burden are usually either social (population density, wealth, health-care access) or environmental (climate, biodiversity, or proxies thereof);^16^, ^23^, ^25^, ^31^ rarely are social and environmental variables assembled into a single model, as we have done here. Combining these variables into one PLS-SEM path analysis suggests that, although biodiversity and agricultural land use effects are present (and valid for some individual diseases), they are surprisingly weak predictors of overall disease burden, including environmentally mediated disease burden. Therefore, managing how environmental exposure interacts with socioeconomic conditions might lead to the most concrete health outcomes.

We deliberately focused on the total burden of environmentally mediated human infections as an outcome variable. This differs from some other analyses that have focused on burdens of non-infectious diseases attributable to pollution and the built environment,^32^ or focused on disease emergence or risk.^33^, ^34^ This likely explains why our results differ from previous studies on emerging infectious diseases, which tend to be driven strongly by biodiversity, habitat fragmentation, and human–animal contact (ie, spillover^33^). In most circumstances, emerging infectious diseases are expected to contribute little to the global disease burden, except in the most exceptional cases (such as the COVID-19 pandemic) and therefore most one-way environment-to-human spillover events are not strongly reflected in the global burden of disease data tracked over time by WHO and analysed here.

The weak associations of environmentally mediated disease burden with land use or biodiversity at the global scale might reflect a reality that drivers of each particular disease can vary across socioecological settings that are difficult to capture in country-scale analyses. For example, conservation biologists and ecologists point out links between human malaria incidence and deforestation in some areas of the tropics and not others, with the strongest effects at deforestation frontiers.^29^, ^35^, ^36^, ^37^, ^38^ Similarly, links between schistosomiasis incidence and dam construction mainly occur across the poorest regions of Africa where disease mitigation is constrained by lack of resources.^39^, ^40^, ^41^, ^42^ Future research will need to answer many basic questions about the socioecological systems that underpin environmentally mediated pathogens in order to implement effective socioecological solutions.

Although environmentally mediated infectious disease burdens were not strongly associated with biodiversity or land use in our analysis, environmentally mediated disease diversity was strongly affected by latitudinal and climatic factors, and range limits were more evident for the environmentally mediated human infectious diseases compared with the human-to-human directly transmitted infectious diseases (table 2; appendix p 43). This suggests that diseases for which humans serve as the main reservoirs are less restricted by climatic factors, and less subject to latitudinal gradients in biodiversity and climate. Although gradients do still play a role for both environmentally and directly transmitted human diseases (Figure 3, Figure 4; appendix p 43), those reliant on non-human hosts (especially invertebrates and ectotherms), or abiotic reservoirs, are more strongly limited at higher latitudes.^43^

In addition, although most environmentally mediated infectious disease burdens occur in the tropics, some high-income, temperate countries do see transmission of environmentally mediated infections (eg, coccidiodomycosis, Lyme disease, and Hendra virus, Ross River virus, and nosocomial infections) that merit attention. For example, better and more sustainable interventions are needed to curb Hendra virus spillover from bats in Australia, and Lyme disease from ticks and wildlife in North America and Europe, as medical options to control these infections are limited. Climate change might also change the distribution of some environmentally mediated diseases.

There are limitations to our study. Using aggregate data at the country level introduces the problem that data and relationships might be different depending on the spatial scale of aggregation used. This, in turn, means that inferences might differ at a finer or coarser spatial scale than that analysed.^44^ Finer (sub-country scale) data might reveal tighter associations of environmentally mediated disease burdens with climatic, biodiversity, or land-use predictors.^45^ However, we note that recent analyses have spotlighted that some environmentally mediated diseases (eg, hookworm), long thought to be eliminated in the USA, are still prevalent in the poorest communities,^46^ lending some evidence that our main results about rural subsistence livelihood as a driver of environmentally mediated disease. Nevertheless, finer resolution (sub-country) data on disease burden, such as that undertaken by the Institute for Health Metrics and Evaluation's Local Burden of Disease project, which has been completed for only a few select diseases so far,^47^ coupled with finer-scale data on the predictor variables could be used to examine social and environmental determinants across scales in the future.

Controlling environmentally mediated infections can be harder than controlling directly transmitted human diseases in some ways, and easier in others. On one hand, reinfection from environmental reservoirs can be common, and our results support the idea that exposure is often entangled with poverty and subsistence livelihoods, introducing challenging complexity. On the other hand, socioecological interventions targeting human–environment interactions, such as provision of water filters or bed nets in poor communities, that are not effective for most directly transmitted person-to-person infections might be highly impactful for several environmentally mediated infectious diseases. In other words, environmental transmission pathways are complex but allow for a wider array of socioecological levers—interventions that interrupt environmental exposure or reduce vulnerability—that could complement conventional medical approaches.^5^, ^6^ For example, although malaria vaccine trials have made news for decades, the most dramatic declines in malaria have occurred with the rapid scale-up of insecticide-treated bed nets.^48^, ^49^ Similarly, for schistosomiasis, caused by the environmentally mediated snail-borne parasite *Schistosoma*, control programmes have been most successful when they incorporate control of parasite-carrying snails in the environment.^50^, ^51^ Guinea worm is another environmentally mediated and poverty-associated parasite that has been reduced from 3·5 million cases in the 1980s to less than three dozen detected cases worldwide in 2019, without a drug or a vaccine.^52^ This remarkable success was achieved through behaviour change, simple water filters, and water supply improvements, key socioecological interventions that target the environmental pathways of transmission and the rural and vulnerable populations that are most exposed.^53^

In conclusion, environmentally mediated transmission is common among human pathogens: most human pathogens assessed are environmentally mediated and, as a group, these cause more than one-third of the global burden of human infectious diseases tracked by WHO. Our results further show a stark disparity in the high global burden of environmentally mediated pathogens, with rural poor livelihoods being one of the strongest explanatory drivers. Most environmentally mediated pathogens lack effective vaccines and treated patients are often reinfected due to their continued contact with unhealthy environments.

Challenges for controlling environmentally mediated pathogens are multifaceted and substantial, including an expanding funding gap,^54^ rising evolved resistance to insecticides and drugs,^55^, ^56^ and uneven surveillance.^54^ The high and unequal distribution of disease burden amid these challenges argues for a renewed focus on environmentally mediated human infectious diseases. The UN Sustainable Development Goals^57^, ^58^ and the recent academic emphasis on the new field of planetary health^59^ are drawing attention to the connections between human health, environmental change, and development. A renewed focus on how environmental exposures interact with socioeconomic and ecological factors to drive high and unequal burdens of environmentally mediated disease could lead to better outcomes for sustainable and equitable global health.

## Data sharing

All data synthesised during this project and used in our analyses is available in a Dryad repository (https://doi.org/10.5061/dryad.dncjsxm2x), along with relevant metadata.

## Declaration of interests

We declare no competing interests.
