# Supplementary material for: Ecological and socioeconomic factors associated with the human burden of environmentally mediated pathogens: a global analysis
Source: Lancet Planet Health. 2022 Nov 10;6(11):e870–9. doi: 10.1016/S2542-5196(22)00248-0 (PMC9669458; doi:10.1016/S2542-5196(22)00248-0)

THE LANCET
Planetary Health

Supplementary appendix

This appendix formed part of the original submission and has been peer reviewed. We post it as supplied by the authors.

Supplement to: Sokolow SH, Nova N, Jones IJ. Ecological and socioeconomic factors associated with the human burden of environmentally mediated pathogens: a global analysis. *Lancet Planet Health* 2022; **6:** e870–79.

Supplemental methods, tables and figures

The partial least squares structural equation modeling approach:
Structural Equation modeling (SEM) is a statistical approach with two key features: 1) like path analysis (which is a type of SEM) can be used to analyze a network of interacting variables, i.e. it allows for more than one dependent variable in a single analysis (and a variable can simultaneously act as a dependent and an independent variable); and 2) SEM adds the ability to create latent (or composite) variables. There are a couple of different types of latent variables, but in short, a latent variable is not directly measured, but is informed by other correlated or complimentary variables. The latent variables considered here (e.g. 'health care access' or 'land area in agriculture'), along with their correlated measurement variables are shown in Table S3 (e.g. for the latent variable 'land area in agriculture': measurement variables were World bank indicator AG.LND.CROP.ZS "Permanent cropland," as % of total land area, and World Bank indicator AG.LND.AGRI.ZS "Agricultural land," as % of total land area).

SEM models, like regression models, are based on *a priori* hypotheses about how a system works. Here, we defined a full *a priori* model (Table S4). We reduced the full model by removing paths that were non-significant by bootstrapping at an alpha of >0.05 (although we chose to retain one and only marginal effect, that of biodiversity on environmentally mediated disease burden, with a p = 0.07 due to interest in this relationship within the disease ecological field), then re-ran the SEM and reported the final model R^2 for each variable fit (Table S5) and weights and p values for each direct and indirect path between predictors and disease burdens (Table S6).

A particular type of SEM, partial least squares SEM, or PLS-SEM, was used here because it is more flexible with respect to distributional assumptions than traditional (covariance based) SEM, and therefore more appropriate for the complex and non-normal epidemiological data and predictor variables (Garson 2016). Needless to say, we have a limited sample size due to limit in the number of countries around the world. Simulation studies comparing PLS-SEM and covariance-based SEM tend to confirm that PLS serves prediction purposes well even when sample size is small (e.g. 100) (Reinartz, Haenlein et al. 2009). In addition, resampling methods, like bootstrapping used here, to determine significance do not have specific sample size requirements but the larger the sample, the more reliable the PLS estimates and the less likely that confidence intervals are fit to noise in the data instead of the underlying distribution (Garson 2016).

References for this section:

Garson, G. D. (2016). Partial Least Squares: Regression & Structural Equation Models. Asheboro, NC, Statistical Publishing Associates.
Jenkins, C. N., S. L. Pimm and L. N. Joppa (2013). "Global patterns of terrestrial vertebrate diversity and conservation." Proc Natl Acad Sci U S A **110**(28): E2602-2610.
Reinartz, W., M. Haenlein and J. Henseler (2009). "An empirical comparison of the efficacy of covariance-based and variance-based SEM." International journal of Research in Marketing **26**(4): 332-344.
Wood, C. L., A. McInturff, H. S. Young, D. Kim and K. D. Lafferty (2017). "Human infectious disease burdens decrease with urbanization but not with biodiversity." Philosophical Transactions of the Royal Society B-Biological Sciences **372**(1722).

Flow chart of data used in this manuscript:

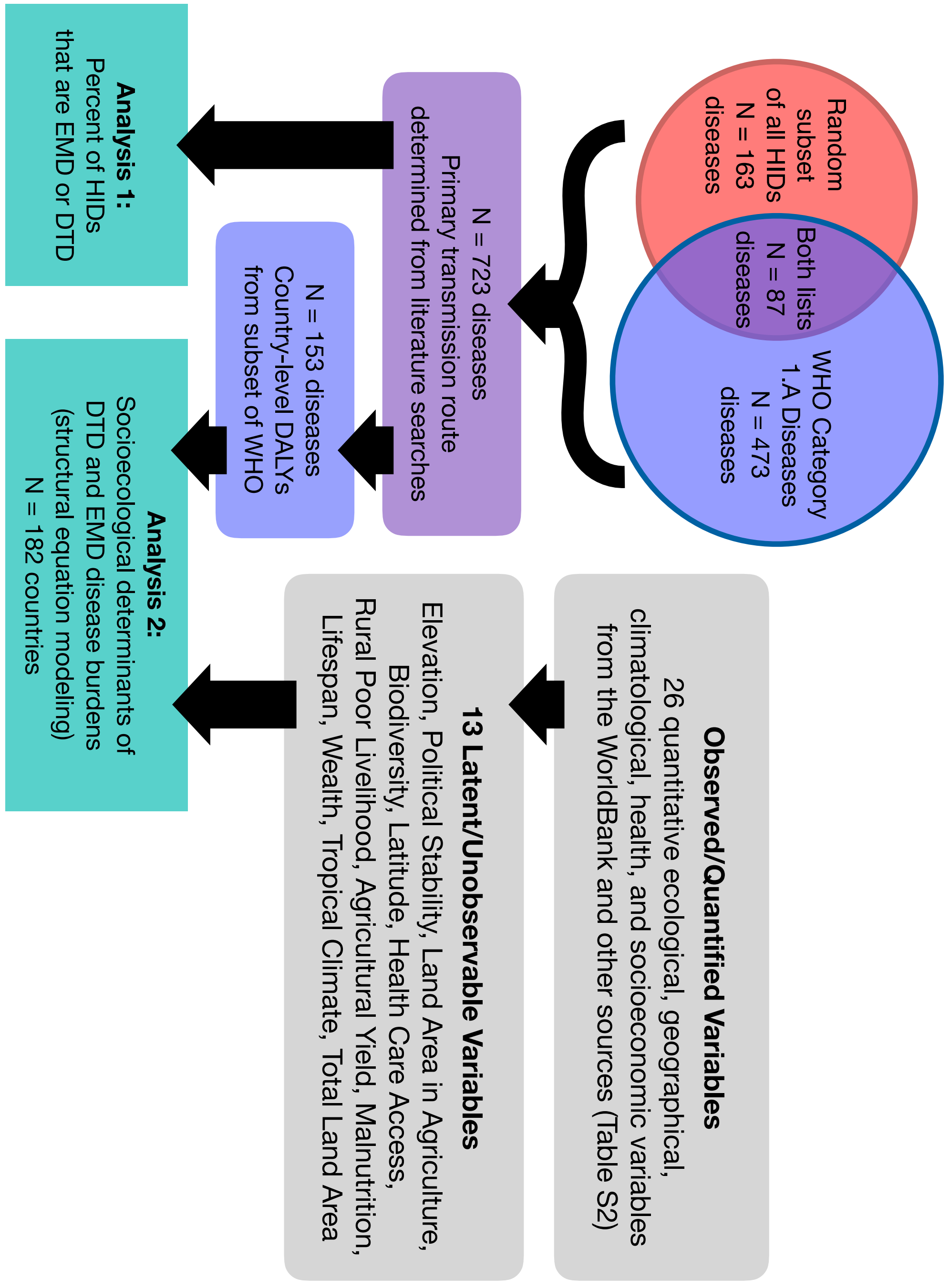

Supplemental Table. Pathogens assessed.

Genus	*Pathogen*	Primary transmission category	Environmentally mediated?	WHO list?	Taylor list?	Refs
Abiotrophia	*Abiotrophia defectiva*	opportunistic/normal flora	no	no	yes	[1,2]
Absidia	*Absidia corymbifera*	sapronosis	yes	yes	yes	[3]
Acanthamoeba	*A astronyxis*	sapronosis	yes	yes	no	[4,5]
Acanthamoeba	*A castellanii*	sapronosis	yes	yes	no	[4,5]
Acanthamoeba	*A culbertsoni*	sapronosis	yes	yes	no	[4,5]
Acanthamoeba	*A hatchetti*	sapronosis	yes	yes	yes	[4,5]
Acanthamoeba	*A palistinensis*	sapronosis	yes	yes	no	[4,5]
Acanthamoeba	*A rhysoides*	sapronosis	yes	yes	no	[4,5]
Acanthamoeba	*Acanthamoeba polyphaga*	sapronosis	yes	yes	yes	[5,6]
Acanthocephalus	*Acanthocephalus bufonis*	foodborne	yes	yes	no	[7]
Acanthocephalus	*Acanthocephalus other*	foodborne	yes	yes	no	[7]
Acanthocephalus	*Acanthocephalus rauschi*	foodborne	yes	yes	no	[7]
Achromobacter	*Achromobacter piechaudii*	unknown/unclassifiable	unknown	no	yes	[8]
Acinetobacter	*Acinetobacter johnsonii*	nosocomial	yes	no	yes	[9]
Acinetobacter	*Acinetobacter junii*	unknown/unclassifiable	unknown	no	yes	[10]
Acinetobacter	*Acinetobacter lwoffii*	human-to-human	no	no	yes	[11]
Acremonium	*Acremonium curvulum*	sapronosis	yes	no	yes	[12,13]
Acremonium	*Acremonium strictum*	sapronosis	yes	no	yes	[14,15]
Actinobacillus	*Actinobacillus equuli*	human-to-human	no	no	yes	[16,17]
Actinomadura	*A latina*	sapronosis	yes	yes	no	[18]
Actinomadura	*Actinomadura madurae*	sapronosis	yes	yes	no	[18]
Actinomadura	*Actinomadura pelletieri*	sapronosis	yes	yes	no	[18]
Actinomyces	*Actinomyces israelii*	opportunistic/normal flora	no	yes	no	[19]
Actinomyces	*Actinomyces meyeri*	opportunistic/normal flora	no	no	yes	[20,21]
Alaria	*Alaria marcianae*	foodborne	yes	no	yes	[22]
Alphacoronavirus	*Human coronavirus 229E*	human-to-human	no	yes	yes	[23]
Alphacoronavirus	*Human coronavirus NL63*	human-to-human	no	yes	no	[23]
Alphainfluenzavirus	*Influenza A virus*	human-to-human	no	no	yes	[24]
Alphavirus	*Chikungunya virus*	vectorborne	yes	yes	yes	[25]
Alphavirus	*Eastern equine encephalitis virus*	vectorborne	yes	yes	no	[26,27]
Alphavirus	*Everglades virus*	vectorborne	yes	no	yes	[28]
Alphavirus	*O'nyong-nyong virus*	vectorborne	yes	yes	no	[29]
Alphavirus	*Ockelbo virus*	vectorborne	yes	no	yes	[30]
Alphavirus	*Ross River virus*	vectorborne	yes	yes	no	[31]
Alphavirus	*Venezuelan equine encephalitis virus*	vectorborne	yes	yes	no	[32]
Alphavirus	*Western equine encephalitis virus*	vectorborne	yes	yes	no	[27,33]
Alternaria	*Alternaria tenuissima*	sapronosis	yes	no	yes	[34,35]
Ancylostoma	*Ancylostoma braziliense*	soilborne	yes	yes	no	[36]
Ancylostoma	*Ancylostoma Caninum*	soilborne	yes	yes	yes	[37]
Ancylostoma	*Ancylostoma ceylanicum*	soilborne	yes	yes	no	[38]
Ancylostoma	*Ancylostoma duodenale*	soilborne	yes	yes	no	[39,40]
Ancylostoma	*Ancylostoma malayanum*	soilborne	yes	yes	no	[7]
Angiostrongylus	*Angiostrongylus cantonensis*	foodborne	yes	yes	no	[41,42]
Anisakis	*A simplex*	foodborne	yes	yes	no	[7]
Anisakis	*Anisakis physeteris*	foodborne	yes	yes	no	[7]
Aphanoascus	*Aphanoascus fulvescens*	sapronosis	yes	no	yes	[43,44]
Apophysomyces	*Apophysomyces elegans*	sapronosis	yes	yes	no	[45]
Arcanobacterium	*Arcanobacterium pyogenes*	directly zoonotic (domestic)	yes	no	yes	[46,47]
Arenavirus	*Junin virus*	directly zoonotic (wildlife)	yes	yes	no	[48,49]
Arenavirus	*Lassa virus*	human-to-human	no	yes	no	[50]
Arenavirus	*Machupo virus*	unknown/unclassifiable	yes	yes	no	[51]
Artyfechinostomum	*Artyfechinostomum mehrai*	foodborne	yes	no	yes	[52]
Ascaris	*Ascaris lumbricoides*	soilborne	yes	yes	no	[53–55]
Aspergillus	*A candidus*	sapronosis	yes	yes	no	[56]
Aspergillus	*A fisherianus*	sapronosis	yes	yes	no	[57]
Aspergillus	*A flavipes*	sapronosis	yes	yes	no	[58]
Aspergillus	*A flavus*	sapronosis	yes	yes	no	[59]
Aspergillus	*A fumigatus group*	sapronosis	yes	yes	no	[60]
Aspergillus	*A glaucus*	sapronosis	yes	yes	no	[61]
Aspergillus	*A nidulans*	sapronosis	yes	yes	no	[62]
Aspergillus	*A niger*	sapronosis	yes	yes	no	[63]
Aspergillus	*A terreus group*	sapronosis	yes	yes	yes	[64,65]
Aspergillus	*A versicolor*	sapronosis	yes	yes	no	[61]
Aspergillus	*A wentii*	sapronosis	yes	yes	no	[66]
Aspergillus	*Aspergillus clavatus*	sapronosis	yes	yes	yes	[67,68]
Australobilharzia	*Australobilharzia terrigalensis*	waterborne	yes	no	yes	[69]

Genus	*Pathogen*	Primary transmission category	Environmentally mediated?	WHO list?	Taylor list?	Refs
Avulavirus	*Newcastle disease virus*	directly zoonotic (domestic)	yes	yes	no	70
Babesia	*B divergens*	vectorborne	yes	yes	no	71
Babesia	*B gibsoni*	vectorborne	yes	yes	no	71
Babesia	*B microti*	vectorborne	yes	yes	no	71
Babesia	*Babesia bovis*	vectorborne	yes	yes	yes	72
Bacillus	*Bacillus anthracis*	soilborne	yes	yes	no	73,74
Bacillus	*Bacillus cereus*	sapronosis	yes	yes	no	75
Bacillus	*Bacillus coagulans*	sapronosis	yes	no	yes	76
Bacillus	*Bacillus fusiformis*	unknown/unclassifiable	unknown	yes	no	77
Bacillus	*Bacillus sphaericus*	unknown/unclassifiable	yes	no	yes	78,79
Bacillus	*Bacillus thuringiensis*	unknown/unclassifiable	yes	no	yes	80
Bacteroides	*Bacteroides fragilis*	opportunistic/normal flora	no	yes	no	81
Bacteroides	*Bacteroides thetaiotaomicron*	opportunistic/normal flora	no	no	yes	82,83
Bacteroides	*Bacteroides vulgata*	opportunistic/normal flora	no	no	yes	84
Balantidium	*Balantidium coli*	waterborne	yes	yes	no	85,86
Bartonella	*Bartonella bacilliformis*	vectorborne	yes	yes	no	87,88
Bartonella	*Bartonella henselae*	vectorborne	yes	yes	no	89
Bartonella	*Bartonella quintana*	vectorborne	yes	yes	no	87,90,91
Basidiobolus	*Basidiobolus ranarum*	sapronosis	yes	yes	no	92
Baylisascaris	*Baylisascaris procyonis*	directly zoonotic (wildlife)	yes	yes	no	93
Beauveria	*Beauveria bassiana*	sapronosis	yes	no	yes	94
Betacoronavirus	*Human coronavirus HKU1*	human-to-human	no	yes	no	23,95
Betacoronavirus	*Human coronavirus OC43*	human-to-human	no	yes	no	23,95
Bipolaris	*Bipolaris australiensis*	sapronosis	yes	no	yes	96,97
Bipolaris	*Bipolaris hawaiiensis*	sapronosis	yes	no	yes	98
Bipolaris	*Bipolaris spicifera*	sapronosis	yes	no	yes	98
Blastomyces	*Blastomyces dermatitidis*	sapronosis	yes	yes	no	99,100
Bordetella	*Bordetella bronchiseptica*	directly zoonotic (domestic)	yes	no	yes	101–103
Bordetella	*Bordetella parapertussis*	human-to-human	no	yes	no	104–106
Bordetella	*Bordetella pertussis*	human-to-human	no	yes	yes	107,108
Bornavirus	*Borna disease virus*	unknown/unclassifiable	unknown	no	yes	109,110
Borrelia	*Borrelia burgdorferei*	vectorborne	yes	yes	no	111,112
Borrelia	*Borrelia hermsii*	vectorborne	yes	yes	yes	113
Borrelia	*Borrelia latyschewii*	vectorborne	yes	no	yes	114
Borrelia	*Borrelia mazzottii*	vectorborne	yes	no	yes	115
Borrelia	*Borrelia recurrentis*	vectorborne	yes	yes	no	116
Borrelia	*Borrelia vincentii*	opportunistic/normal flora	no	yes	no	117
Borrelia	*Borrellia caucasica*	vectorborne	yes	no	yes	118
Botryomyces	*Botryomyces caespitosus*	unknown/unclassifiable	unknown	no	yes	119
Brucella	*Brucella melitensis*	foodborne	yes	yes	yes	120,121
Brugia	*Brugia guyanensis*	vectorborne	yes	no	yes	22
Brugia	*Brugia malayi*	vectorborne	yes	yes	no	122,123
Brugia	*Brugia timori*	vectorborne	yes	yes	no	122,123
Bunostomum	*Bunostomum phlebotomum*	soilborne	yes	no	yes	124,125
Bunyamwera	*Bunyamwera virus*	vectorborne	yes	no	yes	126
Burkholderia	*Burkholderia mallei*	directly zoonotic (domestic)	yes	yes	no	127,128
Burkholderia	*Burkholderia pseudomallei*	sapronosis	yes	yes	no	127,128
Calicivirus	*Norwalk agent*	foodborne/waterborne	yes	yes	no	129,130
Campylobacter	*C concisus*	sapronosis	yes	yes	no	131,132
Campylobacter	*C curvus*	sapronosis	yes	yes	no	131,132
Campylobacter	*C fetus*	sapronosis	yes	yes	no	131,132
Campylobacter	*C gracilis*	sapronosis	yes	yes	no	131,132
Campylobacter	*C hyointestinalis*	sapronosis	yes	yes	no	131,132
Campylobacter	*C jejuni*	sapronosis	yes	yes	no	131,132
Campylobacter	*C lari*	sapronosis	yes	yes	no	131,132
Campylobacter	*C rectus*	sapronosis	yes	yes	no	131,132
Campylobacter	*C sputorum*	sapronosis	yes	yes	no	131,132
Campylobacter	*C upsaliensis*	sapronosis	yes	yes	no	131,132
Campylobacter	*Campylobacter coli*	sapronosis	yes	yes	no	131,132
Candida	*C albicans*	opportunistic/normal flora	no	yes	no	133,134
Candida	*C glabrata*	opportunistic/normal flora	no	yes	no	133
Candida	*C lusitanae*	sapronosis	yes	yes	yes	133
Candida	*C parapsilosis*	sapronosis	yes	yes	no	133,135
Candida	*C tropicalis*	opportunistic/normal flora	no	yes	no	133
Candida	*Candida krusei*	sapronosis	yes	yes	yes	136–138
Capillaria	*Capillaria aerophila*	foodborne	yes	yes	no	7
Capillaria	*Capillaria hepaticum*	foodborne	yes	yes	no	7
Capillaria	*Capillaria philippinensis*	foodborne	yes	yes	no	7
Capnocytophaga	*Capnocytophaga canimorsus*	directly zoonotic (domestic)	yes	no	yes	139,140
Capnocytophaga	*Capnocytophaga ochracea*	opportunistic/normal flora	no	no	yes	141

Genus	*Pathogen*	Primary transmission category	Environmentally mediated?	WHO list?	Taylor list?	Refs
Cardiobacterium	*Cardiobacterium hominis*	opportunistic/normal flora	no	no	yes	[142–144]
Centipeda	*Centipeda periodontii*	opportunistic/normal flora	no	no	yes	[145,146]
Centrocestus	*Centrocestus formosanus*	foodborne	yes	no	yes	[147–149]
Chaetomium	*Chaetomium atrobrunneum*	sapronosis	yes	no	yes	[150,151]
Chlamydia	*Chlamydia psittaci*	directly zoonotic (wildlife)	yes	yes	no	[152,153]
Chlamydia	*Chlamydia trachomatis*	human-to-human	no	yes	no	[154]
Chlamydoabsidia	*Chlamydoabsidia padenii*	unknown/unclassifiable	unknown	no	yes	[155]
Chlorella	*Chlorella protothecoides*	sapronosis	yes	no	yes	[156]
Chryseobacterium	*Chryseobacterium meningo septicum*	nosocomial	yes	no	yes	[157]
Citrobacter	*Citrobacter koseri*	opportunistic/normal flora	no	no	yes	[158,159]
Citrobacter	*Citrobacter sediakii*	human-to-human	no	no	yes	[160]
Citrobacter	*Citrobacter werkmanii*	nosocomial	yes	no	yes	[161]
Cladorrhinum	*Cladorrhinum bulbillosum*	sapronosis	yes	no	yes	[162,163]
Cladosporium	*Cladosporium carrionii*	sapronosis	yes	yes	no	[164]
Clinostomum	*Clinostomum complanatum*	foodborne	yes	no	yes	[165]
Clostridium	*Clostridium bifermentans*	unknown/unclassifiable	unknown	no	yes	[166]
Clostridium	*Clostridium botulinum*	sapronosis	yes	yes	no	[167]
Clostridium	*Clostridium chauvoei*	unknown/unclassifiable	unknown	no	yes	[166]
Clostridium	*Clostridium difficile*	human-to-human	unknown	yes	no	[168,169]
Clostridium	*Clostridium novyi*	opportunistic/normal flora	yes	no	yes	[166]
Clostridium	*Clostridium perfringens*	sapronosis	yes	yes	yes	[170,171]
Clostridium	*Clostridium sordellii*	opportunistic/normal flora	yes	no	yes	[166]
Clostridium	*Clostridium tetani*	sapronosis	yes	yes	no	[172,173]
Cocciodiodes	*Coccidioides immitis*	sapronosis	yes	yes	no	[174]
Cokeromyces	*Cokeromyces recurvartus*	sapronosis	yes	yes	no	[175]
Coltivirus	*Colorado tick fever virus*	vectorborne	yes	yes	no	[176]
Coltivirus	*Eyach virus*	vectorborne	yes	no	yes	[177]
Conidiobolus	*C incongruus*	sapronosis	yes	yes	no	[178]
Conidiobolus	*C lamprauges*	sapronosis	yes	yes	no	[179]
Conidiobolus	*Conidiobolus coronatus*	sapronosis	yes	yes	no	[180,181]
Corynebacterium	*Corynebacterium diphtheriae*	human-to-human	no	yes	no	[182]
Corynebacterium	*Corynebacterium minutissimum*	unknown/unclassifiable	no	no	yes	[183]
Corynebacterium	*Corynebacterium pseudodipthericum*	unknown/unclassifiable	no	no	yes	[184–186]
Corynebacterium	*Corynebacterium striatum*	opportunistic/normal flora	no	no	yes	[187,188]
Corynosoma	*Corynosoma strumosum*	foodborne	yes	no	yes	[189]
Coxiella	*Coxiella burnetii*	vectorborne	yes	yes	no	[190,191]
Cryptococcus	*Cryptococcus neoformans*	sapronosis	yes	yes	no	[192]
Cryptosporidium	*Cryptosporidium canis*	waterborne	yes	yes	no	[193–195]
Cryptosporidium	*Cryptosporidium felis*	waterborne	yes	yes	no	[193–195]
Cryptosporidium	*Cryptosporidium hominis*	waterborne	yes	yes	no	[193–195]
Cryptosporidium	*Cryptosporidium meleagradis*	waterborne	yes	yes	no	[193–195]
Cryptosporidium	*Cryptosporidium parvum*	waterborne	yes	yes	no	[193–195]
Cunninghamella	*Cunninghamella bertholletiae*	sapronosis	yes	yes	no	[196]
Curvularia	*Curvularia geniculata*	sapronosis	yes	yes	yes	[197]
Curvularia	*Curvularia lunata*	sapronosis	yes	yes	no	[198]
Cyclodontostomum	*Cyclodontostomum purvisi*	foodborne	yes	no	yes	[199–201]
Cyclospora	*Cyclospora cayetanensis*	foodborne	yes	no	yes	[202]
Cylindrocarpon	*Cylindrocarpon destructans*	sapronosis	yes	yes	no	[203]
Cylindrocarpon	*Cylindrocarpon cyanescens*	sapronosis	yes	yes	no	[203]
Cytomegalovirus	*Human herpesvirus 5*	human-to-human	no	yes	no	[204]
Delftia	*Delftia acidovorans*	sapronosis	yes	no	yes	[205,206]
Deltaretrovirus	*Human T-Lymphotrophic virus 1*	human-to-human	no	yes	no	[207]
Dicrocoelium	*Dicrocoelium dendriticum*	foodborne/waterborne	yes	yes	no	[208,209]
Diphyllobothrium	*D cameroni*	foodborne	yes	yes	no	[7]
Diphyllobothrium	*D cordatum*	foodborne	yes	yes	no	[7]
Diphyllobothrium	*D dalliae*	foodborne	yes	yes	no	[7]
Diphyllobothrium	*D dendriticum*	foodborne	yes	yes	no	[7]
Diphyllobothrium	*D elegans*	foodborne	yes	yes	no	[7]
Diphyllobothrium	*D erinaceieuropaei*	foodborne	yes	yes	no	[7]
Diphyllobothrium	*D hians*	foodborne	yes	yes	no	[7]
Diphyllobothrium	*D houghtoni*	foodborne	yes	yes	no	[7]
Diphyllobothrium	*D klebanovskii*	foodborne	yes	yes	no	[7]
Diphyllobothrium	*D lanceolatum*	foodborne	yes	yes	no	[7]
Diphyllobothrium	*D mansonoides*	foodborne	yes	yes	no	[7]
Diphyllobothrium	*D nihonkaiense*	foodborne	yes	yes	no	[7]
Diphyllobothrium	*D orcini*	foodborne	yes	yes	no	[7]
Diphyllobothrium	*D pacificum*	foodborne	yes	yes	no	[210]
Diphyllobothrium	*D scoticum*	foodborne	yes	yes	no	[7]
Diphyllobothrium	*D stemmacephalum*	foodborne	yes	yes	no	[7]
Diphyllobothrium	*D theileri*	foodborne	yes	yes	no	[7]

Genus	*Pathogen*	Primary transmission category	Environmentally mediated?	WHO list?	Taylor list?	Refs
Diphyllobothrium	*Diphyllobothrium latum*	foodborne	yes	yes	no	210
Dipylidium	*Dipylidium caninum*	foodborne	yes	yes	no	7
Dirofilaria	*D immitis*	vectorborne	yes	yes	no	7
Dirofilaria	*D repens*	vectorborne	yes	yes	no	7
Dirofilaria	*D striata*	vectorborne	yes	yes	no	7
Dirofilaria	*D subdermata*	vectorborne	yes	yes	yes	211,212
Dirofilaria	*D ursi*	vectorborne	yes	yes	no	7
Dirofilaria	*Dirofilaria tenuis*	vectorborne	yes	yes	yes	213
Doratomyces	*Doratomyces stemonitis*	unknown/unclassifiable	yes	no	yes	214,215
Dracunculus	*Dracunculus insignis*	waterborne	yes	yes	no	7
Dracunculus	*Dracunculus medinensis*	waterborne	yes	yes	no	7
Drepanidotaenia	*Drepanidotaenia lanceolata*	foodborne/waterborne	yes	no	yes	216
Ebolavirus	*Cote d'Ivoire ebolavirus*	human-to-human	no	yes	no	217
Ebolavirus	*Reston ebolavirus*	human-to-human	no	yes	no	218
Ebolavirus	*Sudan ebolavirus*	human-to-human	no	yes	yes	217
Ebolavirus	*Zaire ebolavirus*	human-to-human	no	yes	no	217
Echinococcus	*Echinococcus granulosus*	foodborne	yes	yes	no	7
Echinococcus	*Echinococcus multilocularis*	foodborne	yes	yes	no	7
Echinococcus	*Echinococcus oligarthus*	foodborne	yes	yes	no	7
Echinococcus	*Echinococcus vogeli*	foodborne	yes	yes	no	7
Echinoparyphium	*Echinoparyphium recurvatum*	foodborne	yes	no	yes	219
Echinostoma	*E cinetorchis*	foodborne	yes	yes	no	220
Echinostoma	*E echinatum*	foodborne	yes	yes	no	221
Echinostoma	*E hortense*	foodborne	yes	yes	no	222
Echinostoma	*E jassyense*	foodborne	yes	yes	yes	149
Echinostoma	*E macrorchis*	foodborne	yes	yes	no	223
Echinostoma	*E malayanum*	foodborne	yes	yes	no	224
Echinostoma	*E revolutum*	foodborne	yes	yes	no	225
Echinostoma	*Echinostoma ilocanum*	foodborne	yes	yes	yes	226
Ehrlichia	*Ehrlichia chaffeensis*	vectorborne	yes	no	yes	227
Ehrlichia	*Ehrlichia equi*	vectorborne	yes	no	yes	228
Ehrlichia	*Ehrlichia sennetsu*	foodborne	yes	yes	no	229
Emmonsia	*E crescens*	sapronosis	yes	yes	no	230
Emmonsia	*Emmonsia parva*	sapronosis	yes	yes	yes	231
Encephalitozoon	*E cuniculi*	sapronosis	yes	yes	no	232–234
Encephalitozoon	*E intestinalis*	sapronosis	yes	yes	no	232,233
Encephalitozoon	*Encephalitozoon hellem*	sapronosis	yes	yes	no	7,232,23
Entamoeba	*Entamoeba histolytica*	sapronosis	yes	yes	no	235
Enterobacter	*Enterobacter amnigenus*	unknown/unclassifiable	yes	no	yes	236
Enterobacter	*Enterobacter cloacae*	sapronosis	yes	no	yes	237
Enterobius	*E gregorii*	human-to-human	no	yes	no	7
Enterobius	*Enterobius vermicularis*	human-to-human	no	yes	no	7,238
Enterococcus	*Enterococcus durans*	unknown/unclassifiable	yes	no	yes	239
Enterococcus	*Enterococcus faeclum*	unknown/unclassifiable	yes	no	yes	239–241
Enterococcus	*Entrococcus avium*	opportunistic/normal flora	no	no	yes	239
Enterocytozoon	*Enterocytozoon bieneusi*	unknown/unclassifiable	yes	yes	yes	242
Enterovirus	*Echovirus*	foodborne/waterborne	yes	yes	no	243,244
Enterovirus	*Echovirus type 16*	human-to-human	no	yes	no	245
Enterovirus	*Human enterovirus 70*	human-to-human	no	yes	no	246
Enterovirus	*Human enterovirus A*	human-to-human	no	yes	no	246
Enterovirus	*Human enterovirus B*	human-to-human	no	yes	no	246,247
Enterovirus	*Poliovirus*	human-to-human	no	yes	no	248
Epidermophyton	*Epidermophyton floccosum*	human-to-human	no	yes	no	249
Erysipelothrix	*Erysipelothrix rhusiopathiae*	sapronosis	yes	yes	no	250,251
Escherichia	*Escherichia coli*	sapronosis	yes	yes	no	252,253
Eubacterium	*Eubacterium brachy*	opportunistic/normal flora	no	no	yes	254,255
Eubacterium	*Eubacterium combesii*	opportunistic/normal flora	no	no	yes	256
Eubacterium	*Eubacterium contortum*	opportunistic/normal flora	no	no	yes	257
Exophalia	*E oligosperma*	sapronosis	yes	yes	no	258,259
Exophalia	*Exophalia jeanselmei*	sapronosis	yes	yes	no	260,261
Fasciola	*F hepatica*	foodborne	yes	yes	no	262
Fasciola	*F indica*	foodborne	yes	yes	no	262
Fasciola	*Fasciola gigantica*	foodborne	yes	yes	no	262
Fasciolopsis	*Fasciolopsis buski*	foodborne	yes	yes	no	263
Fibrobacter	*Fibrobacter intestinalis*	opportunistic/normal flora	no	no	yes	264
Flavivirus	*Central European encephalitis virus*	vectorborne	yes	yes	no	265
Flavivirus	*Dengue virus*	vectorborne	yes	yes	yes	266,267
Flavivirus	*Far eastern tickborne encephalitis virus*	vectorborne	yes	yes	yes	268
Flavivirus	*Japanese encephalitis virus*	vectorborne	yes	yes	no	269
Flavivirus	*Kunjin virus*	vectorborne	yes	yes	no	270

Genus	*Pathogen*	Primary transmission category	Environmentally mediated?	WHO list?	Taylor list?	Refs
Flavivirus	*Kyasanur forest disease virus*	vectorborne	yes	yes	yes	[271]
Flavivirus	*Louping ill virus*	vectorborne	yes	yes	no	[272,273]
Flavivirus	*Murray Valley virus*	vectorborne	yes	yes	no	[274]
Flavivirus	*Omsk haemorrhagic fever virus*	vectorborne	yes	yes	no	[275]
Flavivirus	*Powassan virus*	vectorborne	yes	yes	no	[276]
Flavivirus	*Rocio virus*	vectorborne	yes	yes	no	[277]
Flavivirus	*St Louis encephalitis virus*	vectorborne	yes	yes	no	[278]
Flavivirus	*West Nile virus*	vectorborne	yes	yes	no	[279]
Flavivirus	*Yellow fever virus*	vectorborne	yes	yes	no	[280]
Fluoribacter	*Fluoribacter bozemanae (Legionella*	sapronosis	yes	no	yes	[281]
Fluoribacter	*Fluoribacter dumoffi (Legionella dumoffi)*	sapronosis	yes	no	yes	[282]
Fonsecaea	*Fonsecaea compacta (aka Rhinocladiella*	sapronosis	yes	yes	no	[283]
Fonsecaea	*Fonsecaea pedrosoi*	sapronosis	yes	yes	no	[284]
Francisella	*Francisella tularensis*	vectorborne	yes	yes	yes	[285–287]
Fusarium	*Fusarium solani*	sapronosis	yes	yes	no	[288]
Fusarium	*Fusarium verticillioides*	sapronosis	yes	yes	no	[289]
Fusobacterium	*Fusobacterium mortiferum*	opportunistic/normal flora	no	no	yes	[290]
Gardnerella	*Gardnerella vaginalis*	human-to-human	no	no	yes	[291]
Gastrodiscoides	*Gastrodiscoides hominis*	foodborne	yes	no	yes	[292]
Geotrichum	*Geotrichum candidum*	sapronosis	yes	yes	no	[293,294]
Giardia	*Giardia lamblia*	waterborne	yes	yes	yes	[295]
Gnathostoma	*G hispidum*	foodborne	yes	yes	no	[7]
Gnathostoma	*G nipponicum*	foodborne	yes	yes	no	[7]
Gnathostoma	*Gnathostoma doloresi*	foodborne	yes	yes	yes	[296,297]
Gnathostoma	*Gnathostoma spinigerum*	waterborne	yes	yes	yes	[298,299]
Gonglyonema	*Gonglyonema pulchrum*	foodborne/waterborne	yes	yes	no	[300]
Gordonia	*Gordonia terrae*	sapronosis	yes	no	yes	[301]
Haemophilus	*Haemophilus aphrophilus*	opportunistic/normal flora	no	no	yes	[302–304]
Haemophilus	*Haemophilus ducreyi*	human-to-human	no	yes	no	[305,306]
Haemophilus	*Haemophilus influenzae*	human-to-human	no	yes	no	[307]
Hantavirus	*Andes virus*	directly zoonotic (wildlife)	yes	no	yes	[308]
Hantavirus	*Dobrava virus*	directly zoonotic (wildlife)	yes	yes	no	[309]
Hantavirus	*Hantaan virus*	directly zoonotic (wildlife)	yes	yes	no	[38,309,3]
Hantavirus	*Puumala virus*	directly zoonotic (wildlife)	yes	yes	no	[309]
Hantavirus	*Seoul virus*	directly zoonotic (wildlife)	yes	yes	no	[309]
Hantavirus	*Sin Nombre virus*	directly zoonotic (wildlife)	yes	yes	no	[311]
Haplorchis	*Haplorchis vanissima*	foodborne	yes	no	yes	[312]
Helicobacter	*Helicobacter pylori*	human-to-human	no	yes	no	[313,314]
Hepatovirus	*Hepatitis A virus*	human-to-human	no	yes	no	[315]
Heterophyes	*H dispar*	foodborne	yes	yes	no	[316]
Heterophyes	*H nocens*	foodborne	yes	yes	no	[317]
Heterophyes	*Heterophyes heterophyes*	foodborne	yes	yes	no	[316]
Heterophyopsis	*Heterophyopsis continua*	foodborne	yes	no	yes	[318]
Histoplasma	*Histoplasma capsulatum*	sapronosis	yes	yes	no	[319]
Hortaea	*Hortaea werneckii*	sapronosis	yes	yes	no	[320]
Hymenolepis	*Hymenolepis diminuta*	foodborne	yes	yes	no	[7]
Hymenolepis	*Hymenolepis nana*	foodborne	yes	yes	no	[7]
Isospora	*Isospora belli*	foodborne/waterborne	yes	yes	yes	[321,322]
Isospora	*Isospora hominis*	foodborne/waterborne	yes	yes	no	[323]
Klebsiella	*Klebsiella granulomatis*	human-to-human	no	yes	no	[324,325]
Klebsiella	*Klebsiella oxytoca*	nosocomial	yes	no	yes	[326]
Klebsiella	*Klebsiella pneumoniae*	sapronosis	yes	yes	no	[327]
Lacazia	*Lacazia loboi*	sapronosis	yes	yes	no	[328,329]
Lechiguanas	*Lechiguanas virus*	directly zoonotic (wildlife)	yes	no	yes	[330]
Legionella	*Legionella cincinnatiensis*	sapronosis	yes	no	yes	[331]
Legionella	*Legionella lansingensis*	sapronosis	yes	no	yes	[332]
Legionella	*Legionella oakridgensis*	sapronosis	yes	no	yes	[333]
Legionella	*Legionella pneumophila*	sapronosis	yes	yes	yes	[334,335]
Leishmania	*Leishmania aethiopica*	vectorborne	yes	yes	yes	[336]
Leishmania	*Leishmania amazonensis*	vectorborne	yes	yes	no	[336]
Leishmania	*Leishmania braziliensis*	vectorborne	yes	yes	no	[336]
Leishmania	*Leishmania donovani*	vectorborne	yes	yes	no	[336]
Leishmania	*Leishmania guyanensis*	vectorborne	yes	yes	no	[336]
Leishmania	*Leishmania infantum*	vectorborne	yes	yes	no	[336]
Leishmania	*Leishmania lainsoni*	vectorborne	yes	yes	yes	[308]
Leishmania	*Leishmania major*	vectorborne	yes	yes	yes	[7,337,33]
Leishmania	*Leishmania mexicana*	vectorborne	yes	yes	no	[336]
Leishmania	*Leishmania naiffi*	vectorborne	yes	yes	yes	[339]
Leishmania	*Leishmania panamensis*	vectorborne	yes	yes	no	[336]

**Genus**	***Pathogen***	**Primary transmission category**	**Environmentally mediated?**	**WHO list?**	**Taylor list?**	**Refs**
Leishmania	*Leishmania peruviana*	vectorborne	yes	yes	no	[336]
Leishmania	*Leishmania tropica*	vectorborne	yes	yes	no	[336]
Leishmania	*Leishmania venezuelensis*	vectorborne	yes	yes	yes	[340–342]
Lentivirus	*Human immunodeficiency virus 1*	human-to-human	no	yes	no	[343]
Lentivirus	*Human immunodeficiency virus 2*	human-to-human	no	yes	no	[343]
Leptospaeria	*Leptospaeria senegalensis*	sapronosis	yes	no	yes	[344]
Leptosphaeria	*Leptosphaeria senegalensis*	sapronosis	yes	yes	no	[345]
Leptosphaeria	*Leptosphaeria tompkinsii*	sapronosis	yes	yes	no	[345]
Leptospira	*L inadai*	waterborne	yes	yes	no	[346]
Leptospira	*L kirschneri*	waterborne	yes	yes	no	[346347,3]
Leptospira	*L meyeri*	waterborne	yes	yes	no	[346]
Leptospira	*L noguchii*	waterborne	yes	yes	no	[346]
Leptospira	*L peruviana*	waterborne	yes	yes	no	[346]
Leptospira	*L weilii*	waterborne	yes	yes	no	[346]
Leptospira	*Leptospira borgpetersenii*	waterborne	yes	yes	yes	[347,348]
Leptospira	*Leptospira interrogans*	waterborne	yes	yes	yes	[349–351]
Leptospira	*Leptospira santarosaii*	waterborne	yes	yes	yes	[352,353]
Listeria	*Listeria monocytogenes*	sapronosis	yes	yes	no	[354]
Listeria	*Listeria weishimeri*	foodborne	yes	no	yes	[355,356]
Loa	*Loa loa*	vectorborne	yes	yes	no	[7]
Lymphocryptovirus	*Epstein–Barr virus*	human-to-human	no	yes	no	[357]
Lyssavirus	*Mokola virus*	directly zoonotic (wildlife)	unknown	no	yes	[358]
Lyssavirus	*Rabies virus*	directly zoonotic (wildlife)	yes	yes	no	[359]
Madurella	*M grisea*	sapronosis	yes	yes	no	[360,361]
Madurella	*Madurella mycetomatis*	sapronosis	yes	yes	no	[360,361]
Malassezia	*M globosa*	opportunistic/normal flora	no	yes	no	[362]
Malassezia	*M obtusa*	opportunistic/normal flora	no	yes	no	[362]
Malassezia	*M pachydermatis*	opportunistic/normal flora	no	yes	no	[362]
Malassezia	*M restricta*	opportunistic/normal flora	no	yes	no	[362]
Malassezia	*M sloofiae*	opportunistic/normal flora	no	yes	no	[362]
Malassezia	*M sympodialis*	opportunistic/normal flora	no	yes	no	[362]
Malassezia	*Malassezia furfur*	opportunistic/normal flora	unknown	yes	yes	[362–364]
Mammarenavirus	*Lymphocytic choriomeningitis virus*	directly zoonotic (wildlife)	yes	yes	no	[365]
Mammomonogamou	*Mammomonogamous laryngeus*	unknown/unclassifiable	yes	yes	no	[7]
Mammomonogamou	*Mammomonogamous nasicola*	unknown/unclassifiable	yes	yes	no	[7]
Mansonella	*Mansonella ozzardi*	vectorborne	yes	yes	no	[7]
Mansonella	*Mansonella perstans*	vectorborne	yes	yes	no	[7]
Mansonella	*Mansonella rodhaini*	vectorborne	yes	yes	no	[7]
Mansonella	*Mansonella semiclarum*	vectorborne	yes	yes	no	[7]
Mansonella	*Mansonella streptocerca*	vectorborne	yes	yes	no	[7]
Marburgvirus	*Marburg virus*	directly zoonotic (wildlife)	yes	yes	no	[366,367]
Mastadenovirus	*Human adenovirus A*	human-to-human	no	yes	no	[368,369]
Mastadenovirus	*Human adenovirus B*	human-to-human	no	yes	no	[368,369]
Mastadenovirus	*Human adenovirus C*	human-to-human	no	yes	no	[368,369]
Mastadenovirus	*Human adenovirus D*	human-to-human	no	yes	no	[368,369]
Mastadenovirus	*Human adenovirus E*	human-to-human	no	yes	no	[368,369]
Mastadenovirus	*Human adenovirus F*	human-to-human	no	yes	no	[368,369]
Mathevotaenia	*Mathevotaenia symmetrica*	unknown/unclassifiable	yes	no	yes	[216,370,]
Mecistocirrus	*Mecistocirrus digitatus*	foodborne	yes	no	yes	[372,373]
Metagonimus	*M minutus*	foodborne	yes	yes	no	[7]
Metagonimus	*Metagonimus yokogawai*	foodborne	yes	yes	yes	[7]
Metapneumovirus	*Human metapneumovirus*	human-to-human	no	yes	no	[374]
Metastrongylus	*Metastrongylus elongatus*	foodborne	yes	yes	no	[7]
Metorchis	*Metorchis conjunctus*	foodborne	yes	no	yes	[375]
Microascus	*Microascus cinereus*	sapronosis	yes	no	yes	[151,376]
Microascus	*Microascus cirrosus*	sapronosis	yes	no	yes	[377]
Micronema	*Micronema deletrix*	sapronosis	yes	no	yes	[378]
Microsporum	*M canis*	sapronosis	yes	yes	no	[249]
Microsporum	*M equinum*	sapronosis	yes	yes	no	[249,379]
Microsporum	*M ferrugineum*	human-to-human	no	yes	no	[249]
Microsporum	*M fulvum*	sapronosis	yes	yes	no	[249]
Microsporum	*M gallinae*	sapronosis	yes	yes	no	[249]
Microsporum	*M gypseum*	sapronosis	yes	yes	no	[249]
Microsporum	*M nanum*	sapronosis	yes	yes	no	[249]
Microsporum	*M persicolor*	sapronosis	yes	yes	no	[249,380]
Microsporum	*M praecox*	sapronosis	yes	yes	no	[249]
Microsporum	*M racemosum*	sapronosis	yes	yes	no	[249]
Microsporum	*M vanbreuseghemii*	sapronosis	yes	yes	no	[249]
Microsporum	*Microsporum audouinii*	human-to-human	no	yes	yes	[381,382]
Molluscipoxvirus	*Molluscum contagiosum virus*	human-to-human	no	yes	yes	[383]

Genus	*Pathogen*	Primary transmission category	Environmentally mediated?	WHO list?	Taylor list?	Refs
Moniezia	*Moniezia expansa*	foodborne	yes	no	yes	384
Moniliella	*Moniliella suavelolens*	sapronosis	unknown	no	yes	385
Moraxella	*Moraxella cuniculi*	unknown/unclassifiable	unknown	no	yes	386–388
Morbillivirus	*Measles virus*	human-to-human	no	yes	yes	389
Mucor	*M circinelloides*	sapronosis	yes	yes	no	390
Mucor	*M indicus*	sapronosis	yes	yes	no	391
Mucor	*M racemosus*	sapronosis	yes	yes	no	
Mucor	*M ramosissimus*	sapronosis	yes	yes	no	
Mucor	*Mucor hiemalis*	sapronosis	yes	yes	yes	392
Multiceps	*Multiceps brauni*	foodborne	yes	no	yes	393
Myceliophthora	*Myceliophthora thermophila*	sapronosis	yes	no	yes	394
Mycobacterium	*M abscessus*	sapronosis	yes	yes	yes	395–397
Mycobacterium	*M asiaticum*	unknown/unclassifiable	yes	yes	no	398
Mycobacterium	*M celatum*	sapronosis	yes	yes	no	399,400
Mycobacterium	*M chelonae*	waterborne	yes	yes	yes	401–403
Mycobacterium	*M conspicuum*	unknown/unclassifiable	unknown	yes	no	404
Mycobacterium	*M fortuitum*	sapronosis	yes	yes	no	366
Mycobacterium	*M genavense*	unknown/unclassifiable	unknown	yes	no	400
Mycobacterium	*M gordonae*	sapronosis	yes	yes	yes	405
Mycobacterium	*M haemophilium*	unknown/unclassifiable	yes	yes	no	400
Mycobacterium	*M intracellulare*	sapronosis	yes	yes	no	400
Mycobacterium	*M kansasii*	sapronosis	yes	yes	no	400,406,
Mycobacterium	*M malmoense*	sapronosis	yes	yes	no	400,408
Mycobacterium	*M marinum*	sapronosis	yes	yes	yes	405,409
Mycobacterium	*M mucogenicum*	sapronosis	yes	yes	yes	405
Mycobacterium	*M peregrinum*	sapronosis	yes	yes	no	400,410
Mycobacterium	*M porcinum*	sapronosis	yes	yes	no	400,411
Mycobacterium	*M scrofulaceum*	sapronosis	yes	yes	no	400
Mycobacterium	*M senegalese*	sapronosis	yes	yes	yes	405,412,
Mycobacterium	*M shimoidei*	unknown/unclassifiable	unknown	yes	yes	414,415
Mycobacterium	*M simium*	directly zoonotic (wildlife)	yes	yes	no	400
Mycobacterium	*M smegmatis*	sapronosis	yes	yes	no	400,416
Mycobacterium	*M szulgai*	unknown/unclassifiable	yes	yes	yes	417
Mycobacterium	*M ulcerans*	sapronosis	yes	yes	no	400,418
Mycobacterium	*M xenopi*	sapronosis	yes	yes	no	419,420
Mycobacterium	*Mycobacterium avium*	sapronosis	yes	yes	yes	421–423
Mycobacterium	*Mycobacterium bovis*	foodborne	yes	yes	no	366,424
Mycobacterium	*Mycobacterium leprae*	human-to-human	no	yes	no	425–427
Mycobacterium	*Mycobacterium tuberculosis*	human-to-human	no	yes	no	428,429
Mycocentrospora	*Mycocentrospora acerina*	sapronosis	yes	no	yes	430
Mycoplasma	*Mycoplasma pneumoniae*	human-to-human	no	yes	no	431
Naegleria	*Naegleria fowleri*	sapronosis	yes	yes	no	432
Nairovirus	*Crimean-Congo hemorrhagic fever virus*	vectorborne	yes	yes	yes	433
Nannizzia	*Nannizzia cajetani*	sapronosis	yes	no	yes	434
Nanophyetus	*Nanophyetus salmincola*	foodborne	yes	yes	no	7
Nattrassia	*Nattrassia mangiferae*	sapronosis	yes	no	yes	435
Necator	*Necator americanus*	soilborne	yes	yes	no	53–55
Neisseria	*Neisseria gonorrhoeae*	human-to-human	no	yes	no	436
Neisseria	*Neisseria meningitidis*	human-to-human	no	yes	yes	437–439
Neisseria	*Neisseria sicca*	opportunistic/normal flora	no	no	yes	440
Neotestudina	*Neotestudina rosatii*	sapronosis	yes	yes	no	441
Nocardia	*Nocardia asteroides*	sapronosis	yes	yes	no	129
Nocardia	*Nocardia brasiliensis*	sapronosis	yes	yes	yes	442,443
Nocardia	*Nocardia farcinica*	sapronosis	yes	yes	no	129,444
Nocardia	*Nocardia nova*	sapronosis	yes	yes	no	129
Nocardia	*Nocardia otitidiscaviarum*	sapronosis	yes	yes	no	129
Nosema	*N africanum*	sapronosis	yes	yes	no	7,233,44
Nosema	*N ceylonensis*	sapronosis	yes	yes	no	7,233,44
Nosema	*N ocularum*	sapronosis	yes	yes	no	7,233,44
Nosema	*Nosema connori*	unknown/unclassifiable	yes	yes	no	7,233,44
Oesophagostomum	*O aculeatum*	unknown/unclassifiable	unknown	yes	no	7
Oesophagostomum	*O bifurcum*	foodborne	yes	yes	no	7,446
Oesophagostomum	*Oesophagostomum stephanostomum*	foodborne	yes	yes	yes	447,448
Oidiodendron	*Oidiodendron cerealis*	sapronosis	yes	no	yes	449
Onchocerca	*Onchocerca volvulus*	vectorborne	yes	yes	no	450
Oncovirus	*Oncovirus*	human-to-human	no	yes	no	451
Opisthorchis	*O sinensis*	foodborne	yes	yes	no	452
Opisthorchis	*O viverrini*	foodborne	yes	yes	no	452
Opisthorchis	*Opisthorchis felineus*	foodborne	yes	yes	yes	452
Orthobunyavirus	*California encephalitis virus*	vectorborne	yes	yes	no	453

Genus	*Pathogen*	Primary transmission category	Environmentally mediated?	WHO list?	Taylor list?	Refs
Orthobunyavirus	*Gan gan virus*	vectorborne	yes	no	yes	454
Orthobunyavirus	*La Crosse virus*	vectorborne	yes	yes	no	455
Orthobunyavirus	*Marituba virus*	vectorborne	yes	no	yes	456
Orthobunyavirus	*Oropouche virus*	vectorborne	yes	yes	no	457
Orthohantavirus	*New York virus*	directly zoonotic (wildlife)	yes	no	yes	458
Orthohepadnavirus	*Hepatitis B virus*	human-to-human	no	yes	no	459
Orthohepadnavirus	*Hepatitis C virus*	human-to-human	no	yes	no	460
Orthonairovirus	*Tamdy virus*	vectorborne	yes	no	yes	461
Orthopneumovirus	*Respiratory syncytial virus*	human-to-human	no	yes	no	462
Orthopoxvirus	*Cowpox virus*	directly zoonotic (wildlife)	yes	yes	no	463
Orthopoxvirus	*Monkeypox virus*	directly zoonotic (wildlife)	yes	yes	no	464
Ovadendron	*Ovadendron ochraceum*	sapronosis	yes	no	yes	465,466
Paenibacillus	*Paenibacillus alvei*	sapronosis	yes	no	yes	251,467
Papillomavirus	*Human papillomavirus*	human-to-human	no	yes	no	468
Paracoccidioides	*Paracoccidioides brasiliensis*	sapronosis	yes	yes	no	469
Paragonimus	*P africanus*	foodborne	yes	yes	no	470
Paragonimus	*P caliensis*	foodborne	yes	yes	no	471
Paragonimus	*P heterotremus*	foodborne	yes	yes	no	472
Paragonimus	*P hueit'ungensis*	foodborne	yes	yes	no	473
Paragonimus	*P kellicotti*	foodborne	yes	yes	yes	474
Paragonimus	*P mexicanus*	foodborne	yes	yes	no	475
Paragonimus	*P miyazakii*	foodborne	yes	yes	no	476
Paragonimus	*P ohirai*	foodborne	yes	yes	no	477
Paragonimus	*P phillipinensis*	foodborne	yes	yes	no	478
Paragonimus	*P sadoensis*	foodborne	yes	yes	no	479
Paragonimus	*P siamensis*	foodborne	yes	yes	no	480
Paragonimus	*P skrjabini*	foodborne	yes	yes	no	481
Paragonimus	*P uterobilateralis*	foodborne	yes	yes	no	482
Paragonimus	*P westermani*	foodborne	yes	yes	no	483
Paragonimus	*P bangkokensis*	foodborne	yes	yes	yes	484
Parapoxvirus	*Orf virus*	directly zoonotic (domestic)	yes	yes	no	485
Parapoxvirus	*Pseudocowpox virus*	directly zoonotic (domestic)	yes	yes	no	486,487
Parastrongylus	*Parastrongylus cantonensis*	foodborne	yes	yes	no	7
Parastrongylus	*Parastrongylus costaricensis*	foodborne	yes	yes	no	7
Parvovirus	*Human parvovirus B19*	human-to-human	no	yes	no	488
Pasteurella	*Pasteurella canis (may be syn P multocida)*	directly zoonotic (domestic)	yes	no	yes	489,490
Pasteurella	*Pasteurella dagmatis*	unknown/unclassifiable	yes	no	yes	491
Pasteurella	*Pasteurella multocida*	directly zoonotic (domestic)	no	yes	no	489,490
Pearsonema	*Pearsonema plica*	unknown/unclassifiable	yes	no	yes	492
Pediculus	*Pediculus capitis*	human-to-human	no	yes	no	7
Pediculus	*Pediculus humanus*	human-to-human	no	yes	no	7
Pegivirus	*Hepatitis G virus*	human-to-human	no	no	yes	493
Penicillium	*Penicillium decumbens*	sapronosis	yes	no	yes	494
Penicillum	*Penicillum marneffei*	sapronosis	yes	yes	yes	494
Phaeoaneliomyces	*Phaeoaneliomyces elegans*	sapronosis	yes	no	yes	495
Phialemonium	*Phialemonium obovatum*	unknown/unclassifiable	yes	no	yes	496,497
Phialophora	*Phialophora verrucosa*	sapronosis	yes	yes	no	498
Phlebovirus	*Rift Valley fever virus*	vectorborne	yes	yes	no	499
Phlebovirus	*Sandfly fever virus*	vectorborne	yes	yes	yes	500
Phlebovirus	*Zinga virus*	vectorborne	yes	yes	yes	501
Phoma	*Phoma cava*	sapronosis	yes	no	yes	502
Phthrius	*Phthrius pubis*	human-to-human	no	yes	no	7
Piedraia	*Piedraia hortae*	sapronosis	yes	yes	no	503
Plagiorchis	*Plagiorchis javensis*	foodborne	yes	no	yes	504
Plasmodium	*Plasmodium falciparum*	vectorborne	yes	yes	no	505
Plasmodium	*Plasmodium malariae*	vectorborne	yes	yes	no	506
Plasmodium	*Plasmodium ovale*	vectorborne	yes	yes	no	506,507
Plasmodium	*Plasmodium simium*	vectorborne	yes	yes	yes	508
Plasmodium	*Plasmodium vivax*	vectorborne	yes	yes	no	509
Pneumocystis	*Pneumocystis carinii*	sapronosis	yes	yes	no	510
Polyomavirus	*JC virus*	unknown/unclassifiable	no	yes	no	511,512
Porphryomonas	*Porphryomonas catoniae*	opportunistic/normal flora	no	no	yes	513–515
Porphyromonas	*Porphyromonas levii*	unknown/unclassifiable	unknown	no	yes	516,517
Prevotella	*Prevotella melaninogenica*	opportunistic/normal flora	no	no	yes	518
Prevotella	*Prevotella tannerae*	opportunistic/normal flora	no	no	yes	514,519
Prion	*CJD agent*	unknown/unclassifiable	unknown	yes	yes	520,521
Propionibacterium	*Propionibacterium propionicus*	opportunistic/normal flora	no	no	yes	522,523
Proteus	*Proteus mirabilis*	opportunistic/normal flora	no	yes	no	524
Proteus	*Proteus morganii*	opportunistic/normal flora	no	yes	no	524,525
Pseudaallescheria	*Pseudaallescheria boydii*	sapronosis	yes	yes	no	526

Genus	*Pathogen*	Primary transmission category	Environmentally mediated?	WHO list?	Taylor list?	Refs
Pseudochaetosphaer	*Pseudochaetosphaeronema larense*	sapronosis	yes	yes	no	527
Pseudomonas	*Pseudomonas aeruginosa*	sapronosis	yes	yes	no	528
Pseudomonas	*Pseudomonas stutzeri*	foodborne	yes	no	yes	529
Pyramicocephalus	*Pyramicocephalus anthrocephalus*	foodborne	yes	no	yes	530
Pyrenochaeta	*Pyrenochaeta mackinnonii*	sapronosis	yes	yes	no	531,532
Pyrenochaeta	*Pyrenochaeta romeroi*	sapronosis	yes	yes	no	531,532
Pythium	*Pythium insidiosum*	sapronosis	yes	no	yes	139
Rhinocladiella	*Rhinocladiella aquaspersa*	sapronosis	yes	yes	no	533
Rhinocladiella	*Rhinocladiella atrovirens*	sapronosis	yes	yes	no	534
Rhinocladiella	*Rhinocladiella compacta*	sapronosis	yes	no	yes	535,536
Rhinosporidium	*Rhinosporidium seeberi*	sapronosis	yes	yes	no	537
Rhizomucor	*R miehei*	sapronosis	yes	yes	no	538,539
Rhizomucor	*Rhizomucor pusillus*	sapronosis	yes	yes	no	538–540
Rhizopus	*R azygosporus*	sapronosis	yes	yes	no	541
Rhizopus	*R microsporus*	sapronosis	yes	yes	no	542
Rhizopus	*R oryzae*	sapronosis	yes	yes	no	543,544
Rhizopus	*Rhizopus stolonifer*	sapronosis	yes	yes	yes	410,545,
Rhodococcus	*Rhodococcus fascians*	unknown/unclassifiable	yes	no	yes	
Rickettsia	*Rickettsia africae*	vectorborne	yes	no	yes	547,547,
Rickettsia	*Rickettsia akari*	vectorborne	yes	yes	no	549
Rickettsia	*Rickettsia conorii*	vectorborne	yes	no	yes	53,550,5
Rickettsia	*Rickettsia prowazekii*	vectorborne	yes	yes	no	552
Rickettsia	*Rickettsia rickettsii*	vectorborne	yes	yes	no	553
Rickettsia	*Rictularia species*	unknown/unclassifiable	unknown	no	yes	554–556
Roseolovirus	*Human herpesvirus 6*	human-to-human	no	yes	no	557
Roseolovirus	*Human herpesvirus 7*	human-to-human	no	yes	no	557
Rotavirus	*Rotavirus A*	waterborne	yes	yes	no	558–560
Rotavirus	*Rotavirus B*	waterborne	yes	yes	no	558–560
Rotavirus	*Rotavirus C*	human-to-human	no	yes	yes	558–560
Rotavirus	*Rotavirus D*	waterborne	yes	yes	no	558–560
Rotavirus	*Rotavirus E*	waterborne	yes	yes	no	558–560
Rotavirus	*Rotavirus F*	human-to-human	no	yes	yes	558–560
Rubivirus	*Rubella virus*	human-to-human	no	yes	no	561,562
Rubulavirus	*Menangle virus*	directly zoonotic (domestic)	yes	no	yes	563–565
Rubulavirus	*Mumps*	human-to-human	no	yes	no	566
Saccharomonospora	*Saccharomonospora viridis*	sapronosis	yes	no	yes	567
Saksenaea	*Saksenaea vasiformis*	sapronosis	yes	yes	no	568
Salmonella	*Salmonella enterica*	foodborne/waterborne	yes	yes	no	569
Salmonella	*Salmonella enteritidis*	sapronosis	yes	yes	no	425,570
Salmonella	*Salmonella typhi*	foodborne/waterborne	yes	yes	no	571
Salmonella	*Salmonella typhimurium*	sapronosis	yes	yes	no	572,573
Sarcocystis	*Sarcocystis hominis*	foodborne	yes	yes	no	574
Sarcocystis	*Sarcocystis lindemanni*	waterborne	yes	yes	no	574
Sarcocystis	*Sarcocystis subhominis*	foodborne	yes	yes	no	574
Schistosoma	*S bovis*	waterborne	yes	yes	no	7
Schistosoma	*S malayensis*	waterborne	yes	yes	no	7,575
Schistosoma	*S rodhaini*	waterborne	yes	yes	no	7
Schistosoma	*S spindale*	waterborne	yes	yes	no	7
Schistosoma	*Schistosoma haematobium*	waterborne	yes	yes	no	7
Schistosoma	*Schistosoma intercalatum*	waterborne	yes	yes	no	7,576
Schistosoma	*Schistosoma japonicum*	waterborne	yes	yes	no	7,577
Schistosoma	*Schistosoma mansoni*	waterborne	yes	yes	no	7
Schistosoma	*Schistosoma mattheei*	waterborne	yes	yes	yes	7,578
Schistosoma	*Schistosoma mekongi*	waterborne	yes	yes	no	579
Schistosoma	*Schistosoma spindale*	waterborne	yes	no	yes	580
Schistosomatium	*S douthitti*	waterborne	yes	yes	yes	7
Scolecobasidium	*Scolecobasidium humicola*	sapronosis	yes	no	yes	581
Scytalidium	*Scytalidium infestans*	unknown/unclassifiable	yes	no	yes	582
Selenomonas	*Selenomonas artemidis*	opportunistic/normal flora	no	no	yes	583
Selenomonas	*Selenomonas noxia*	opportunistic/normal flora	no	no	yes	584
Serratia	*Serratia rubidaea*	unknown/unclassifiable	yes	no	yes	585
Shigella	*Shigella boydii*	sapronosis	yes	yes	no	586,587
Shigella	*Shigella dysenteriae*	sapronosis	yes	yes	yes	586,587
Shigella	*Shigella flexneri*	sapronosis	yes	yes	no	586,587
Shigella	*Shigella sonnei*	sapronosis	yes	yes	no	586,587
Simplexvirus	*Human herpesvirus 1*	human-to-human	no	yes	no	588
Simplexvirus	*Human herpesvirus 2*	human-to-human	no	yes	yes	589
Spirillum	*Spirillum minus*	directly zoonotic (wildlife)	yes	yes	no	590,591
Sporothrix	*Sporothrix schenkii*	sapronosis	yes	yes	no	592
Staphylococcus	*Staphylococcus aureus*	human-to-human	no	yes	no	593

Genus	*Pathogen*	Primary transmission category	Environmentally mediated?	WHO list?	Taylor list?	Refs
Staphylococcus	*Staphylococcus warneri*	opportunistic/normal flora	no	no	yes	594
Stenotrophomonas	*Stenotrophomonas maltophilia*	nosocomial	yes	no	yes	595
Streptobacillus	*Streptobacillus moniliformis*	directly zoonotic (wildlife)	yes	yes	yes	596
Streptococcus	*Streptococcus agalactiae*	human-to-human	no	yes	yes	597
Streptococcus	*Streptococcus bovis*	unknown/unclassifiable	unknown	yes	no	598
Streptococcus	*Streptococcus constellatus*	opportunistic/normal flora	no	no	yes	599
Streptococcus	*Streptococcus equi*	directly zoonotic (domestic)	yes	yes	yes	600
Streptococcus	*Streptococcus gordonii*	opportunistic/normal flora	no	no	yes	601,602
Streptococcus	*Streptococcus pneumoniae*	human-to-human	no	yes	no	597
Streptococcus	*Streptococcus pyogenes*	human-to-human	no	yes	no	597
Streptomyces	*Streptomyces somaliensis*	sapronosis	yes	yes	no	603
Streptomyces	*Streptomyces sudanensis*	sapronosis	yes	yes	no	603,604
Strongyloides	*S fuelleborni*	soilborne	yes	yes	no	7
Strongyloides	*S papillosus*	soilborne	yes	yes	yes	7,605
Strongyloides	*S stercoralis*	soilborne	yes	yes	no	7
Strongyloides	*S westeri*	soilborne	yes	yes	no	7
Strongyloides	*Strongyloides ransomi*	soilborne	yes	yes	yes	7
Taenia	*T brauni (Multiceps brauni)*	foodborne	yes	yes	no	393
Taenia	*T crassiceps*	foodborne	yes	yes	no	7
Taenia	*T glomeratus (Multiceps glomeratus)*	foodborne	yes	yes	no	7
Taenia	*T longihamatus (Multiceps longihamatus)*	foodborne	yes	yes	no	7
Taenia	*T serialis*	foodborne	yes	yes	no	7
Taenia	*T taeniaeformis*	foodborne	yes	yes	no	7
Taenia	*Taenia saginata*	foodborne	yes	yes	no	7
Taenia	*Taenia solium*	foodborne	yes	yes	no	7
Taeniolella	*Taeniolella exilis*	unknown/unclassifiable	yes	no	yes	606
Tatumella	*Tatumella ptyseos*	unknown/unclassifiable	no	no	yes	607
Ternidens	*Ternidens deminutus*	unknown/unclassifiable	yes	yes	no	7
Tetraploa	*Tetraploa aristata*	unknown/unclassifiable	yes	no	yes	608
Thelazia	*Thelazia californiensis*	vectorborne	yes	yes	no	7
Thelazia	*Thelazia callipaeda*	vectorborne	yes	yes	no	7
Thogotovirus	*Batken virus*	unknown/unclassifiable	unknown	no	yes	609,610
Toxocara	*T canis*	soilborne	yes	yes	yes	7
Toxocara	*Toxocara cati*	soilborne	yes	yes	yes	611
Toxoplasma	*Toxoplasma gondii*	foodborne	yes	yes	no	612
Trachipleistophora	*Trachipleistophora hominis*	sapronosis	yes	yes	no	233,445
Treponema	*Treponema carateum*	human-to-human	no	yes	no	613
Treponema	*Treponema pallidum*	human-to-human	no	yes	no	614
Trichinella	*T nativa*	foodborne	yes	yes	no	7
Trichinella	*T pseudospiralis*	foodborne	yes	yes	no	7
Trichinella	*T spiralis*	foodborne	yes	yes	no	7
Trichinella	*T T5*	foodborne	yes	yes	no	615
Trichinella	*Trichinella britovi*	foodborne	yes	yes	no	7
Trichinella	*Trichinella nelsoni*	foodborne	yes	yes	no	7
Trichoderma	*Trichoderma viride*	sapronosis	yes	no	yes	616
Trichomonas	*Trichomonas vaginalis*	human-to-human	no	yes	yes	617
Trichophyton	*T ajelloi*	sapronosis	yes	yes	no	249,618,
Trichophyton	*T bullosum*	directly zoonotic (domestic)	yes	yes	no	249,618,
Trichophyton	*T concentricum*	human-to-human	no	yes	no	249,618,
Trichophyton	*T equinum*	directly zoonotic (domestic)	yes	yes	no	249,618,
Trichophyton	*T gourvilii*	human-to-human	no	yes	no	249,618,
Trichophyton	*T megninii*	human-to-human	no	yes	no	249,618,
Trichophyton	*T mentagrophytes*	directly zoonotic (wildlife)	yes	yes	no	249,618,
Trichophyton	*T rubrum*	human-to-human	no	yes	no	249,618,
Trichophyton	*T schoenleinii*	human-to-human	no	yes	no	249,618,
Trichophyton	*T soudanense*	human-to-human	no	yes	no	618–620
Trichophyton	*T tonsurans*	human-to-human	no	yes	no	249,618,
Trichophyton	*T verrucosum*	directly zoonotic (domestic)	yes	yes	no	249,618,
Trichophyton	*T violaceum*	human-to-human	no	yes	no	618–620
Trichophyton	*Trichophyton simii*	directly zoonotic (wildlife)	yes	yes	yes	621–623
Trichosporon	*Trichosporon asahii*	sapronosis	yes	yes	yes	624–626
Trichosporon	*Trichosporon beigelii*	sapronosis	yes	yes	no	627
Trichosporon	*Trichosporon cutaneum*	sapronosis	yes	no	yes	628
Trichostrongylus	*T affinis*	foodborne	yes	yes	no	7,629
Trichostrongylus	*T axei*	foodborne	yes	yes	no	7,629
Trichostrongylus	*T brevis*	unknown/unclassifiable	yes	yes	yes	630,6317
Trichostrongylus	*T calcaratus*	foodborne	yes	yes	no	7,629
Trichostrongylus	*T capricola*	foodborne	yes	yes	no	7,629
Trichostrongylus	*T colubriformis*	foodborne	yes	yes	no	7,629
Trichostrongylus	*T instabilis*	foodborne	yes	yes	no	7,629

Genus	*Pathogen*	Primary transmission category	Environmentally mediated?	WHO list?	Taylor list?	Refs
Trichostrongylus	*T lerouxi*	foodborne	yes	yes	no	[7,629]
Trichostrongylus	*T probulurus*	foodborne	yes	yes	no	[7,629]
Trichostrongylus	*T skrjabini*	foodborne	yes	yes	no	[7,629]
Trichostrongylus	*T vitrinus*	foodborne	yes	yes	no	[7,629]
Trichostrongylus	*Trichostrongylus orientalis*	foodborne	yes	yes	yes	[7,632,63]
Trichuris	*Trichuris trichiura*	soilborne	yes	yes	no	[55,634]
Tritirachium	*Tritirachium oryzae*	sapronosis	yes	no	yes	[635]
Trypanosoma	*Trypanosoma brucei*	vectorborne	yes	yes	no	[636]
Trypanosoma	*Trypanosoma cruzi*	vectorborne	yes	yes	no	[637,638]
Tsukamurella	*Tsukamurella paurometabola*	sapronosis	yes	no	yes	[639]
Varicella	*Human herpesvirus 3*	human-to-human	no	yes	no	[640]
Vesiculovirus	*Piry virus*	vectorborne	yes	yes	no	[641]
Vesiculovirus	*Vesicular stomatitis virus*	vectorborne	yes	yes	no	[642]
Vibrio	*Vibrio cholerae*	sapronosis	yes	yes	no	[643]
Vibrio	*Vibrio cincinnatiensis*	sapronosis	yes	no	yes	[644]
Vibrio	*Vibrio parahaemolyticus*	foodborne	yes	yes	no	[645]
Vibrio	*Vibrio vulnificus*	foodborne	yes	yes	no	[646]
Vittaforma	*Vittaforma corneae*	sapronosis	yes	yes	no	[7,233,44]
Volutella	*Volutella cinerescens*	unknown/unclassifiable	yes	no	yes	[647]
Watsonius	*Watsonius watsoni*	foodborne	yes	yes	no	[7]
Wuchereria	*Wuchereria bancrofti*	vectorborne	yes	yes	no	[123,648]
Yatapoxvirus	*Tanapox virus*	human-to-human	unknown	yes	yes	[649]
Yatapoxvirus	*Yaba monkey tumor virus*	directly zoonotic (wildlife)	yes	yes	no	[650]
Yersinia	*Yersinia enterocolitica*	sapronosis	yes	yes	no	[651]
Yersinia	*Yersinia frederiksenii*	sapronosis	yes	no	yes	[652]
Yersinia	*Yersinia intermedia*	unknown/unclassifiable	yes	no	yes	[653]
Yersinia	*Yersinia pestis*	vectorborne	yes	yes	no	[654]
Yersinia	*Yersinia ruckeri*	unknown/unclassifiable	yes	no	yes	[655]

References for this table:

1 Senn L, Entenza JM, Greub G, *et al.* Bloodstream and endovascular infections due to Abiotrophia defectiva and Granulicatellaspecies. *BMC Infectious Diseases* 2006; **6**. DOI:10.1186/1471-2334-6-9.

2 Bouvet A, Grimont F, Grimont PAD. Streptococcus defectivus sp. nov. and Streptococcus adjacens sp. nov., Nutritionally Variant Streptococci from Human Clinical Specimens. *International Journal of Systematic Bacteriology* 1989; **39**: 290–4.

3 Woo PC, Leung S-Y, Ngan AH, Lau SK, Yuen K-Y. A significant number of reported *Absidia corymbifera* (*Lichtheimia corymbifera*) infections are caused by *Lichtheimia ramosa* (syn. *Lichtheimia hongkongensis*): an emerging cause of mucormycosis. *Emerging Microbes & Infections* 2012; **1**: 1–8.

4 Ahmed Khan N. Pathogenesis of Acanthamoeba infections. *Microbial Pathogenesis* 2003; **34**: 277–85.

5 Visvesvara GS, Stehr-Green JK. Epidemiology of Free-Living Ameba Infections [1]. *The Journal of Protozoology* 1990; **37**: 25s–33s.

6 Visvesvara GS, Moura H, Schuster FL. Pathogenic and opportunistic free-living amoebae: *Acanthamoeba* spp., *Balamuthia mandrillaris* , *Naegleria fowleri* , and *Sappinia diploidea*. *FEMS Immunology & Medical Microbiology* 2007; **50**: 1–26.

7 Ashford RW, Crewe W. The parasites of Homo sapiens: an annotated checklist of the protozoa, helminths and arthropods for which we are home, 2 ed. London: Taylor & Francis, 2003.

8 Kiredjian M, Holmes B, Kersters K, Guilvout I, De Ley J. Alcaligenes piechaudii, a New Species from Human Clinical Specimens and the Environment. *International Journal of Systematic Bacteriology* 1986; **36**: 282–7.

9 Seifert H, Baginski R, Schulze A, Pulverer G. Antimicrobial susceptibility of Acinetobacter species. *Antimicrobial Agents and Chemotherapy* 1993; **37**: 750–3.

10 Linde H-J, Hahn J, Holler E, Reischl U, Lehn N. Septicemia Due to Acinetobacter junii. *Journal of Clinical Microbiology* 2002; **40**: 2696–7.

11 Bergogne-Bérézin E, Towner KJ. Acinetobacter spp. as nosocomial pathogens: microbiological, clinical, and epidemiological features. *Clin Microbiol Rev* 1996; **9**: 148–65.

12 Das S, Saha R, Dar SA, Ramachandran VG. Acr emonium Species: A Review of the Etiological Agents of Emerging Hyalohyphomycosis. *Mycopathologia* 2010; **170**: 361–75.

13 Gupta A, Srinivasan R, Kaliaperumal S, Saha I. Post-traumatic fungal endophthalmitis—a prospective study. *Eye* 2008; **22**: 13–7.

14 Yalaz M. Fatal disseminated Acremonium strictum infection in a preterm newborn: a very rare cause of neonatal septicaemia. *Journal of Medical Microbiology* 2003; **52**: 835–7.

15 Walsh TJ, Groll AH. Emerging fungal pathogens: evolving challenges to immunocompromised patients for the twenty-first century. *Transplant Infectious Disease* 1999; **1**: 247–61.

16 Matthews S, Dart A, Dowling B, Hodgson J, Hodgson D. Peritonitis associated with Actinobacillus equuli in horses: 51 cases. *Australian Veterinary Journal* 2001; **79**: 536–9.

17 Ashhurst-Smith C, Norton R, Thoreau W, Peel MM. Actinobacillus equuli septicemia: an unusual zoonotic infection. *J Clin Microbiol* 1998; **36**: 2789–90.

18 Trujillo ME, Goodfellow M. *Actinomadura*. In: Whitman WB, Rainey F, Kämpfer P, *et al.*, eds. Bergey's Manual of Systematics of Archaea and Bacteria. Chichester, UK: John Wiley & Sons, Ltd, 2015: 1–32.

19 Valour F, Ferry T, Karsenty J, *et al.* Actinomycosis: etiology, clinical features, diagnosis, treatment, and management. *Infection and Drug Resistance* 2014; : 183.

20 McVey DS, Kennedy M, Chengappa MM, editors. Veterinary microbiology, 3rd ed. Ames, Iowa: Wiley-Blackwell, 2013.

21 Apotheloz C, Regamey C. Disseminated Infection Due to Actinomyces meyeri: Case Report and Review. *Clinical Infectious Diseases* 1996; **22**: 621–5.

22 Shoop WL, Corkum KC. Epidemiology of Alaria marcianae mesocercariae in Louisiana. *J Parasitol* 1981; **67**: 928–31.

23 Ijaz MK, Brunner AH, Sattar SA, Nair RC, Johnson-Lussenburg CM. Survival Characteristics of Airborne Human Coronavirus 229E. *Journal of General Virology* 1985; **66**: 2743–8.

24 Tellier R. Review of Aerosol Transmission of Influenza A Virus. *Emerg Infect Dis* 2006; **12**: 1657–62.

25 Vega-Rúa A, Lourenço-de-Oliveira R, Mousson L, *et al.* Chikungunya Virus Transmission Potential by Local Aedes Mosquitoes in the Americas and Europe. *PLoS Negl Trop Dis* 2015; **9**: e0003780.

26 Nickerson JP, Kannabiran S, Burbank HN. MRI findings in eastern equine encephalitis: the "parenthesis" sign. *Clinical Imaging* 2016; **40**: 222–3.

27 Reisler RB, Gibbs PH, Danner DK, Boudreau EF. Immune interference in the setting of same-day administration of two similar inactivated alphavirus vaccines: Eastern equine and western equine encephalitis. *Vaccine* 2012; **30**: 7271–7.

28 Hoyer IJ, Acevedo C, Wiggins K, Alto BW, Burkett-Cadena ND. Patterns of Abundance, Host Use, and Everglades Virus Infection in *Culex* (*Melanoconion*) *cedecei* Mosquitoes, Florida, USA. *Emerg Infect Dis* 2019; **25**: 1093–100.

29 Lanciotti RS, Ludwig ML, Rwaguma EB, *et al.* Emergence of Epidemic O'nyong-nyong Fever in Uganda after a 35-Year Absence: Genetic Characterization of the Virus. *Virology* 1998; **252**: 258–68.

30 Turell MJ, LundstrÖM JO, Niklasson B. Transmission of Ockelbo Virus by Aedes cinereus, Ae, communis, and Ae. excrucians (Diptera: Culicidae) Collected in an Enzootic Area in Central Sweden. *Journal of Medical Entomology* 1990; **27**: 266–8.

31 Harley D, Sleigh A, Ritchie S. Ross River Virus Transmission, Infection, and Disease: a Cross-Disciplinary Review. *Clinical Microbiology Reviews* 2001; **14**: 909–32.

32 Weaver SC, Ferro C, Barrera R, Boshell J, Navarro J-C. V ENEZUELAN E QUINE E NCEPHALITIS. *Annu Rev Entomol* 2004; **49**: 141–74.

33 Allison AB, Stallknecht DE, Holmes EC. Evolutionary genetics and vector adaptation of recombinant viruses of the western equine encephalitis antigenic complex provides new insights into alphavirus diversity and host switching. *Virology* 2015; **474**: 154–62.

34 Romano C, Fimiani M, Pellegrino M, *et al.* Cutaneous phaeohyphomycosis due to *Alternaria tenuissima*. *Mycoses* 1996; **39**: 211–5.

35 Robertshaw H, Higgins E. Cutaneous infection with Alternaria tenuissima in an immunocompromised patient. *Br J Dermatol* 2005; **153**: 1047–9.

36 Yoshida Y. Comparative studies on Ancylostoma braziliense and Ancylostoma ceylanicum. I. The adult stage. *J Parasitol* 1971; **57**: 983–9.

37 Prociv P, Croese J. Human enteric infection with Ancylostoma caninum : hookworms reappraised in the light of a "new" zoonosis. *Acta Tropica* 1996; **62**: 23–44.

38 Traub RJ. Ancylostoma ceylanicum, a re-emerging but neglected parasitic zoonosis. *International Journal for Parasitology* 2013; **43**: 1009–15.

39 Brooker S, Clements ACA, Bundy DAP. Global Epidemiology, Ecology and Control of Soil-Transmitted Helminth Infections. In: Advances in Parasitology. Elsevier, 2006: 221–61.

40 Hotez PJ, Brooker S, Bethony JM, Bottazzi ME, Loukas A, Xiao S. Hookworm Infection. *N Engl J Med* 2004; **351**: 799–807.

41 Pien FD, Pien BC. Angiostrongylus cantonensis eosinophilic meningitis. *International Journal of Infectious Diseases* 1999; **3**: 161–3.

42 Slom TJ, Cortese MM, Gerber SI, *et al.* An Outbreak of Eosinophilic Meningitis Caused by *Angiostrongylus cantonensis* in Travelers Returning from the Caribbean. *N Engl J Med* 2002; **346**: 668–75.

43 Ellis D, Women's and Children's Hospital (Adelaide SA), Mycology Unit. Descriptions of medical fungi. North Adelaide, S. Aust.: The authors, 2007.

44 Cano J, Guarro J. The genus Aphanoascus. *Mycological Research* 1990; **94**: 355–77.

45 Chakrabarti A, Ghosh A, Prasad GS, *et al.* Apophysomyces elegans: an Emerging Zygomycete in India. *J Clin Microbiol* 2003; **41**: 783–8.

46 Levy CE, Pedro RJ, Von Nowakonski A, Holanda LM, Brocchi M, Ramos MC. *Arcanobacterium pyogenes* Sepsis in Farmer, Brazil. *Emerg Infect Dis* 2009; **15**: 1131–2.

47 Madsen M, Høi Sørensen G, Aalbaek B, Hansen JW, Bjørn H. Summer mastitis in heifers: studies on the seasonal occurrence of Actinomyces pyogenes, Peptostreptococcus indolicus and Bacteroidaceae in clinically healthy cattle in Denmark. *Vet Microbiol* 1992; **30**: 243–55.

48 Grant A, Seregin A, Huang C, *et al.* Junín virus pathogenesis and virus replication. *Viruses* 2012; **4**: 2317–39.

49 Enria DA, Briggiler AM, Feuillade MR. An overview of the epidemiological, ecological and preventive hallmarks of Argentine haemorrhagic fever (Junin virus). *Bulletin de l'Institut Pasteur* 1998; **96**: 103–14.

50 McCormick JB, Fisher-Hoch SP. Lassa fever. *Curr Top Microbiol Immunol* 2002; **262**: 75–109.

51 Kuns ML. Epidemiology of Machupo Virus Infection. *The American Journal of Tropical Medicine and Hygiene* 1965; **14**: 813–6.

52 Chai J-Y, Shin E-H, Lee S-H, Rim H-J. Foodborne Intestinal Flukes in Southeast Asia. *Korean J Parasitol* 2009; **47**: S69.

53 Walker M, Hall A, Basáñez M-G. Ascaris lumbricoides. In: Ascaris: The Neglected Parasite. Elsevier, 2013: 155–201.

54 Brooker SJ, Pullan RL. Ascaris lumbricoides and Ascariasis. In: Ascaris: The Neglected Parasite. Elsevier, 2013: 343–62.

55 Bethony J, Brooker S, Albonico M, *et al.* Soil-transmitted helminth infections: ascariasis, trichuriasis, and hookworm. *The Lancet* 2006; **367**: 1521–32.

56 Linares G, McGarry PA, Baker RD. Solid solitary aspergillotic granuloma of the brain: Report of a case due to Aspergillus candidus and review of the literature. *Neurology* 1971; **21**: 177–177.

57 Coriglione G, Stella G, Gafa L, *et al.* Neosartorya fischeri var fischeri (Wehmer) Malloch and Cain 1972 (Anamorph: Aspergillus fischerianus Samson and Gams 1985) as a cause of mycotic keratitis. *Eur J Epidemiol* 1990; **6**: 382–5.

58 Roselle GA, Baird IM. Aspergillus flavipes group osteomyelitis. *Arch Intern Med* 1979; **139**: 590–2.

59 Hedayati MT, Pasqualotto AC, Warn PA, Bowyer P, Denning DW. Aspergillus flavus: human pathogen, allergen and mycotoxin producer. *Microbiology* 2007; **153**: 1677–92.

60 Latgé JP. Aspergillus fumigatus and aspergillosis. *Clin Microbiol Rev* 1999; **12**: 310–50.

61 Hodgson MJ, Morey P, Leung W-Y, *et al.* Building-Associated Pulmonary Disease From Exposure toStachybotrys chartarum and Aspergillus versicolor: *Journal of Occupational & Environmental Medicine* 1998; **40**: 241–9.

62 Segal BH, DeCarlo ES, Kwon-Chung KJ, Malech HL, Gallin JI, Holland SM. Aspergillus nidulansInfection in Chronic Granulomatous Disease. *Medicine* 1998; **77**: 345–54.

63 Schuster E, Dunn-Coleman N, Frisvad JC, Van Dijck PWM. On the safety of Aspergillus niger--a review. *Appl Microbiol Biotechnol* 2002; **59**: 426–35.

64 Steinbach WJ, Benjamin DK, Kontoyiannis DP, *et al.* Infections due to Aspergillus terreus: a multicenter retrospective analysis of 83 cases. *Clin Infect Dis* 2004; **39**: 192–8.

65 Warris A, Bjørneklett A, Gaustad P. Invasive pulmonary aspergillosis associated with infliximab therapy. *N Engl J Med* 2001; **344**: 1099–100.

66 Halsey C, Lumley H, Luckit J. Necrotising external otitis caused by Aspergillus wentii: a case report: Necrotising external otitis caused by A. wentii. *Mycoses* 2011; **54**: e211–3.

67 Blyth W, Grant IWB, Blackadder ES, Greenberg M. Fungal antigens as a source of sensitization and respiratory disease in Scottish maltworkers. *Clinical & Experimental Allergy* 1977; **7**: 549–62.

68 Blyth W. The occurrence and nature of alveolitis-inducing substances in Aspergillus clavatus. *Clin Exp Immunol* 1978; **32**: 272–82.

69 Cooper RC, Olivieri AW, Danielson RE, Badger PG. Evaluation of military field-water quality: Volume 6, Infectious organisms of military concern associated with nonconsumptive exposure: Assessment of health risks and recommendations for establishing related standards. 1986.

70 Alexander DJ. Newcastle disease and other avian paramyxoviruses. *Rev - Off Int Epizoot* 2000; **19**: 443–62.

71 Homer MJ, Aguilar-Delfin I, Telford SR, Krause PJ, Persing DH. Babesiosis. *Clin Microbiol Rev* 2000; **13**: 451–69.

72 Vannier E, Gewurz B, Krause P. Human Babesiosis. *Elsevier* 2008. https://www-clinicalkey-com.stanford.idm.oclc.org/#!/content/playContent/1-s2.0-S0891552008000251?returnurl=null&referrer=null (accessed March 14, 2020).

73 Turnbull PC. Definitive identification of Bacillus anthracis--a review. *J Appl Microbiol* 1999; **87**: 237–40.

74 Ganz HH, Turner WC, Brodie EL, *et al.* Interactions between Bacillus anthracis and Plants May Promote Anthrax Transmission. *PLOS Neglected Tropical Diseases* 2014; **8**: e2903.

75 Ehling-Schulz M, Fricker M, Scherer S. Bacillus cereus, the causative agent of an emetic type of food-borne illness. *Mol Nutr Food Res* 2004; **48**: 479–87.

76 Krawczyk AO, de Jong A, Holsappel S, *et al.* Genome Sequences of 12 Spore-Forming Bacillus Species, Comprising Bacillus coagulans, Bacillus licheniformis, Bacillus amyloliquefaciens, Bacillus sporothermodurans, and Bacillus vallismortis, Isolated from Foods. *Genome Announc* 2016; **4**. DOI:10.1128/genomeA.00103-16.

77 Wenzler E, Kamboj K, Balada-Llasat J-M. Severe Sepsis Secondary to Persistent Lysinibacillus sphaericus, Lysinibacillus fusiformis and Paenibacillus amylolyticus Bacteremia. *International Journal of Infectious Diseases* 2015; **35**: 93–5.

78 Kellen WR, Clark TB, Lindegren JE, Ho BC, Rogoff MH, Singer S. Bacillus sphaericus Neide as a pathogen of mosquitoes. *Journal of Invertebrate Pathology* 1965; **7**: 442–8.

79 Hertlein BC, Levy R, Miller TW. Recycling potential and selective retrieval of Bacillus sphaericus from soil in a mosquito habitat. *Journal of Invertebrate Pathology* 1979; **33**: 217–21.

80 Waterfield NR, Wren BW, ffrench-Constant RH. Invertebrates as a source of emerging human pathogens. *Nature Reviews Microbiology* 2004; **2**: 833–41.

81 Sack RB, Myers LL, Almeido - Hill J, *et al.* Enterotoxigenic Bacteroides Fragilis: Epidemiologic Studies of its Role as a Human Diarrhoeal Pathogen. *Journal of Diarrhoeal Diseases Research* 1992; **10**: 4–9.

82 Xu J, Bjursell MK, Himrod J, *et al.* A Genomic View of the Human-Bacteroides thetaiotaomicron Symbiosis. *Science* 2003; **299**: 2074–6.

83 Curtis MM, Hu Z, Klimko C, Narayanan S, Deberardinis R, Sperandio V. The gut commensal Bacteroides thetaiotaomicron exacerbates enteric infection through modification of the metabolic landscape. *Cell Host Microbe* 2014; **16**: 759–69.

84 Wexler HM. Bacteroides: the Good, the Bad, and the Nitty-Gritty. *CMR* 2007; **20**: 593–621.

85 Arean VM, Koppisch E. Balantidiasis; a review and report of cases. *Am J Pathol* 1956; **32**: 1089–115.

86 Schuster FL, Ramirez-Avila L. Current world status of Balantidium coli. *Clin Microbiol Rev* 2008; **21**: 626–38.

87 Brenner DJ, O'connor SP, Winkler HH, Steigerwalt AG. Proposals to unify the genera Bartonella and Rochalimaea, with descriptions of Bartonella quintana comb. nov., Bartonella vinsonii comb. nov., Bartonella henselae comb. nov., and Bartonella elizabethae comb. nov., and to remove the family Bartonellaceae from the order Rickettsiales. *International journal of systematic bacteriology* 1993. DOI:10.1099/00207713-43-4-777.

88 Ihler GM. Bartonella bacilliformis: dangerous pathogen slowly emerging from deep background. *FEMS Microbiol Lett* 1996; **144**: 1–11.

89 Chomel BB, Boulouis H-J, Maruyama S, Breitschwerdt EB. Bartonella Spp. in Pets and Effect on Human Health. *Emerg Infect Dis* 2006; **12**: 389–94.

90 Ohl ME, Spach DH. Bartonella quintana and urban trench fever. *Clin Infect Dis* 2000; **31**: 131–5.

91 Breitschwerdt EB. Bartonellosis: One Health Perspectives for an Emerging Infectious Disease. *ILAR J* 2014; **55**: 46–58.

92 Gugnani HC. A review of zygomycosis due to Basidiobolus ranarum. *Eur J Epidemiol* 1999; **15**: 923–9.

93 Sorvillo F, Ash LR, Berlin OGW, Yatabe J, Degiorgio C, Morse SA. Baylisascaris procyonis: An Emerging Helminthic Zoonosis. *Emerg Infect Dis* 2002; **8**: 355–9.

94 Rehner SA, Minnis AM, Sung G-H, Luangsa-ard JJ, Devotto L, Humber RA. Phylogeny and systematics of the anamorphic, entomopathogenic genus Beauveria. *Mycologia* 2011; **103**: 1055–73.

95 Gaunt ER, Hardie A, Claas ECJ, Simmonds P, Templeton KE. Epidemiology and clinical presentations of the four human coronaviruses 229E, HKU1, NL63, and OC43 detected over 3 years using a novel multiplex real-time PCR method. *J Clin Microbiol* 2010; **48**: 2940–7.

96 Flanagan KL, Bryceson ADM. Disseminated Infection Due to Bipolaris australiensis in a Young Immunocompetent Man: Case Report and Review. *Clin Infect Dis* 1997; **25**: 311–3.

97 Walsh TJ, Groll AH. Emerging fungal pathogens: evolving challenges to immunocompromised patients for the twenty-first century. *Transplant Infectious Disease* 1999; **1**: 247–61.

98 Chowdhary A, Randhawa HS, Singh V, *et al.* Bipolaris hawaiiensis as etiologic agent of allergic bronchopulmonary mycosis: first case in a paediatric patient. *Med Mycol* 2011; **49**: 760–5.

99 Chapman SW, Dismukes WE, Proia LA, *et al.* Clinical Practice Guidelines for the Management of Blastomycosis: 2008 Update by the Infectious Diseases Society of America. *Clinical Infectious Diseases* 2008; **46**: 1801–12.

100 Sarosi GA, Davies SF. Blastomycosis. *Am Rev Respir Dis* 1979; **120**: 911–38.

101 Galeziok M, Roberts I, Passalacqua J-A. Bordetella bronchiseptica pneumonia in a man with acquired immunodeficiency syndrome: a case report. *J Med Case Reports* 2009; **3**: 76.

102 Goodnow RA. Biology of Bordetella bronchiseptica. *Microbiol Rev* 1980; **44**: 722–38.

103 Porter JF, Parton R, Wardlaw AC. Growth and survival of Bordetella bronchiseptica in natural waters and in buffered saline without added nutrients. *Appl Environ Microbiol* 1991; **57**: 1202–6.

104 Khelef N, Danve B, Quentin-Millet MJ, Guiso N. Bordetella pertussis and Bordetella parapertussis: two immunologically distinct species. *Infect Immun* 1993; **61**: 486–90.

105 Heininger U, Stehr K, Schmitt-Grohé S, *et al.* Clinical characteristics of illness caused by Bordetella parapertussis compared with illness caused by Bordetella pertussis. *Pediatr Infect Dis J* 1994; **13**: 306–9.

106 Parkhill J, Sebaihia M, Preston A, *et al.* Comparative analysis of the genome sequences of Bordetella pertussis , Bordetella parapertussis and Bordetella bronchiseptica. *Nature Genetics* 2003; **35**: 32–40.

107 Danthis M. Whooping cough. *Nurs Stand* 2014; **28**: 53.

108 Trainor EA, Nicholson TL, Merkel TJ. Bordetella pertussis transmission. *Pathog Dis* 2015; **73**: ftv068.

109 Rott R, Herzog S, Fleischer B, *et al.* Detection of serum antibodies to Borna disease virus in patients with psychiatric disorders. *Science* 1985; **228**: 755–6.

110 Staeheli P, Sauder C, Hausmann J, Ehrensperger F, Schwemmle M. Epidemiology of Borna disease virus. *Journal of General Virology,* 2000; **81**: 2123–35.

111 Johnson RC, Schmid GP, Hyde FW, Steigerwalt AG, Brenner DJ. Borrelia burgdorferi sp. nov.: Etiologic Agent of Lyme Disease. *International Journal of Systematic Bacteriology* 1984; **34**: 496–7.

112 Fraser CM, Casjens S, Huang WM, *et al.* Genomic sequence of a Lyme disease spirochaete, Borrelia burgdorferi. *Nature* 1997; **390**: 580–6.

113 Schwan TG, Raffel SJ, Schrumpf ME, Porcella SF. Diversity and Distribution of Borrelia hermsii. *Emerg Infect Dis* 2007; **13**: 436–42.

114 Assous MV, Wilamowski A. Relapsing fever borreliosis in Eurasia—forgotten, but certainly not gone! *Clinical Microbiology and Infection* 2009; **15**: 407–14.

115 Parola P, Ryelandt J, Mangold AJ, Mediannikov O, Guglielmone AA, Raoult D. Relapsing fever Borrelia in Ornithodoros ticks from Bolivia. *Ann Trop Med Parasitol* 2011; **105**: 407–11.

116 Cutler SJ, Moss J, Fukunaga M, Wright DJ, Fekade D, Warrell D. Borrelia recurrentis characterization and comparison with relapsing-fever, Lyme-associated, and other Borrelia spp. *Int J Syst Bacteriol* 1997; **47**: 958–68.

117 Lyons DC. The Incidence and Significance of the Presence of Borrelia vincenti and Other Spirochaetaceae on Beverage Glasses. *J Bacteriol* 1936; **31**: 523–6.

118 Rebaudet S, Parola P. Epidemiology of relapsing fever borreliosis in Europe. *FEMS Immunol Med Microbiol* 2006; **48**: 11–5.

119 Benoldi D, Alinovi A, Polonelli L, *et al.* Botryomyces caespitosus as an agent of cutaneous phaeohyphomycosis. *J Med Vet Mycol* 1991; **29**: 9–13.

120 Godfroid J, Cloeckaert A, Liautard J-P, *et al.* From the discovery of the Malta fever's agent to the discovery of a marine mammal reservoir, brucellosis has continuously been a re-emerging zoonosis. *Vet Res* 2005; **36**: 313–26.

121 Roop RM, Gaines JM, Anderson ES, Caswell CC, Martin DW. Survival of the fittest: how Brucella strains adapt to their intracellular niche in the host. *Med Microbiol Immunol* 2009; **198**. DOI:10.1007/s00430-009-0123-8.

122 Fischer P, Supali T, Maizels RM. Lymphatic filariasis and Brugia timori: prospects for elimination. *Trends Parasitol* 2004; **20**: 351–5.

123 Ottesen EA, Duke BO, Karam M, Behbehani K. Strategies and tools for the control/elimination of lymphatic filariasis. *Bull World Health Organ* 1997; **75**: 491–503.

124 Jex AR, Waeschenbach A, Hu M, *et al.* The mitochondrial genomes of Ancylostoma caninum and Bunostomum phlebotomum – two hookworms of animal health and zoonotic importance. *BMC Genomics* 2009; **10**: 79.

125 Beaver PC. Larva migrans. *Experimental Parasitology* 1956; **5**: 587–621.

126 Smithburn KC, Paterson HE, Kokernot RH, de Meillon B. Isolation of Bunyamwera Virus from a Naturally Infected Human Being and Further Isolations from Aedes (Banksinella) Circumluteolus Theo. 1. *The American Journal of Tropical Medicine and Hygiene* 1958; **7**: 579–84.

127 Silva EB, Dow SW. Development of Burkholderia mallei and pseudomallei vaccines. *Front Cell Infect Microbiol* 2013; **3**: 10.

128 Inglis TJJ, Sagripanti J-L. Environmental factors that affect the survival and persistence of Burkholderia pseudomallei. *Appl Environ Microbiol* 2006; **72**: 6865–75.

129 Wilson R, Anderson LJ, Holman RC, Gary GW, Greenberg HB. Waterborne Gastroenteritis due to the Norwalk Agent: Clinical and Epidemiologic Investigation. *Am J Public Health* 1982; **72**: 72–4.

130 Kohn MA, Farley TA, Ando T, *et al.* An Outbreak of Norwalk Virus Gastroenteritis Associated With Eating Raw Oysters: Implications for Maintaining Safe Oyster Beds. *JAMA* 1995; **273**: 466–71.

131 Kapperud G, Skjerve E, Bean NH, Ostroff SM, Lassen J. Risk factors for sporadic Campylobacter infections: results of a case-control study in southeastern Norway. *Journal of Clinical Microbiology* 1992; **30**: 3117–21.

132 Friedman CR, Hoekstra RM, Samuel M, *et al.* Risk factors for sporadic Campylobacter infection in the United States: A case-control study in FoodNet sites. *Clin Infect Dis* 2004; **38 Suppl 3**: S285-296.

133 Pfaller MA. Nosocomial candidiasis: emerging species, reservoirs, and modes of transmission. *Clin Infect Dis* 1996; **22 Suppl 2**: S89-94.

134 Calderone RA, Fonzi WA. Virulence factors of Candida albicans. *Trends Microbiol* 2001; **9**: 327–35.

135 Singh R, Parija SC. Candida parapsilosis : an emerging fungal pathogen. *Indian Journal of Medical Research* 2012; **136**: 671.

136 SAMARANAYAKE YH, SAMARANAYAKE LP. Candida krusei: biology, epidemiology, pathogenicity and clinical manifestations of an emerging pathogen. *Journal of Medical Microbiology,* 1994; **41**: 295–310.

137 Wingard JR, Merz WG, Rinaldi MG, Johnson TR, Karp JE, Saral R. Increase in Candida krusei Infection among Patients with Bone Marrow Transplantation and Neutropenia Treated Prophylactically with Fluconazole. *New England Journal of Medicine* 1991; **325**: 1274–7.

138 Merz WG, Karp JE, Schron D, Saral R. Increased incidence of fungemia caused by Candida krusei. *Journal of Clinical Microbiology* 1986; **24**: 581–4.

139 Gaastra W, Lipman LJA. Capnocytophaga canimorsus. *Vet Microbiol* 2010; **140**: 339–46.

140 Brenner DJ, Hollis DG, Fanning GR, Weaver RE. Capnocytophaga canimorsus sp. nov. (formerly CDC group DF-2), a cause of septicemia following dog bite, and C. cynodegmi sp. nov., a cause of localized wound infection following dog bite. *J Clin Microbiol* 1989; **27**: 231–5.

141 Parenti DM, Snydman DR. Capnocytophaga Species: Infections in Nonimmunocompromised and Immunocompromised Hosts. *J Infect Dis* 1985; **151**: 140–7.

142 Wormser GP, Bottone EJ. Cardiobacterium hominis: review of microbiologic and clinical features. *Rev Infect Dis* 1983; **5**: 680–91.

143 Malani AN, Aronoff DM, Bradley SF, Kauffman CA. Cardiobacterium hominis endocarditis: Two cases and a review of the literature. *Eur J Clin Microbiol Infect Dis* 2006; **25**: 587–95.

144 Slotnick IJ, Dougherty M. FURTHER CHARACTERIZATION OF AN UNCLASSIFIED GROUP OF BACTERIA CAUSING ENDOCARDITIS IN MAN: CARDIOBACTERIUM HOMINIS GEN. ET SP. N. *Antonie Van Leeuwenhoek* 1964; **30**: 261–72.

145 Rams TE, Hawley CE, Whitaker EJ, Degener JE, van Winkelhoff AJ. Centipeda periodontii in human periodontitis. *Odontology* 2015; **103**: 286–91.

146 LAI C-H, MALES BM, DOUGHERTY PA, BERTHOLD P, LISTGARTEN MA. Centipeda periodontii gen. nov., sp. nov. from Human Periodontal Lesions. *International Journal of Systematic and Evolutionary Microbiology,* 1983; **33**: 628–35.

147 Scholz T, Salgado-Maldonado G. The Introduction and Dispersal of Centrocestus formosanus (Nishigori, 1924) (Digenea: Heterophyidae) in Mexico: A Review. *amid* 2000; **143**: 185–200.

148 Han ET, Shin EH, Phommakorn S, *et al.* Centrocestus formosanus (Digenea: Heterophyidae) encysted in the freshwater fish, Puntius brevis, from Lao PDR. *Korean J Parasitol* 2008; **46**: 49–53.

149 Chai J-Y, Sohn W-M, Yong T-S, *et al.* Centrocestus formosanus (Heterophyidae): Human Infections and the Infection Source in Lao PDR. *para* 2013; **99**: 531–6.

150 Guppy KH, Thomas C, Thomas K, Anderson D. Cerebral Fungal Infections in the Immunocompromised Host: A Literature Review and a New Pathogen—Chaetomium atrobrunneum: Case Report. *Neurosurgery* 1998; **43**: 1463–9.

151 Barron MA, Sutton DA, Veve R, *et al.* Invasive Mycotic Infections Caused by Chaetomium perlucidum, a New Agent of Cerebral Phaeohyphomycosis. *Journal of Clinical Microbiology* 2003; **41**: 5302–7.

152 Brewis C, McFerran DJ. 'Farmer's ear': sudden sensorineural hearing loss due to Chlamydia psittaci infection. *The Journal of Laryngology & Otology* 1997; **111**: 855–7.

153 Vanrompay D, Ducatelle R, Haesebrouck F. Chlamydia psittaci infections: a review with emphasis on avian chlamydiosis. *Vet Microbiol* 1995; **45**: 93–119.

154 Malhotra M, Sood S, Mukherjee A, Muralidhar S, Bala M. Genital Chlamydia trachomatis: An update. *Indian J Med Res* 2013; **138**: 303–16.

155 Thomas PA, Geraldine P. Oculomycosis. In: Topley & Wilson's Microbiology and Microbial Infections. American Cancer Society, 2010. DOI:10.1002/9780470688618.taw0143.

156 Szabo NJ, Matulka RA, Chan T. Safety evaluation of Whole Algalin Protein (WAP) from Chlorella protothecoides. *Food Chem Toxicol* 2013; **59**: 34–45.

157 Hoque SN, Graham J, Kaufmann ME, Tabaqchali S. Chryseobacterium (Flavobacterium) meningosepticum outbreak associated with colonization of water taps in a neonatal intensive care unit. *J Hosp Infect* 2001; **47**: 188–92.

158 Dzeing-Ella A, Szwebel TA, Loubinoux J, *et al.* Infective endocarditis due to Citrobacter koseri in an immunocompetent adult. *J Clin Microbiol* 2009; **47**: 4185–6.

159 Markova IA, Romanenko AS, Dukhanina AV. [Isolation of bacteria of the family enterobacteriaceae from plant tissues]. *Mikrobiologiia* 2005; **74**: 663–6.

160 Borenshtein D, Schauer DB. The Genus Citrobacter. In: Dworkin M, Falkow S, Rosenberg E, Schleifer K-H, Stackebrandt E, eds. The Prokaryotes. Springer New York, 2006: 90–8.

161 Doran TI. The Role of Citrobacter in Clinical Disease of Children: Review. *Clin Infect Dis* 1999; **28**: 384–94.

162 Chopin JB, Sigler L, Connole MD, O'Boyle DA, Mackay B, Goldstein L. Keratomycosis in a Percheron cross horse caused by Cladorrhinum bulbillosum. *J Med Vet Mycol* 1997; **35**: 53–5.

163 Zapater RC, Scattini F. Mycotic keratitis by Cladorrhinum. *Sabouraudia* 1979; **17**: 65–9.

164 Namratha N, Nadgir S, Kale M, Rathod R. Chromoblastomycosis due to Cladosporium carrionii. *J Lab Physicians* 2010; **2**: 47–8.

165 Chung DI, Moon CH, Kong HH, Choi DW, Lim DK. The first human case of Clinostomum complanatum (Trematoda: Clinostomidae) infection in Korea. *Korean J Parasitol* 1995; **33**: 219–23.

166 Baldassi L. Clostridial toxins: potent poisons, potent medicines. *Journal of Venomous Animals and Toxins including Tropical Diseases* 2005; **11**: 391–411.

167 Shapiro RL, Hatheway C, Swerdlow DL. Botulism in the United States: a clinical and epidemiologic review. *Ann Intern Med* 1998; **129**: 221–8.

168 Marsh JW, O'Leary MM, Shutt KA, *et al.* Multilocus Variable-Number Tandem-Repeat Analysis for Investigation of Clostridium difficile Transmission in Hospitals. *Journal of Clinical Microbiology* 2006; **44**: 2558–66.

169 Viswanathan VK, Mallozzi MJ, Vedantam G. Clostridium difficile infection: An overview of the disease and its pathogenesis, epidemiology and interventions. *Gut Microbes* 2010; **1**: 234–42.

170 Shimizu T, Ohtani K, Hirakawa H, *et al.* Complete genome sequence of Clostridium perfringens, an anaerobic flesh-eater. *Proc Natl Acad Sci USA* 2002; **99**: 996–1001.

171 Cooper KK, Songer JG. Virulence of Clostridium perfringens in an experimental model of poultry necrotic enteritis. *Vet Microbiol* 2010; **142**: 323–8.

172 Shoesmith JG, Holland KT. The germination of spores of Clostridium tetani. *J Gen Microbiol* 1972; **70**: 253–61.

173 Hatheway CL. Toxigenic clostridia. *Clin Microbiol Rev* 1990; **3**: 66–98.

174 Emmons, Ashburn. THE ISOLATION OF HAPLOSPORANGIUM PARVUM N. SPAND COCCIDIOIDES IMMITIS FROM WILD RODENTS. THEIR RELATIONSHIP TO COCCIDIOIDOMYCOSIS. *Public Health Rep* 1942; **57**: 1715–46.

175 Kemna ME, Neri RC, Ali R, Salkin IF. Cokeromyces recurvatus, a mucoraceous zygomycete rarely isolated in clinical laboratories. *J Clin Microbiol* 1994; **32**: 843–5.

176 Romero JR, Simonsen KA. Powassan Encephalitis and Colorado Tick Fever. *Infectious disease clinics of North America* 2008; **22**: 545–59.

177 Moutailler S, Popovici I, Devillers E, Vayssier-Taussat M, Eloit M. Diversity of viruses in Ixodes ricinus, and characterization of a neurotropic strain of Eyach virus. *New Microbes and New Infections* 2016; **11**: 71–81.

178 Walsh TJ, Renshaw G, Andrews J, *et al.* Invasive Zygomycosis Due to Conidiobolus incongruus. *Clin Infect Dis* 1994; **19**: 423–30.

179 Kimura M, Yaguchi T, Sutton DA, Fothergill AW, Thompson EH, Wickes BL. Disseminated Human Conidiobolomycosis Due to Conidiobolus lamprauges. *J Clin Microbiol* 2011; **49**: 752–6.

180 Taylor GD, Sekhon AS, Tyrrell DLJ, Goldsand G. Rhinofacial Zygomycosis Caused by Conidiobolus coronatus: A Case Report Including in Vitro Sensitivity to Antimycotic Agents. *The American Journal of Tropical Medicine and Hygiene* 1987; **36**: 398–401.

181 Nayak DR, Pillai S, Rao L. Rhinofacial zygomycosis caused by conidiobolus coronatus. *Indian J Otolaryngol Head Neck Surg* 2004; **56**: 225–7.

182 Mandlik A, Swierczynski A, Das A, Ton-That H. Corynebacterium diphtheriae employs specific minor pilins to target human pharyngeal epithelial cells. *Mol Microbiol* 2007; **64**: 111–24.

183 Funke G, von Graevenitz A, Clarridge JE, Bernard KA. Clinical microbiology of coryneform bacteria. *Clin Microbiol Rev* 1997; **10**: 125–59.

184 Manzella JP, Kellogg JA, Parsey KS. Corynebacterium pseudodiphtheriticum: a respiratory tract pathogen in adults. *Clin Infect Dis* 1995; **20**: 37–40.

185 Soriano F, Zapardiel J, Nieto E. Antimicrobial susceptibilities of Corynebacterium species and other non-spore-forming gram-positive bacilli to 18 antimicrobial agents. *Antimicrob Agents Chemother* 1995; **39**: 208–14.

186 Bittar F, Cassagne C, Bosdure E, *et al.* Outbreak of Corynebacterium pseudodiphtheriticum Infection in Cystic Fibrosis Patients, France. *Emerg Infect Dis* 2010; **16**: 1231–6.

187 Leonard RB, Nowowiejski DJ, Warren JJ, Finn DJ, Coyle MB. Molecular evidence of person-to-person transmission of a pigmented strain of Corynebacterium striatum in intensive care units. *J Clin Microbiol* 1994; **32**: 164–9.

188 Otsuka Y, Ohkusu K, Kawamura Y, Baba S, Ezaki T, Kimura S. Emergence of multidrug-resistant Corynebacterium striatum as a nosocomial pathogen in long-term hospitalized patients with underlying diseases. *Diagnostic Microbiology and Infectious Disease* 2006; **54**: 109–14.

189 Jefferies DJ, Hanson HM, Harris EA. The prevalence of Pseudoterranova decipiens (Nematoda) and Corynosoma strumosum (Acanthocephala) in otters Lutra lutra from coastal sites in Britain. *Journal of Zoology* 1990; **221**: 316–21.

190 Seshadri R, Paulsen IT, Eisen JA, *et al.* Complete genome sequence of the Q-fever pathogen Coxiella burnetii. *Proc Natl Acad Sci USA* 2003; **100**: 5455–60.

191 Maurin M, Raoult D. Q fever. *Clin Microbiol Rev* 1999; **12**: 518–53.

192 Mitchell TG, Perfect JR. Cryptococcosis in the era of AIDS--100 years after the discovery of Cryptococcus neoformans. *Clin Microbiol Rev* 1995; **8**: 515–48.

193 Xiao L. Molecular epidemiology of cryptosporidiosis: an update. *Exp Parasitol* 2010; **124**: 80–9.

194 Chalmers RM, Davies AP. Minireview: clinical cryptosporidiosis. *Exp Parasitol* 2010; **124**: 138–46.

195 Current WL, Garcia LS. Cryptosporidiosis. *Clin Microbiol Rev* 1991; **4**: 325–58.

196 Bibashi E, Sidi V, Kotsiou M, Makrigiannaki E, Koliouskas D. Pulmonary Zygomycosis caused by Cunninghamella bertholletiae in a child with acute lymphoblastic leukemia. *Hippokratia* 2008; **12**: 43–5.

197 Nityananda K, Sivasubramaniam P, Ajello L. A CASE OF MYCOTIC KERATITIS CAUSED BY CURVULARIA GENICULATA. *Arch Ophthalmol* 1964; **71**: 456–8.

198 Rinaldi MG, Phillips P, Schwartz JG, *et al.* Human Curvularia infections. Report of five cases and review of the literature. *Diagn Microbiol Infect Dis* 1987; **6**: 27–39.

199 Hasegawa H, Syafruddin null. Cyclodontostomum purvisi (syn. Ancistronema coronatum) (Nematoda: Strongyloidea: Chabertiidae) from rats of Kalimantan and Sulawesi, Indonesia. *J Parasitol* 1994; **80**: 657–60.

200 Bhaibulaya M, Indrangarm S. Man, an accidental host of Cyclodontostomum purvisi (Adams, 1933), and the occurrence in rats in Thailand. *Southeast Asian J Trop Med Public Health* 1975; **6**: 391–4.

201 Chaisiri K, Siribat P, Ribas A, Morand S. Potentially Zoonotic Helminthiases of Murid Rodents from the Indo-Chinese Peninsula: Impact of Habitat and the Risk of Human Infection. *Vector Borne Zoonotic Dis* 2015; **15**: 73–85.

202 Ortega YR, Sanchez R. Update on Cyclospora cayetanensis, a Food-Borne and Waterborne Parasite. *CMR* 2010; **23**: 218–34.

203 Zoutman DE, Sigler L. Mycetoma of the foot caused by Cylindrocarpon destructans. *J Clin Microbiol* 1991; **29**: 1855–9.

204 Britt W. Manifestations of human cytomegalovirus infection: proposed mechanisms of acute and chronic disease. *Curr Top Microbiol Immunol* 2008; **325**: 417–70.

205 Kawamura I, Yagi T, Hatakeyama K, *et al.* Recurrent vascular catheter-related bacteremia caused by Delftia acidovorans with different antimicrobial susceptibility profiles. *J Infect Chemother* 2011; **17**: 111–3.

206 Lee SM, Kim MK, Lee JL, Wee WR, Lee JH. Experience of Comamonas Acidovorans Keratitis with Delayed Onset and Treatment Response in Immunocompromised Cornea. *Korean Journal of Ophthalmology* 2008; **22**: 49–52.

207 Sodroski JG, Rosen CA, Haseltine WA. Trans-acting transcriptional activation of the long terminal repeat of human T lymphotropic viruses in infected cells. *Science* 1984; **225**: 381–5.

208 Cengiz ZT, Yilmaz H, Dulger AC, Cicek M. Human infection with Dicrocoelium dendriticum in Turkey. *Ann Saudi Med* 2010; **30**: 159–61.

209 Gonçalves MLC, Araújo A, Ferreira LF. Human intestinal parasites in the past: new findings and a review. *Memórias do Instituto Oswaldo Cruz* 2003; **98**: 103–18.

210 Skeríková A, Brabec J, Kuchta R, Jiménez JA, García HH, Scholz T. Is the human-infecting Diphyllobothrium pacificum a valid species or just a South American population of the holarctic fish broad tapeworm, D. latum? *Am J Trop Med Hyg* 2006; **75**: 307–10.

211 Gutierrez Y, Catallaer M, Wicker D. Extrapulmonary Dirofilaria Immitis-like Infections in the Western Hemisphere. *The American Journal of Surgical Pathology* 1996; **20**: 299–305.

212 Diaz JH, Risher WH. Risk factors for human heartworm infections (dirofilariasis) in the South. *J La State Med Soc* 2015; **167**: 79–86.

213 Reddy MV. Human dirofilariasis: An emerging zoonosis. *Trop Parasitol* 2013; **3**: 2–3.

214 Beguin H, Nolard N. Mould biodiversity in homes I. Air and surface analysis of 130 dwellings. *Aerobiologia* 1994; **10**: 157.

215 Ali-Shtayeh MS, Salameh A-AM, Abu-Ghdeib S, Jamous RM. Hair and scalp mycobiota in school children in Nablus area. *Mycopathologia* 2001; **150**: 127–35.

216 Muller R, Muller R, Wakelin D. Worms and human disease, 2nd ed. Wallingford, Oxon, UK; New York: CABI, 2002.

217 Pourrut X, Kumulungui B, Wittmann T, *et al.* The natural history of Ebola virus in Africa. *Microbes and Infection* 2005; **7**: 1005–14.

218 Miranda MEG, Miranda NLJ. Reston ebolavirus in humans and animals in the Philippines: a review. *J Infect Dis* 2011; **204 Suppl 3**: S757-760.

219 Chai J-Y. Echinostomes in humans. In: Toledo R, Fried B, eds. The Biology of Echinostomes. New York, NY: Springer New York, 2009: 147–83.

220 Lee SK, Chung NS, Ko IH, Ko HI, Sohn WM. [A case of natural human infection by Echinostoma cinetorchis]. *Kisaengchunghak Chapchi* 1988; **26**: 61–4.

221 Toledo R, Esteban JG. An update on human echinostomiasis. *Trans R Soc Trop Med Hyg* 2016; **110**: 37–45.

222 Cho C-M, Tak W-Y, Kweon Y-O, *et al.* A human case of Echinostoma hortense (Trematoda: Echinostomatidae) infection diagnosed by gastroduodenal endoscopy in Korea. *Korean J Parasitol* 2003; **41**: 117–20.

223 Lo CT. Echinostoma macrorchis: life history, population dynamics of intramolluscan stages, and the first and second intermediate hosts. *J Parasitol* 1995; **81**: 569–76.

224 Tangtrongchitr A, Monzon RB. Eating habits associated with Echinostoma malayanum infections in the Philippines. *Southeast Asian J Trop Med Public Health* 1991; **22 Suppl**: 212–6.

225 Chantima K, Chai J-Y, Wongsawad C. Echinostoma revolutum: freshwater snails as the second intermediate hosts in Chiang Mai, Thailand. *Korean J Parasitol* 2013; **51**: 183–9.

226 Grover M, Dutta R, Kumar R, Aneja S, Mehta G. Echinostoma ilocanum infection. *Indian Pediatr* 1998; **35**: 549–52.

227 Paddock CD, Childs JE. Ehrlichia chaffeensis: a Prototypical Emerging Pathogen. *CMR* 2003; **16**: 37–64.

228 Dumler JS, Asanovich KM, Bakken JS, Richter P, Kimsey R, Madigan JE. Serologic cross-reactions among Ehrlichia equi, Ehrlichia phagocytophila, and human granulocytic Ehrlichia. *Journal of Clinical Microbiology* 1995; **33**: 1098–103.

229 Dumler JS, Walker DH. Tick-borne ehrlichioses. *The Lancet Infectious Diseases* 2001; **1**: 21–8.

230 Emmons CW, Jellison WL. Emmonsia crescens sp. n. and adiaspiromycosis (haplomycosis) in mammals. *Ann N Y Acad Sci* 1960; **89**: 91–101.

231 Anstead GM, Sutton DA, Graybill JR. Adiaspiromycosis Causing Respiratory Failure and a Review of Human Infections Due to Emmonsia and Chrysosporium spp. *J Clin Microbiol* 2012; **50**: 1346–54.

232 Weber R, Kuster H, Visvesvara GS, Bryan RT, Schwartz DA, Lüthy R. Disseminated Microsporidiosis Due to Encephalitozoon hellem: Pulmonary Colonization, Microhematuria, and Mild Conjunctivitis in a Patient with AIDS. *Clin Infect Dis* 1993; **17**: 415–9.

233 Didier ES, Weiss LM. Microsporidiosis: not just in AIDS patients. *Curr Opin Infect Dis* 2011; **24**: 490–5.

234 Valencakova A, Balent P, Ravaszova P, *et al.* Molecular identification and genotyping of Microsporidia in selected hosts. *Parasitol Res* 2012; **110**: 689–93.

235 Loftus B, Anderson I, Davies R, *et al.* The genome of the protist parasite Entamoeba histolytica. *Nature* 2005; **433**: 865–8.

236 Westerfeld C, Papaliodis GN, Behlau I, Durand ML, Sobrin L. Enterobacter amnigenus endophthalmitis. *Retin Cases Brief Rep* 2009; **3**: 409–11.

237 Mezzatesta ML, Gona F, Stefani S. Enterobacter cloacae complex: clinical impact and emerging antibiotic resistance. *Future Microbiol* 2012; **7**: 887–902.

238 Cook GC. Enterobius vermicularis infection. *Gut* 1994; **35**: 1159–62.

239 Byappanahalli MN, Nevers MB, Korajkic A, Staley ZR, Harwood VJ. Enterococci in the Environment. *Microbiol Mol Biol Rev* 2012; **76**: 685–706.

240 Morrison D, Woodford N, Cookson B. Enterococci as emerging pathogens of humans. *Journal of Applied Microbiology* 1997; **83**: 89S-99S.

241 Boyce JM. VANCOMYCIN-RESISTANT ENTEROCOCCUS: Detection, Epidemiology, and Control Measures. *Infectious Disease Clinics of North America* 1997; **11**: 367–84.

242 Dowd SE, Gerba CP, Pepper IL. Confirmation of the Human-Pathogenic Microsporidia Enterocytozoon bieneusi, Encephalitozoon intestinalis, and Vittaforma corneae in Water. *Appl Environ Microbiol* 1998; **64**: 3332–5.

243 Kee F, McElroy G, Stewart D, Coyle P, Watson J. A community outbreak of echovirus infection associated with an outdoor swimming pool. *J Public Health (Oxf)* 1994; **16**: 145–8.

244 Kramer M, Schulte BM, Toonen LWJ, *et al.* Echovirus infection causes rapid loss-of-function and cell death in human dendritic cells. *Cell Microbiol* 2007; **9**: 1507–18.

245 Hall CB, Cherry JD, Hatch MH, Nelson DB, Winter HS. The Return of Boston Exanthem: Echovirus 16 Infections in 1974. *Am J Dis Child* 1977; **131**: 323–6.

246 Zhang Y, Tan X-J, Wang H-Y, *et al.* An outbreak of hand, foot, and mouth disease associated with subgenotype C4 of human enterovirus 71 in Shandong, China. *Journal of Clinical Virology* 2009; **44**: 262–7.

247 Mauleekoonphairoj J, Puenpa J, Korkong S, Vongpunsawad S, Poovorawan Y. PREVALENCE OF HUMAN ENTEROVIRUS AMONG PATIENTS WITH HAND, FOOT, AND MOUTH DISEASE AND HERPANGINA IN THAILAND, 2013. *Southeast Asian J Trop Med Public Health* 2015; **46**: 1013–20.

248 Dowdle WR, Birmingham ME. The Biologic Principles of Poliovirus Eradication. *Journal of Infectious Diseases* 1997; **175**: S286–92.

249 Aly R. Ecology and epidemiology of dermatophyte infections. *J Am Acad Dermatol* 1994; **31**: S21-25.

250 Brooke CJ, Riley TV. Erysipelothrix rhusiopathiae: bacteriology, epidemiology and clinical manifestations of an occupational pathogen. *J Med Microbiol* 1999; **48**: 789–99.

251 Reboli AC, Farrar WE. Erysipelothrix rhusiopathiae: an occupational pathogen. *Clin Microbiol Rev* 1989; **2**: 354–9.

252 Caprioli A, Morabito S, Brug�re H, Oswald E. Enterohaemorrhagic *Escherichia coli* : emerging issues on virulence and modes of transmission. *Vet Res* 2005; **36**: 289–311.

253 Solomon EB, Yaron S, Matthews KR. Transmission of Escherichia coli O157:H7 from Contaminated Manure and Irrigation Water to Lettuce Plant Tissue and Its Subsequent Internalization. *AEM* 2002; **68**: 397–400.

254 Hill GB, Ayers OM, Kohan AP. Characteristics and sites of infection of Eubacterium nodatum, Eubacterium timidum, Eubacterium brachy, and other asaccharolytic eubacteria. *J Clin Microbiol* 1987; **25**: 1540–5.

255 Rochford JC. Pleuropulmonary infection associated with Eubacterium brachy, a new species of Eubacterium. *J Clin Microbiol* 1980; **12**: 722–3.

256 Finegold SM, Sutter VL. Fecal flora in different populations, with special reference to diet. *Am J Clin Nutr* 1978; **31**: S116–22.

257 Schwiertz A, Le Blay G, Blaut M. Quantification of different Eubacterium spp. in human fecal samples with species-specific 16S rRNA-targeted oligonucleotide probes. *Appl Environ Microbiol* 2000; **66**: 375–82.

258 Rimawi BH, Rimawi RH, Mirdamadi M, *et al.* A case of Exophiala oligosperma successfully treated with voriconazole. *Medical Mycology Case Reports* 2013; **2**: 144–7.

259 Al-Obaid I, Ahmad S, Khan ZU, Dinesh B, Hejab HM. Catheter-associated fungemia due to Exophiala oligosperma in a leukemic child and review of fungemia cases caused by Exophiala species. *European Journal of Clinical Microbiology & Infectious Diseases* 2006; **25**: 729–32.

260 Wang L, Yokoyama K, Miyaji M, Nishimura K. Identification, Classification, and Phylogeny of the Pathogenic Species Exophiala jeanselmei and Related Species by Mitochondrial Cytochrome b Gene Analysis. *J Clin Microbiol* 2001; **39**: 4462–7.

261 Nucci M, Akiti T, Barreiros G, *et al.* Nosocomial outbreak of Exophiala jeanselmei fungemia associated with contamination of hospital water. *Clin Infect Dis* 2002; **34**: 1475–80.

262 Phalee A, Wongsawad C, Rojanapaibul A, Chai J-Y. Experimental life history and biological characteristics of Fasciola gigantica (Digenea: Fasciolidae). *Korean J Parasitol* 2015; **53**: 59–64.

263 Graczyk TK, Gilman RH, Fried B. Fasciolopsiasis: is it a controllable food-borne disease? *Parasitol Res* 2001; **87**: 80–3.

264 Montgomery L, Flesher B, Stahl D. Transfer of Bacteroides succinogenes (Hungate) to Fibrobacter gen. nov. as Fibrobacter succinogenes comb. nov. and Description of Fibrobacter intestinalis sp. nov. *International Journal of Systematic Bacteriology* 1988; **38**: 430–5.

265 Bogovic P, Strle F. Tick-borne encephalitis: A review of epidemiology, clinical characteristics, and management. *World J Clin Cases* 2015; **3**: 430–41.

266 Thomas SJ, Strickman D, Vaughn DW. Dengue Epidemiology: Virus Epidemiology, Ecology, and Emergence. In: Chambers TJ, Monath TP, eds. Advances in Virus Research. Academic Press, 2003: 235–89.

267 Kautner I, Robinson MJ, Kuhnle U. Dengue virus infection: Epidemiology, pathogenesis, clinical presentation, diagnosis, and prevention. *The Journal of Pediatrics* 1997; **131**: 516–24.

268 Diaz JH. Emerging Tickborne Viral Infections: What Wilderness Medicine Providers Need to Know. *Wilderness & Environmental Medicine* 2020; **31**: 489–97.

269 Mackenzie JS, Gubler DJ, Petersen LR. Emerging flaviviruses: the spread and resurgence of Japanese encephalitis, West Nile and dengue viruses. *Nat Med* 2004; **10**: S98–109.

270 Scherret JH, Poidinger M, Mackenzie JS, *et al.* The relationships between West Nile and Kunjin viruses. *Emerg Infect Dis* 2001; **7**: 697–705.

271 Holbrook MR. Kyasanur forest disease. *Antiviral Research* 2012; **96**: 353–62.

272 Davidson MM, Williams H, Macleod JA. Louping ill in man: a forgotten disease. *J Infect* 1991; **23**: 241–9.

273 Hudson PJ, Norman R, Laurenson MK, *et al.* Persistence and transmission of tick-borne viruses: *Ixodes ricinus* and louping-ill virus in red grouse populations. *Parasitology* 1995; **111**: S49–58.

274 Selvey LA, Dailey L, Lindsay M, *et al.* The changing epidemiology of Murray Valley encephalitis in Australia: the 2011 outbreak and a review of the literature. *PLoS Negl Trop Dis* 2014; **8**: e2656.

275 Růžek D, Yakimenko VV, Karan LS, Tkachev SE. Omsk haemorrhagic fever. *The Lancet* 2010; **376**: 2104–13.

276 Anderson JF, Armstrong PM. Prevalence and genetic characterization of Powassan virus strains infecting Ixodes scapularis in Connecticut. *Am J Trop Med Hyg* 2012; **87**: 754–9.

277 Medeiros DBA, Nunes MRT, Vasconcelos PFC, Chang G-JJ, Kuno G. Complete genome characterization of Rocio virus (Flavivirus: Flaviviridae), a Brazilian flavivirus isolated from a fatal case of encephalitis during an epidemic in São Paulo state. *Journal of General Virology,* 2007; **88**: 2237–46.

278 Thomas SJ, Endy TP, Rothman AL, Barrett AD. Flaviviruses (Dengue, Yellow Fever, Japanese Encephalitis, West Nile Encephalitis, St. Louis Encephalitis, Tick-Borne Encephalitis, Kyasanur Forest Disease, Alkhurma Hemorrhagic Fever, Zika). In: Mandell, Douglas, and Bennett's Principles and Practice of Infectious Diseases. Elsevier, 2015: 1881-1903.e6.

279 Rossi SL, Ross TM, Evans JD. West Nile virus. *Clin Lab Med* 2010; **30**: 47–65.

280 Monath TP. Yellow fever: an update. *Lancet Infect Dis* 2001; **1**: 11–20.

281 Vickers RM, Yu VL. Clinical laboratory differentiation of Legionellaceae family members with pigment production and fluorescence on media supplemented with aromatic substrates. *J Clin Microbiol* 1984; **19**: 583–7.

282 Brown A, Garrity GM, Vickers RM. Fluoribacter dumoffii (Brenner et al.) comb. nov. and Fluoribacter gormanii (Morris et al.) comb. nov. *International Journal of Systematic Bacteriology* 1981; **31**: 111–5.

283 Sharma NL, Sharma RC, Grover PS, Gupta ML, Sharma AK, Mahajan VK. Chromoblastomycosis in India. *Int J Dermatol* 1999; **38**: 846–51.

284 Chaidaroon W, Tananuvat N, Chavengsaksongkram P, Vanittanakom N. Corneal Chromoblastomycosis Caused by Fonsecaea pedrosoi. *Case Rep Ophthalmol* 2015; **6**: 82–7.

285 Keim P, Johansson A, Wagner DM. Molecular epidemiology, evolution, and ecology of Francisella. *Ann N Y Acad Sci* 2007; **1105**: 30–66.

286 Santic M, Al-Khodor S, Abu Kwaik Y. Cell biology and molecular ecology of Francisella tularensis. *Cell Microbiol* 2010; **12**: 129–39.

287 Larsson P, Oyston PCF, Chain P, *et al.* The complete genome sequence of Francisella tularensis, the causative agent of tularemia. *Nat Genet* 2005; **37**: 153–9.

288 Zhang S, Ahearn DG, Noble-Wang JA, *et al.* Growth and Survival of Fusarium solani-F. oxysporum Complex on Stressed Multipurpose Contact Lens Care Solution Films on Plastic Surfaces In Situ and In Vitro. *Cornea* 2006; **25**: 1210–6.

289 Godoy P, Cano J, Gené J, Guarro J, Höfling-Lima AL, Lopes Colombo A. Genotyping of 44 Isolates of Fusarium solani, the Main Agent of Fungal Keratitis in Brazil. *J Clin Microbiol* 2004; **42**: 4494–7.

290 Prout J, Glymph R. Anaerobic septicemia secondary to Fusobacterium mortiferum. *J Natl Med Assoc* 1986; **78**: 334, 337.

291 Catlin BW. Gardnerella vaginalis: characteristics, clinical considerations, and controversies. *Clin Microbiol Rev* 1992; **5**: 213–37.

292 Uzairue L, Ugbor C, Ezeah G, Eze N, Nwadike I. Gastrodiscoides hominis Infestation on Vegetables (Cabbages) Sold in Ekpoma Markets, Edo State, Southern Nigeria- A Case Report. *International Journal of Basic, Applied and Innovative Research*; **2**: 37–9.

293 Kassamali H, Anaissie E, Ro J, *et al.* Disseminated Geotrichum candidum infection. *Journal of Clinical Microbiology* 1987; **25**: 1782–3.

294 Sfakianakis A, Krasagakis K, Stefanidou M, *et al.* Invasive cutaneous infection with Geotrichum candidum – sequential treatment with amphotericin B and voriconazole. *Med Mycol* 2007; **45**: 81–4.

295 Faubert G. Immune response to Giardia duodenalis. *Clin Microbiol Rev* 2000; **13**: 35–54, table of contents.

296 Nawa Y. Historical review and current status of gnathostomiasis in Asia. *Southeast Asian J Trop Med Public Health* 1991; **22 Suppl**: 217–9.

297 Nawa Y, Imai J, Ogata K, Otsuka K. The First Record of a Confirmed Human Case of Gnathostoma doloresi Infection. *The Journal of Parasitology* 1989; **75**: 166–9.

298 Prommas C, Daengsvang S. Further Report of a Study on the Life Cycle of Gnathostoma spinigerum. *The Journal of Parasitology* 1936; **22**: 180–6.

299 Punyagupta S, Bunnag T, Juttijudata P. Eosinophilic meningitis in Thailand. Clinical and epidemiological characteristics of 162 patients with myeloencephalitis probably caused by Gnathostoma spinigerum. *J Neurol Sci* 1990; **96**: 241–56.

300 Wilde H, Suankratay C, Thongkam C, Chaiyabutr N. Human Gongylonema Infection in Southeast Asia. *J Travel Med* 2001; **8**: 204–6.

301 Blanc V, Dalle M, Markarian A, *et al.* Gordonia terrae: a Difficult-To-Diagnose Emerging Pathogen? *J Clin Microbiol* 2007; **45**: 1076–7.

302 Price T, Edwards L. Haemophilus aphrophilus endocarditis. *J Okla State Med Assoc* 1984; **77**: 387–9.

303 Batshon BA, Brosius OC. A case of cerebral abscess with haemophilus aphrophilus cultured from spinal fluid. *Am J Clin Pathol* 1969; **52**: 356–7.

304 Bieger RC, Brewer NS, Washington JA. Haemophilus aphrophilus: a microbiologic and clinical review and report of 42 cases. *Medicine (Baltimore)* 1978; **57**: 345–55.

305 Lewis DA. Chancroid: clinical manifestations, diagnosis, and management. *Sex Transm Infect* 2003; **79**: 68–71.

306 Morse SA. Chancroid and Haemophilus ducreyi. *Clin Microbiol Rev* 1989; **2**: 137–57.

307 Makintubee S, Istre GR, Ward JI. Transmission of invasive Haemophilus influenzae type b disease in day care settings. *The Journal of Pediatrics* 1987; **111**: 180–6.

308 Martinez VP, Bellomo C, San Juan J, *et al.* Person-to-person transmission of Andes virus. *Emerging Infect Dis* 2005; **11**: 1848–53.

309 Lundkvist A, Hukic M, Hörling J, Gilljam M, Nichol S, Niklasson B. Puumala and Dobrava viruses cause hemorrhagic fever with renal syndrome in Bosnia-Herzegovina: evidence of highly cross-neutralizing antibody responses in early patient sera. *J Med Virol* 1997; **53**: 51–9.

310 Lee HW, Baek LJ, Johnson KM. Isolation of Hantaan Virus, the Etiologic Agent of Korean Hemorrhagic Fever, from Wild Urban Rats. *The Journal of Infectious Diseases* 1982; **146**: 638–44.

311 Goldsmith CS, Elliott LH, Peters CJ, Zaki SR. Ultrastructural characteristics of Sin Nombre virus, causative agent of hantavirus pulmonary syndrome. *Archives of Virology* 1995; **140**: 2107–22.

312 Miliotis M. International Handbook of Foodborne Pathogens. CRC Press. https://www.crcpress.com/International-Handbook-of-Foodborne-Pathogens/Miliotis-Bier/p/book/9780824706852 (accessed March 21, 2020).

313 Suerbaum S, Michetti P. Helicobacter pylori infection. *N Engl J Med* 2002; **347**: 1175–86.

314 Salmanian A-H, Siavoshi F, Akbari F, Afshari A, Malekzadeh R. Yeast of the oral cavity is the reservoir of Heliobacter pylori: Oral yeast is the reservoir of H. pylori. *Journal of Oral Pathology & Medicine* 2008; **37**: 324–8.

315 Lima LR, Almeida AJD, Tourinho R dos S, Hasselmann B, Lewis Ximenez LL, De Paula VS. Evidence of Hepatitis A Virus Person-to-Person Transmission in Household Outbreaks. *PLoS ONE* 2014; **9**: e102925.

316 Chai JY, Seo BS, Lee SH, Hong SJ, Sohn WM. Human infections by Heterophyes heterophyes and H. dispar imported from Saudi Arabia. *Korean J Parasitol* 1986; **24**: 82.

317 Park J-H, Kim J-L, Shin E-H, Guk S-M, Park Y-K, Chai J-Y. A new endemic focus of Heterophyes nocens and other heterophyid infections in a coastal area of Gangjin-gun, Jeollanam-do. *Korean J Parasitol* 2007; **45**: 33.

318 Mas-Coma S, Bargues MD, Valero MA. Fascioliasis and other plant-borne trematode zoonoses. *Int J Parasitol* 2005; **35**: 1255–78.

319 Kauffman CA. Histoplasmosis: a clinical and laboratory update. *Clin Microbiol Rev* 2007; **20**: 115–32.

320 Bonifaz A, Badali H, de Hoog GS, *et al.* Tinea nigra by Hortaea werneckii, a report of 22 cases from Mexico. *Studies in Mycology* 2008; **61**: 77–82.

321 Kim MJ, Kim WH, Jung H-C, Chai J-W, Chai J-Y. Isospora belli Infection with Chronic Diarrhea in an Alcoholic Patient. *Korean J Parasitol* 2013; **51**: 207–12.

322 Lindsay DS, Dubey JP, Blagburn BL. Biology of Isospora spp. from humans, nonhuman primates, and domestic animals. *Clin Microbiol Rev* 1997; **10**: 19–34.

323 Lindsay DS, Dubey JP, Butler JM, Blagburn BL. Mechanical transmission of Toxoplasma gondii oocysts by dogs. *Vet Parasitol* 1997; **73**: 27–33.

324 Richens J. The diagnosis and treatment of donovanosis (granuloma inguinale). *Sexually Transmitted Infections* 1991; **67**: 441–52.

325 O'Farrell N. Donovanosis. *Sex Transm Infect* 2002; **78**: 452–7.

326 Lowe C, Willey B, O'Shaughnessy A, *et al.* Outbreak of Extended-Spectrum β-Lactamase–producing Klebsiella oxytoca Infections Associated with Contaminated Handwashing Sinks1. *Emerg Infect Dis* 2012; **18**: 1242–7.

327 Nordmann P, Cuzon G, Naas T. The real threat of Klebsiella pneumoniae carbapenemase-producing bacteria. *Lancet Infect Dis* 2009; **9**: 228–36.

328 Taborda PR, Taborda VA, McGinnis MR. Lacazia loboi gen. nov., comb. nov., the etiologic agent of lobomycosis. *J Clin Microbiol* 1999; **37**: 2031–3.

329 Elsayed S, Kuhn SM, Barber D, Church DL, Adams S, Kasper R. Human Case of Lobomycosis. *Emerg Infect Dis* 2004; **10**: 715–8.

330 Mull N, Jackson R, Sironen T, Forbes KM. Ecology of Neglected Rodent-Borne American Orthohantaviruses. *Pathogens* 2020; **9**: 325.

331 Gubler JGH, Schorr M, Gaia V, Zbinden R, Altwegg M. Recurrent Soft Tissue Abscesses Caused by Legionella cincinnatiensis. *J Clin Microbiol* 2001; **39**: 4568–70.

332 Thacker WL, Dyke JW, Benson RF, *et al.* Legionella lansingensis sp. nov. isolated from a patient with pneumonia and underlying chronic lymphocytic leukemia. *J Clin Microbiol* 1992; **30**: 2398–401.

333 Tang PW, Toma S, MacMillan LG. Legionella oakridgensis: laboratory diagnosis of a human infection. *J Clin Microbiol* 1985; **21**: 462–3.

334 Fields BS, Benson RF, Besser RE. Legionella and Legionnaires' disease: 25 years of investigation. *Clin Microbiol Rev* 2002; **15**: 506–26.

335 Muder RR, Yu VL, Woo AH. Mode of transmission of Legionella pneumophila. A critical review. *Arch Intern Med* 1986; **146**: 1607–12.

336 Pearson RD, Sousa AQ. Clinical spectrum of Leishmaniasis. *Clin Infect Dis* 1996; **22**: 1–13.

337 Alvar J, Vélez ID, Bern C, *et al.* Leishmaniasis worldwide and global estimates of its incidence. *PLoS ONE* 2012; **7**: e35671.

338 Reithinger R. Cutaneous leishmaniasis. 2017; **7**. http://antimicrobe.org/h04c.files/history/LancetID_Reithinger_Cutaneous%20Leishmaniasis_2007.pdf (accessed March 21, 2020).

339 Pratlong F, Deniau M, Darie H, *et al.* Human cutaneous leishmaniasis caused by Leishmania naiffi is wide-spread in South America. *Ann Trop Med Parasitol* 2002; **96**: 781–5.

340 Bonfante-Garrido R, Meléndez E, Barroeta S, *et al.* Cutaneous leishmaniasis in western Venezuela caused by infection with Leishmania venezuelensis and L. braziliensis variants. *Transactions of the Royal Society of Tropical Medicine and Hygiene* 1992; **86**: 141–8.

341 Shaw JJ. New World Leishmaniasis: The Ecology of Leishmaniasis and the Diversity of Leishmanial Species in Central and South America. In: Farrell JP, ed. Leishmania. Boston, MA: Springer US, 2002: 11–31.

342 Gramiccia M, Gradoni L. The current status of zoonotic leishmaniases and approaches to disease control. *Int J Parasitol* 2005; **35**: 1169–80.

343 Levy JA. Pathogenesis of human immunodeficiency virus infection. *Microbiol Rev* 1993; **57**: 183–289.

344 Khatri ML, Al-Halali HM, Khalid MF, Saif SA, Vyas MCR. Mycetoma in Yemen: clinicoepidemiologic and histopathologic study. *International Journal of Dermatology* 2002; **41**: 586–93.

345 El-Ani AS. A New Species of Leptosphaeria, an Etiologic Agent of Mycetoma. *Mycologia* 1966; **58**: 406–11.

346 Bharti AR, Nally JE, Ricaldi JN, *et al.* Leptospirosis: a zoonotic disease of global importance. *The Lancet Infectious Diseases* 2003; **3**: 757–71.

347 Slack AT, Symonds ML, Dohnt MF, Smythe LD. The epidemiology of leptospirosis and the emergence of Leptospira borgpetersenii serovar Arborea in Queensland, Australia, 1998–2004. *Epidemiology & Infection* 2006; **134**: 1217–25.

348 Bulach DM, Zuerner RL, Wilson P, *et al.* Genome reduction in Leptospira borgpetersenii reflects limited transmission potential. *PNAS* 2006; **103**: 14560–5.

349 Matsunaga J, Lo M, Bulach DM, Zuerner RL, Adler B, Haake DA. Response of Leptospira interrogans to Physiologic Osmolarity: Relevance in Signaling the Environment-to-Host Transition. *Infect Immun* 2007; **75**: 2864–74.

350 de Faria MT, Calderwood MS, Athanazio DA, *et al.* Carriage of Leptospira interrogans among domestic rats from an urban setting highly endemic for leptospirosis in Brazil. *Acta Trop* 2008; **108**: 1–5.

351 Chadsuthi S, Bicout DJ, Wiratsudakul A, *et al.* Investigation on predominant Leptospira serovars and its distribution in humans and livestock in Thailand, 2010-2015. *PLOS Neglected Tropical Diseases* 2017; **11**: e0005228.

352 Chou L-F, Chen T-W, Ko Y-C, *et al.* Potential impact on kidney infection: a whole-genome analysis of Leptospira santarosai serovar Shermani. *Emerging Microbes & Infections* 2014; **3**: 1–11.

353 Hsieh W-J, Pan M-J. Identification Leptospira santarosai serovar shermani specific sequences by suppression subtractive hybridization. *FEMS Microbiol Lett* 2004; **235**: 117–24.

354 Farber JM, Peterkin PI. Listeria monocytogenes, a food-borne pathogen. *Microbiol Rev* 1991; **55**: 476–511.

355 Nufer U, Stephan R, Tasara T. Growth characteristics of Listeria monocytogenes, Listeria welshimeri and Listeria innocua strains in broth cultures and a sliced bologna-type product at 4 and 7 degrees C. *Food Microbiol* 2007; **24**: 444–51.

356 Andre P, Genicot A. [First isolation of Listeria welshimeri in a human]. *Zentralbl Bakteriol Mikrobiol Hyg A* 1987; **263**: 605–6.

357 Straus SE. Epstein-Barr Virus Infections: Biology, Pathogenesis, and Management. *Ann Intern Med* 1993; **118**: 45.

358 Kgaladi J, Wright N, Coertse J, *et al.* Diversity and epidemiology of Mokola virus. *PLoS Negl Trop Dis* 2013; **7**: e2511.

359 Dyer JL, Yager P, Orciari L, *et al.* Rabies surveillance in the United States during 2013. *J Am Vet Med Assoc* 2014; **245**: 1111–23.

360 Ahmed AOA, van Leeuwen W, Fahal A, van de Sande W, Verbrugh H, van Belkum A. Mycetoma caused by Madurella mycetomatis: a neglected infectious burden. *Lancet Infect Dis* 2004; **4**: 566–74.

361 Estrada R, Chávez-López G, Estrada-Chávez G, López-Martínez R, Welsh O. Eumycetoma. *Clinics in Dermatology* 2012; **30**: 389–96.

362 Guého E, Boekhout T, Ashbee HR, Guillot J, Van Belkum A, Faergemann J. The role of Malassezia species in the ecology of human skin and as pathogens. *Med Mycol* 1998; **36 Suppl 1**: 220–9.

363 Crespo Erchiga V, Ojeda Martos AA, Vera Casaño A, Crespo Erchiga A, Sánchez Fajardo F. [Isolation and identification of Malassezia spp. In pytiriasis versicolor, seborrheic dermatitis and healthy skin.]. *Rev Iberoam Micol* 1999; **16**: S16-21.

364 Belkum A van, Boekhout T, Bosboom R. Monitoring spread of Malassezia infections in a neonatal intensive care unit by PCR-mediated genetic typing. *Journal of Clinical Microbiology* 1994; **32**: 2528–32.

365 Bonthius DJ. Lymphocytic choriomeningitis virus: an underrecognized cause of neurologic disease in the fetus, child, and adult. *Semin Pediatr Neurol* 2012; **19**: 89–95.

366 Smith DH, Johnson BK, Isaacson M, *et al.* Marburg-virus disease in Kenya. *Lancet* 1982; **1**: 816–20.

367 Zehender G, Sorrentino C, Veo C, *et al.* Distribution of Marburg virus in Africa: An evolutionary approach. *Infect Genet Evol* 2016; **44**: 8–16.

368 Borkenhagen LK, Fieldhouse JK, Seto D, Gray GC. Are adenoviruses zoonotic? A systematic review of the evidence. *Emerging Microbes & Infections* 2019; **8**: 1679–87.

369 Desheva Y. Introductory Chapter: Human Adenoviruses. In: Desheva Y, ed. Adenoviruses. IntechOpen, 2019. DOI:10.5772/intechopen.82554.

370 Nama HS, Parihar A. Quantitative and qualitative analysis of helminth fauna in Rattus rattus rufescens. *J Helminthol* 1976; **50**: 99–102.

371 Lamon C, Greer GJ. Human infection with an anoplocephalid tapeworm of the genus Mathevotaenia. *Am J Trop Med Hyg* 1986; **35**: 824–6.

372 Fernando ST. Morphology, Systematics, and Geographic Distribution of Mecistocirrus digitatus, a Trichostrongylid Parasite of Ruminants. *The Journal of Parasitology* 1965; **51**: 149–55.

373 Van Aken D, Vercruysse J, Dargantes A, Lagapa J, Shaw DJ. Epidemiology of Mecistocirrus digitatus and other gastrointestinal nematode infections in cattle in Mindanao, Philippines. *Veterinary Parasitology* 1998; **74**: 29–41.

374 Esper F, Boucher D, Weibel C, Martinello RA, Kahn JS. Human metapneumovirus infection in the United States: clinical manifestations associated with a newly emerging respiratory infection in children. *Pediatrics* 2003; **111**: 1407–10.

375 MacLean JD, Ward BJ, Kokoskin E, Arthur JR, Gyorkos TW, Curtis MA. Common-source outbreak of acute infection due to the North American liver fluke Metorchis conjunctus. *The Lancet* 1996; **347**: 154–8.

376 Sandoval-Denis M, Gené J, Sutton DA, *et al.* Redefining Microascus, Scopulariopsis and allied genera. *Persoonia* 2016; **36**: 1–36.

377 Miossec C, Morio F, Lepoivre T, *et al.* Fatal invasive infection with fungemia due to Microascus cirrosus after heart and lung transplantation in a patient with cystic fibrosis. *J Clin Microbiol* 2011; **49**: 2743–7.

378 Onyiche TE, Okute TO, Oseni OS, Okoro DO, Biu AA, Mbaya AW. Parasitic and zoonotic meningoencephalitis in humans and equids: Current knowledge and the role of Halicephalobus gingivalis. *Parasite Epidemiology and Control* 2018; **3**: 36–42.

379 Kane J, Padhye AA, Ajello L. Microsporum equinum in North America. *J Clin Microbiol* 1982; **16**: 943–7.

380 Onsberg P. Human infections with Microsporum persicolor in Denmark. *Br J Dermatol* 1978; **99**: 531–6.

381 Allen DE, Snyderman R, Meadows L, Pinnell SR. Generalized Microsporum audouinii infection and depressed cellular immunity associated with a missing plasma factor required for lymphocyte blastogenesis. *The American Journal of Medicine* 1977; **63**: 991–1000.

382 Arenas R, Torres E, Amaya M, *et al.* Emergence of Microsporum audouinii and Trichophyton tonsurans as Causative Organisms of Tinea Capitis in the Dominican Republic. *Actas Dermo-Sifiliográficas (English Edition)* 2010; **101**: 330–5.

383 Sherwani S, Farleigh L, Agarwal N, *et al.* Seroprevalence of Molluscum contagiosum Virus in German and UK Populations. *PLoS ONE* 2014; **9**: e88734.

384 Karshima SN, Maikai B-V, Kwaga JKP. Helminths of veterinary and zoonotic importance in Nigerian ruminants: a 46-year meta-analysis (1970–2016) of their prevalence and distribution. *Infect Dis Poverty* 2018; **7**: 52.

385 McKenzie RA, Connole MD, McGinnis MR, Lepelaar R. Subcutaneous phaeohyphomycosis caused by Moniliella suaveolens in two cats. *Vet Pathol* 1984; **21**: 582–6.

386 Kodjo A, Richard Y, Tønjum T. Moraxella boevrei sp. nov., a new Moraxella species found in goats. *Int J Syst Bacteriol* 1997; **47**: 115–21.

387 Queen CW, Ward ACS, Hunter DL. BACTERIA ISOLATED FROM NASAL AND TONSILLAR SAMPLES OF CLINICALLY HEALTHY ROCKY MOUNTAIN BIGHORN AND DOMESTIC SHEEP. In: Journal of wildlife diseases. 1994. DOI:10.7589/0090-3558-30.1.1.

388 Christensen JJ, Renneberg J, Bruun B, Forsgren A. Serum antibody response to proteins of Moraxella (Branhamella) catarrhalis in patients with lower respiratory tract infection. *Clin Diagn Lab Immunol* 1995; **2**: 14–7.

389 Laksono B, de Vries R, McQuaid S, Duprex W, de Swart R. Measles Virus Host Invasion and Pathogenesis. *Viruses* 2016; **8**: 210.

390 Chandra S, Woodgyer A. Primary cutaneous zygomycosis due to Mucor circinelloides. *Australasian Journal of Dermatology* 2002; **43**: 39–42.

391 Karimi K, Zamani A. Mucor indicus: Biology and industrial application perspectives: A review. *Biotechnology Advances* 2013; **31**: 466–81.

392 Prevoo RLMA, Starink TM, Haan P de. Primary cutaneous mucormycosis in a healthy young girl: Report of a case caused by Mucor hiemalis Wehmer. *Journal of the American Academy of Dermatology* 1991; **24**: 882–5.

393 Webman RB, Gilman RH. 132 - Coenuriasis. In: Magill AJ, Hill DR, Solomon T, Ryan ET, eds. Hunter's Tropical Medicine and Emerging Infectious Disease (Ninth Edition). London: W.B. Saunders, 2013: 921–2.

394 Bourbeau P, McGough DA, Fraser H, Shah N, Rinaldi MG. Fatal disseminated infection caused by Myceliophthora thermophila, a new agent of mycosis: case history and laboratory characteristics. *J Clin Microbiol* 1992; **30**: 3019–23.

395 Aitken ML, Limaye A, Pottinger P, *et al.* Respiratory outbreak of Mycobacterium abscessus subspecies massiliense in a lung transplant and cystic fibrosis center. *Am J Respir Crit Care Med* 2012; **185**: 231–2.

396 Moore M, Frerichs JB. An unusual acid-fast infection of the knee with subcutaneous, abscess-like lesions of the gluteal region; report of a case with a study of the organism, Mycobacterium abscessus, n. sp. *J Invest Dermatol* 1953; **20**: 133–69.

397 Villanueva A, Villanueva Calderon R, Acosta Vargas B, *et al.* Report on an Outbreak of Postinjection Abscesses Due to Mycobacterium abscessus, Including Management with Surgery and Clarithromycin Therapy and Comparison of Strains by Random Amplified Polymorphic DNA Polymerase Chain Reaction. *Clin Infect Dis* 1997; **24**: 1147–53.

398 Taylor LQ, Williams AJ, Santiago S. Pulmonary disease caused by Mycobacterium asiaticum. *Tubercle* 1990; **71**: 303–5.

399 Butler WR, Plikaytis BB, Vadney FS, Gross WM. Mycobacterium celatum sp. nov. 2018; : 10.

400 Falkinham JO. Epidemiology of infection by nontuberculous mycobacteria. *Clin Microbiol Rev* 1996; **9**: 177–215.

401 Schulze-Röbbecke R, Feldmann C, Fischeder R, Janning B, Exner M, Wahl G. Dental units: an environmental study of sources of potentially pathogenic mycobacteria. *Tuber Lung Dis* 1995; **76**: 318–23.

402 Primm TP, Lucero CA, Falkinham JO. Health impacts of environmental mycobacteria. *Clin Microbiol Rev* 2004; **17**: 98–106.

403 Meyers H, Brown-Elliott BA, Moore D, *et al.* An outbreak of Mycobacterium chelonae infection following liposuction. *Clin Infect Dis* 2002; **34**: 1500–7.

404 Springer B, Tortoli E, Richter I, *et al.* Mycobacterium conspicuum sp. nov., a new species isolated from patients with disseminated infections. *Journal of clinical microbiology* 1995; **33**: 2805–11.

405 Mazumder SA, Hicks A, Norwood J. Mycobacterium gordonae pulmonary infection in an immunocompetent adult. *N Am J Med Sci* 2010; **2**: 205–7.

406 Engel HW, Berwald LG, Havelaar AH. The occurrence of Mycobacterium kansasii in tapwater. *Tubercle* 1980; **61**: 21–6.

407 Hsu P-Y, Yang Y-H, Hsiao C-H, Lee P-I, Chiang B-L. MYCOBACTERIUM KANSASII INFECTION PRESENTING AS CELLULITIS IN A PATIENT WITH SYSTEMIC LUPUS ERYTHEMATOSUS. *J Formos Med Assoc* 2002; **101**: 4.

408 Zaugg M, Salfinger M, Opravil M, Lüthy R. Extrapulmonary and disseminated infections due to Mycobacterium malmoense: case report and review. *Clin Infect Dis* 1993; **16**: 540–9.

409 Lahey T. Invasive Mycobacterium marinum Infections. *Emerg Infect Dis* 2003; **9**: 1496–8.

410 Nagao K, Ota T, Tanikawa A, *et al.* Genetic identification and detection of human pathogenic Rhizopus species, a major mucormycosis agent, by multiplex PCR based on internal transcribed spacer region of rRNA gene. *Journal of Dermatological Science* 2005; **39**: 23–31.

411 Wallace RJ, Brown-Elliott BA, Wilson RW, *et al.* Clinical and Laboratory Features of Mycobacterium porcinum. *J Clin Microbiol* 2004; **42**: 5689–97.

412 Bercovier H, Vincent V. Mycobacterial infections in domestic and wild animals due to Mycobacterium marinum, M. fortuitum, M. chelonae, M. porcinum, M. farcinogenes, M. smegmatis, M. scrofulaceum, M. xenopi, M. kansasii, M. simiae and M. genavense. *Rev - Off Int Epizoot* 2001; **20**: 265–90.

413 Wallace RJ, Brown-Elliott BA, Brown J, *et al.* Polyphasic Characterization Reveals that the Human Pathogen Mycobacterium peregrinum Type II Belongs to the Bovine Pathogen Species Mycobacterium senegalense. *J Clin Microbiol* 2005; **43**: 5925–35.

414 Tsukamura M. Mycobacterium shimoidei s p . nov., nom. rev., a Lung Pathogen. 2018; : 3.

415 Galizzi N, Tortoli E, Gori A, Morini F, Lapadula G. A Case of Mild Pulmonary Disease Due to Mycobacterium shimoidei with a Favorable Outcome. *J Clin Microbiol* 2013; **51**: 3467–8.

416 Wallace RJ, Nash DR, Tsukamura M, Blacklock ZM, Silcox VA. Human disease due to Mycobacterium smegmatis. *J Infect Dis* 1988; **158**: 52–9.

417 Maloney JM, Gregg CR, Stephens DS, Manian FA, Rimland D. Infections caused by Mycobacterium szulgai in humans. *Rev Infect Dis* 1987; **9**: 1120–6.

418 Van der Werf TS, Van der Graaf WT, Tappero JW, Asiedu K. Mycobacterium ulcerans infection. *The Lancet* 1999; **354**: 1013–8.

419 Simor AE, Salit IE, Vellend H. The role of Mycobacterium xenopi in human disease. *Am Rev Respir Dis* 1984; **129**: 435–8.

420 Costrini NV, Kogan M. Lectin-Induced Inhibition of Nerve Growth Factor Binding by Receptors of Sympathetic Ganglia. *Journal of Neurochemistry* 1981; **36**: 1175–80.

421 Reyn CFV, Green PA, Mccormick D, *et al.* Dual skin testing with Mycobacterium avium sensitin and purified protein derivative to discriminate pulmonary disease due to M. avium complex from pulmonary disease due to Mycobacterium tuberculosis. *J Infect Dis* 1998.

422 Inderlied CB, Kemper CA, Bermudez LE. The Mycobacterium avium complex. *Clin Microbiol Rev* 1993; **6**: 266–310.

423 Masur H. Recommendations on Prophylaxis and Therapy for Disseminated Mycobacterium avium Complex Disease in Patients Infected with the Human Immunodeficiency Virus. *New England Journal of Medicine* 1993; **329**: 898–904.

424 Naranjo V, Gortazar C, Vicente J, de la Fuente J. Evidence of the role of European wild boar as a reservoir of Mycobacterium tuberculosis complex. *Veterinary Microbiology* 2008; **127**: 1–9.

425 Rodrigue DC, Tauxe RV, Rowe B. International increase in Salmonella enteritidis: a new pandemic? *Epidemiol Infect* 1990; **105**: 21–7.

426 Fine PE. Leprosy: the epidemiology of a slow bacterium. *Epidemiol Rev* 1982; **4**: 161–88.

427 Suzuki K, Akama T, Kawashima A, Yoshihara A, Yotsu RR, Ishii N. Current status of leprosy: epidemiology, basic science and clinical perspectives. *J Dermatol* 2012; **39**: 121–9.

428 Smith NH, Dale J, Inwald J, *et al.* The population structure of Mycobacterium bovis in Great Britain: Clonal expansion. *Proc Natl Acad Sci U S A* 2003; **100**: 15271–5.

429 Michalak K, Austin C, Diesel S, Bacon MJ, Zimmerman P, Maslow JN. Mycobacterium tuberculosis infection as a zoonotic disease: transmission between humans and elephants. *Emerging Infect Dis* 1998; **4**: 283–7.

430 Deighton FC, Mulder JL. Mycocentrospora acerina as a human pathogen. *Transactions of the British Mycological Society* 1977; **69**: 326–7.

431 Waites KB, Talkington DF. Mycoplasma pneumoniae and its role as a human pathogen. *Clin Microbiol Rev* 2004; **17**: 697–728, table of contents.

432 Visvesvara GS, Stehr-Green JK. Epidemiology of Free-Living Ameba Infections [1]. *The Journal of Protozoology* 1990; **37**: 25s–33s.

433 Whitehouse CA. Crimean-Congo hemorrhagic fever. *Antiviral Res* 2004; **64**: 145–60.

434 Ajello L. Natural history of the dermatophytes and related fungi. *Mycopathologia et Mycologia Applicata* 1974; **53**: 93–110.

435 Sigler L, Summerbell RC, Poole L, *et al.* Invasive Nattrassia mangiferae infections: case report, literature review, and therapeutic and taxonomic appraisal. *J Clin Microbiol* 1997; **35**: 433–40.

436 Sarafian SK, Knapp JS. Molecular epidemiology of gonorrhea. *Clin Microbiol Rev* 1989; **2**: S49–55.

437 Cohn AC, MacNeil JR, Harrison LH, *et al.* Changes in Neisseria meningitidis Disease Epidemiology in the United States, 1998–2007: Implications for Prevention of Meningococcal Disease. *Clin Infect Dis* 2010; **50**: 184–91.

438 Stephens DS, Greenwood B, Br P, *et al.* (Prof P Brandtzaeg MD). Correspondence to: .

439 SWAIN CL, MARTIN DR. Survival of meningococci outside of the host: implications for acquisition. *Epidemiol Infect* 2007; **135**: 315–20.

440 Doern GV, Blacklow NR, Gantz NM, Aucoin P, Fischer RA, Parker DS. Neisseria sicca osteomyelitis. *J Clin Microbiol* 1982; **16**: 595–7.

441 Richards MR, Lowary TL. Chemistry and biology of galactofuranose-containing polysaccharides. *Chembiochem* 2009; **10**: 1920–38.

442 Satterwhite TK, Wallace RJ. Primary Cutaneous Nocardiosis. *JAMA* 1979; **242**: 333–6.

443 Smego RA, Gallis HA. The Clinical Spectrum of Nocardia brasiliensis Infection in the United States. *Rev Infect Dis* 1984; **6**: 164–80.

444 Torres OH, Domingo P, Pericas R, Boiron P, Montiel JA, Vázquez G. Infection caused by Nocardia farcinica: case report and review. *Eur J Clin Microbiol Infect Dis* 2000; **19**: 205–12.

445 Didier ES, Stovall ME, Green LC, Brindley PJ, Sestak K, Didier PJ. Epidemiology of microsporidiosis: sources and modes of transmission. *Vet Parasitol* 2004; **126**: 145–66.

446 Bethony J, Brooker S, Albonico M, *et al.* Soil-transmitted helminth infections: ascariasis, trichuriasis, and hookworm. *The Lancet* 2006; **367**: 1521–32.

447 Ghai RR, Chapman CA, Omeja PA, Davies TJ, Goldberg TL. Nodule Worm Infection in Humans and Wild Primates in Uganda: Cryptic Species in a Newly Identified Region of Human Transmission. *PLoS Negl Trop Dis* 2014; **8**. DOI:10.1371/journal.pntd.0002641.

448 Gasser RB, Woods WG, Huffman MA, Blotkamp J, Polderman AM. Molecular separation of Oesophagostomum stephanostomum and Oesophagostomum bifurcum (Nematoda: Strongyloidea) from non-human primates1Note: Nucleotide sequences reported in this paper are available in the GenBank™ database under the accession numbers AF136575 and AF136576.1. *International Journal for Parasitology* 1999; **29**: 1087–91.

449 Novakova A. Interesting and rare saprotrophic microfungi isolated from excrements and other substrata in the Domica and Ardovská Caves (Slovak Karst National Park, Slovakia). *ResearchGate* 2005; published online April. https://www.researchgate.net/publication/277015342_Interesting_and_rare_saprotrophic_microfungi_isolated_from_excrements_and_other_substrata_in_the_Domica_and_Ardovska_Caves_Slovak_Karst_National_Park_Slovakia/citations (accessed March 24, 2020).

450 Osei-Atweneboana MY, Eng JK, Boakye DA, Gyapong JO, Prichard RK. Prevalence and intensity of Onchocerca volvulus infection and efficacy of ivermectin in endemic communities in Ghana: a two-phase epidemiological study. *Lancet* 2007; **369**: 2021–9.

451 Schiller JT, Lowy DR. Vaccines to prevent infections by oncoviruses. *Annu Rev Microbiol* 2010; **64**: 23–41.

452 Sithithaworn P, Haswell-Elkins M. Epidemiology of Opisthorchis viverrini. *Acta Trop* 2003; **88**: 187–94.

453 Reeves WC, Hammon WM. California encephalitis virus, a newly described agent. III. Mosquito infection and transmission. *J Immunol* 1952; **69**: 511–4.

454 Gauci PJ, McAllister J, Mitchell IR, Weir RP, Melville LF, Gubala AJ. Genomic characterisation of Trubanaman and Gan Gan viruses, two bunyaviruses with potential significance to public health in Australia. *Virology Reports* 2016; **6**: 1–10.

455 Haddow AD, Odoi A. The incidence risk, clustering, and clinical presentation of La Crosse virus infections in the eastern United States, 2003-2007. *PLoS ONE* 2009; **4**: e6145.

456 Carvalho MGC, Frugulhetti IC, Rebello MA. Marituba (Bunyaviridae) virus replication in culturedAedes albopictus cells and in L-A9 cells. *Archives of Virology* 1986; **90**: 325–35.

457 Gomes MLC, Freitas RB, Travassos da Rosa JFS, *et al.* Oropouche Virus: I. A Review of Clinical, Epidemiological, and Ecological Findings*. *The American Journal of Tropical Medicine and Hygiene* 1981; **30**: 149–60.

458 Fernando R, Capone D, Elrich S, *et al.* Infection with New York Orthohantavirus and Associated Respiratory Failure and Multiple Cerebral Complications. *Emerg Infect Dis* 2019; **25**: 1241–3.

459 Yuen M-F, Chen D-S, Dusheiko GM, *et al.* Hepatitis B virus infection. *Nat Rev Dis Primers* 2018; **4**: 18035.

460 Shepard CW, Finelli L, Alter MJ. Global epidemiology of hepatitis C virus infection. *Lancet Infect Dis* 2005; **5**: 558–67.

461 Zhou H, Ma Z, Hu T, *et al.* Tamdy Virus in *Ixodid* Ticks Infesting Bactrian Camels, Xinjiang, China, 2018. *Emerg Infect Dis* 2019; **25**: 2136–8.

462 Walsh EE, Hall CB. Respiratory Syncytial Virus (RSV). In: Mandell, Douglas, and Bennett's Principles and Practice of Infectious Diseases. Elsevier, 2015: 1948-1960.e3.

463 Vorou RM, Papavassiliou VG, Pierroutsakos IN. Cowpox virus infection: an emerging health threat. *Curr Opin Infect Dis* 2008; **21**: 153–6.

464 Arita I, Jezek Z, Khodakevich L, Ruti K. Human Monkeypox: A Newly Emerged Orthopoxvirus Zoonosis in the Tropical Rain Forests of Africa. *The American Journal of Tropical Medicine and Hygiene* 1985; **34**: 781–9.

465 Lee BL, Grossniklaus HE, Capone A, Padhye AA, Sekhon AS. Ovadendron sulphureo-ochraceum Endophthalmitis After Cataract Surgery. *American Journal of Ophthalmology* 1995; **119**: 307–12.

466 Möller C, Dreyfuss MM. Microfungi from Antarctic Lichens, Mosses and Vascular Plants. *Mycologia* 1996; **88**: 922–33.

467 Piccini C, D'Alessandro B, Antúnez K, Zunino P. [No title found]. *World Journal of Microbiology and Biotechnology* 2002; **18**: 761–5.

468 Walboomers JM, Jacobs MV, Manos MM, *et al.* Human papillomavirus is a necessary cause of invasive cervical cancer worldwide. *J Pathol* 1999; **189**: 12–9.

469 Brummer E, Castaneda E, Restrepo A. Paracoccidioidomycosis: an update. *Clin Microbiol Rev* 1993; **6**: 89–117.

470 Aka NA, Adoubryn K, Rondelaud D, Dreyfuss G. Human paragonimiasis in Africa. *Ann Afr Med* 2008; **7**: 153–62.

471 Little MD. Paragonimus caliensis sp. n. and paragonimiasis in Colombia. *J Parasitol* 1968; **54**: 738–46.

472 Dekumyoy P, Waikagul J, Eom KS. Human lung fluke Paragonimus heterotremus: differential diagnosis between Paragonimus heterotremus and Paragonimus westermani infections by EITB. *Trop Med Int Health* 1998; **3**: 52–6.

473 Huei-lan C, Chih-piao H, Lien-yin H, *et al.* Studies on a new pathogenic lung fluke--Paragonimus hueit'ungensis sp. nov. *Chin Med J* 1977; **3**: 379–94.

474 Johannesen E, Nguyen V. *Paragonimus kellicotti* : A Lung Infection in Our Own Backyard. *Case Reports in Pathology* 2016; **2016**: 1–3.

475 Vargas-Arzola J, Segura-Salvador A, Reyes-Velasco L, *et al.* Detection of Paragonimus mexicanus (Trematoda) metacercariae in crabs from Oaxaca, Mexico. *Acta Tropica* 2014; **137**: 95–8.

476 Blair null, Agatsuma null, Watanobe null. Molecular evidence for the synonymy of three species of paragonimus, P. ohirai miyazaki, 1939, P. iloktsuenensis chen, 1940 and P. sadoensis miyazaki et al., 1968. *J Helminthol* 1997; **71**: 305–10.

477 Sugiyama H, Morishima Y, Kameoka Y, Arakawa K, Kawanaka M. Paragonimus ohirai metacercariae in crabs collected along the Arakawa River in Tokyo, Japan. *J Vet Med Sci* 2004; **66**: 927–31.

478 Cabrera BD. Paragonimiasis in the Philippines: current status. *Arzneimittelforschung* 1984; **34**: 1188–92.

479 Agatsuma T, Habe S. Genetic variability and differentiation of natural populations in three Japanese lung flukes, Paragonimus ohirai, Paragonimus iloktsuenensis and Paragonimus sadoensis (Digenea: Troglotrematidae). *J Parasitol* 1986; **72**: 417–33.

480 Yaemput S, Dekumyoy P, Visiassuk K. The natural first intermediate host of Paragonimus siamensis (Miyazaki and Wykoff, 1965) in Thailand. *Southeast Asian J Trop Med Public Health* 1994; **25**: 284–90.

481 Blair D, Zhengshan C, Minggang C, *et al.* Paragonimus skrjabini Chen, 1959 (Digenea: Paragonimidae) and related species in eastern Asia: a combined molecular and morphological approach to identification and taxonomy. *Syst Parasitol* 2005; **60**: 1–21.

482 Ochigbo SO, Ekanem EE, Udo JJ. Prevalence and intensity of Paragonimus uterobilateralis infection among school children in Oban village, South Eastern, Nigeria. *Trop Doct* 2007; **37**: 224–6.

483 Kalhan S, Sharma P, Sharma S, Kakria N, Dudani S, Gupta A. Paragonimus westermani infection in lung: A confounding diagnostic entity. *Lung India* 2015; **32**: 265–7.

484 Miyazaki I, Vajrasthira S. ON A NEW LUNG FLUKE, PARAGONIMUS BANGKOKENSIS sp. nov. IN THAILAND (TRAMATODA: TROGLOTREMATIDAE). *JJMSB* 1967; **20**: 243–9.

485 Groves RW, Wilson-Jones E, MacDonald DM. Human orf and milkers' nodule: a clinicopathologic study. *J Am Acad Dermatol* 1991; **25**: 706–11.

486 Werchniak AE, Herfort OP, Farrell TJ, Connolly KS, Baughman RD. Milker's nodule in a healthy young woman. *J Am Acad Dermatol* 2003; **49**: 910–1.

487 Leavell UW, Phillips IA. Milker's nodules. Pathogenesis, tissue culture, electron microscopy, and calf inoculation. *Arch Dermatol* 1975; **111**: 1307–11.

488 Heegaard ED, Brown KE. Human Parvovirus B19. *Clinical Microbiology Reviews* 2002; **15**: 485–505.

489 Escande F, Lion C. Epidemiology of human infections by Pasteurella and related groups in France. *Zentralblatt für Bakteriologie* 1993; **279**: 131–9.

490 Kainz A, Lubitz W, Busse H-J. Genomic Fingerprints, ARDRA Profiles and Quinone Systems for Classification of Pasteurella sensu stricto. *Systematic and Applied Microbiology* 2000; **23**: 494–503.

491 Allison K, Clarridge JE. Long-Term Respiratory Tract Infection with Canine-Associated Pasteurella dagmatis and Neisseria canis in a Patient with Chronic Bronchiectasis. *Journal of Clinical Microbiology* 2005; **43**: 4272–4.

492 Callegari D, Kramer L, Cantoni AM, Di Lecce R, Dodi PL, Grandi G. Canine bladderworm (Capillaria plica) infection associated with glomerular amyloidosis. *Veterinary Parasitology* 2010; **168**: 338–41.

493 Reshetnyak VI, Karlovich TI, Ilchenko LU. Hepatitis G virus. *World J Gastroenterol* 2008; **14**: 4725–34.

494 Lyratzopoulos G, Ellis M, Nerringer R, Denning DW. Invasive Infection due to Penicillium Species other than P. marneffei. *Journal of Infection* 2002; **45**: 184–95.

495 Engleberg NC, Johnson J, Bluestein J, Madden K, Rinaldi MG. Phaeohyphomycotic cyst caused by a recently described species, Phaeoannellomyces elegans. *J Clin Microbiol* 1987; **25**: 605–8.

496 Scott RS, Sutton DA, Jagirdar J. Lung infection due to opportunistic fungus, Phialemonium obovatum, in a bone marrow transplant recipient: an emerging infection with fungemia and Crohn disease-like involvement of the gastrointestinal tract. *Ann Diagn Pathol* 2005; **9**: 227–30.

497 Lomax LG, Cole JR, Padhye AA, Ajello L, Chandler FW, Smith BR. Osteolytic phaeohyphomycosis in a German shepherd dog caused by Phialemonium obovatum. *J Clin Microbiol* 1986; **23**: 987–91.

498 Medlar EM. A New Fungus, Phialophora verrucosa, Pathogenic for Man. *Mycologia* 1915; **7**: 200–3.

499 Bird BH, Ksiazek TG, Nichol ST, Maclachlan NJ. Rift Valley fever virus. *J Am Vet Med Assoc* 2009; **234**: 883–93.

500 Dionisio D, Esperti F, Vivarelli A, Valassina M. Epidemiological, clinical and laboratory aspects of sandfly fever. *Curr Opin Infect Dis* 2003; **16**: 383–8.

501 Eisa M. Preliminary survey of domestic animals of the Sudan for precipitating antibodies to Rift Valley fever virus. *J Hyg (Lond)* 1984; **93**: 629–37.

502 Zaitz C, Heins-Vaccari EM, Freitas RS de, *et al.* SUBCUTANEOUS PHEOHYPHOMYCOSIS CAUSED BY Phoma cava: REPORT OF A CASE AND REVIEW OF THE LITERATURE. *Rev Inst Med trop S Paulo* 1997; **39**: 43–8.

503 Bonifaz A, Gómez-Daza F, Paredes V, Ponce RM. Tinea versicolor, tinea nigra, white piedra, and black piedra. *Clinics in Dermatology* 2010; **28**: 140–5.

504 Gajadhar AA. Foodborne Parasites in the Food Supply Web: Occurrence and Control. Woodhead Publishing, 2015.

505 Coulson RMR, Hall N, Ouzounis CA. Comparative genomics of transcriptional control in the human malaria parasite Plasmodium falciparum. *Genome Res* 2004; **14**: 1548–54.

506 Mueller I, Zimmerman PA, Reeder JC. Plasmodium malariae and Plasmodium ovale--the 'bashful' malaria parasites. *Trends Parasitol* 2007; **23**: 278–83.

507 Collins WE, Jeffery GM. Plasmodium ovale: Parasite and Disease. *Clin Microbiol Rev* 2005; **18**: 570–81.

508 Brasil P, Zalis MG, de Pina-Costa A, *et al.* Outbreak of human malaria caused by Plasmodium simium in the Atlantic Forest in Rio de Janeiro: a molecular epidemiological investigation. *The Lancet Global Health* 2017; **5**: e1038–46.

509 Marchesini P, Carter R, Mendis K, Sina B. The neglected burden of Plasmodium vivax malaria. *The American Journal of Tropical Medicine and Hygiene* 2001; **64**: 97–106.

510 Dumoulin A, Mazars E, Seguy N, *et al.* Transmission of Pneumocystis carinii disease from immunocompetent contacts of infected hosts to susceptible hosts. *Eur J Clin Microbiol Infect Dis* 2000; **19**: 671–8.

511 Tan CS, Koralnik IJ. Progressive multifocal leukoencephalopathy and other disorders caused by JC virus: clinical features and pathogenesis. *Lancet Neurol* 2010; **9**: 425–37.

512 Weber T. Progressive multifocal leukoencephalopathy. *Neurol Clin* 2008; **26**: 833–54, x–xi.

513 WILLEMS A, COLLINS MD. Reclassification of Oribaculum catoniae (Moore and Moore 1994) as Porphyromonas catoniae comb. nov. and Emendation of the Genus Porphyromonas. *International Journal of Systematic and Evolutionary Microbiology,* 1995; **45**: 578–81.

514 Moore LV, Johnson JL, Moore WE. Descriptions of Prevotella tannerae sp. nov. and Prevotella enoeca sp. nov. from the human gingival crevice and emendation of the description of Prevotella zoogleoformans. *Int J Syst Bacteriol* 1994; **44**: 599–602.

515 O'Flynn C, Deusch O, Darling AE, *et al.* Comparative Genomics of the Genus Porphyromonas Identifies Adaptations for Heme Synthesis within the Prevalent Canine Oral Species Porphyromonas cangingivalis. *Genome Biol Evol* 2015; **7**: 3397–413.

516 Walter MRV, Morck DW. In vitro expression of tumor necrosis factor-alpha, interleukin 1beta, and interleukin 8 mRNA by bovine macrophages following exposure to Porphyromonas levii. *Can J Vet Res* 2002; **66**: 93–8.

517 Hillier SL, Kiviat NB, Hawes SE, *et al.* Role of bacterial vaginosis–associated microorganisms in endometritis. *American Journal of Obstetrics and Gynecology* 1996; **175**: 435–41.

518 Talan DA, Abrahamian FM, Moran GJ, Citron DM, Tan JO, Goldstein EJC. Clinical Presentation and Bacteriologic Analysis of Infected Human Bites in Patients Presenting to Emergency Departments. *Clin Infect Dis* 2003; **37**: 1481–9.

519 Dewhirst FE, Chen T, Izard J, *et al.* The Human Oral Microbiome. *J Bacteriol* 2010; **192**: 5002–17.

520 Sikorska B, Liberski PP. Human Prion Diseases: From Kuru to Variant Creutzfeldt-Jakob Disease. In: Harris JR, ed. Protein Aggregation and Fibrillogenesis in Cerebral and Systemic Amyloid Disease. Dordrecht: Springer Netherlands, 2012: 457–96.

521 Kamin M, M. Patten B. Creutzfeldt-Jakob disease: Possible transmission to humans by consumption of wild animal brains. *The American Journal of Medicine* 1984; **76**: 142–5.

522 Bowden GHW. Actinomyces, Propionibacterium propionicus, and Streptomyces. In: Baron S, ed. Medical Microbiology, 4th edn. Galveston (TX): University of Texas Medical Branch at Galveston, 1996. http://www.ncbi.nlm.nih.gov/books/NBK8385/ (accessed March 24, 2020).

523 Valour F, Ferry T, Karsenty J, *et al.* Actinomycosis: etiology, clinical features, diagnosis, treatment, and management. *Infection and Drug Resistance* 2014; : 183.

524 Pignato S, Giammanco GM, Grimont F, Grimont PA, Giammanco G. Molecular characterization of the genera Proteus, Morganella, and Providencia by ribotyping. *J Clin Microbiol* 1999; **37**: 2840–7.

525 Falagas ME, Kavvadia PK, Mantadakis E, *et al.* Morganella morganii infections in a general tertiary hospital. *Infection* 2006; **34**: 315–21.

526 Bernstein EF, Schuster MG, Stieritz DD, Heuman PC, Uitto J. Disseminated cutaneous Pseudallescheria boydii. *Br J Dermatol* 1995; **132**: 456–60.

527 Sutton DA, Timm WD, Morgan-Jones G, Rinaldi MG. Human Phaeohyphomycotic Osteomyelitis Caused by the Coelomycete Phomopsis Saccardo 1905: Criteria for Identification, Case History, and Therapy. *J Clin Microbiol* 1999; **37**: 807–11.

528 Bodey GP, Bolivar R, Fainstein V, Jadeja L. Infections Caused by Pseudomonas aeruginosa. *Rev Infect Dis* 1983; **5**: 279–313.

529 Noble RC, Overman SB. Pseudomonas stutzeri infection a review of hospital isolates and a review of the literature. *Diagnostic Microbiology and Infectious Disease* 1994; **19**: 51–6.

530 Rollinson D. Advances in parasitology. Volume eighty two Volume eighty two. Amsterdam; Oxford: Elsevier ; Academic Press, 2013 http://public.ebookcentral.proquest.com/choice/publicfullrecord.aspx?p=1172781 (accessed March 24, 2020).

531 Khan SU, Gurley ES, Hossain MJ, Nahar N, Sharker MAY, Luby SP. A Randomized Controlled Trial of Interventions to Impede Date Palm Sap Contamination by Bats to Prevent Nipah Virus Transmission in Bangladesh. *PLoS One* 2012; **7**. DOI:10.1371/journal.pone.0042689.

532 Thiyagarajan M, Bagul A, Nicholson ML. A nodulo-cystic eumycetoma caused by Pyrenochaeta romeroi in a renal transplant recipient: A case report. *J Med Case Reports* 2011; **5**: 460.

533 Badali H, Bonifaz A, Barrón-Tapia T, *et al. Rhinocladiella aquaspersa* , proven agent of verrucous skin infection and a novel type of chromoblastomycosis. *Med Mycol* 2010; **48**: 696–703.

534 Del Palacio-Hernanz A, Moore MK, Campbell CK, Del Palacio-Perez-Medel A, Del Castillo-Cantero R. Infection of the central nervous system by *Rhinocladiella atrovirens* in a patient with acquired immunodeficiency syndrome. *Med Mycol* 1989; **27**: 127–30.

535 Berman JJ. Taxonomic guide to infectious diseases: understanding the biologic classes of pathogenic organisms, 1st ed. London ; Waltham, MA: Elsevier/Academic Press, 2012.

536 Carrion A, Silva M. Chromoblastomycosis and Its Etiologic Fungi. In: Biology of Pathogenic Fungi. Waltham, Mass: Chronica Botanica Co, 1947: 20–62.

537 Arseculeratne SN. Recent advances in rhinosporidiosis and Rhinosporidium seeberi. *Indian J Med Microbiol* 2002; **20**: 119–31.

538 Espinel-Ingroff A, Oakley LA, Kerkering TM. Opportunistic zygomycotic infections. *Mycopathologia* 1987; **97**: 33–41.

539 Richardson M. The ecology of the Zygomycetes and its impact on environmental exposure. *Clin Microbiol Infect* 2009; **15 Suppl 5**: 2–9.

540 Severo LC, Job F, Mattos TC. Systemic zygomycosis: nosocomial infection by Rhizomucor pusillus. *Mycopathologia* 1991; **113**: 79–80.

541 Schipper MAA, Maslen MM, Hogg GG, Chow CW, Samson RA. Human infection by *Rhizopus azygosporus* and the occurrence of azygospores in Zygomycetes. *Med Mycol* 1996; **34**: 199–203.

542 Cheng VCC, Chan JFW, Ngan AHY, *et al.* Outbreak of intestinal infection due to Rhizopus microsporus. *J Clin Microbiol* 2009; **47**: 2834–43.

543 Ibrahim AS, Spellberg B, Avanessian V, Fu Y, Edwards JE. Rhizopus oryzae adheres to, is phagocytosed by, and damages endothelial cells in vitro. *Infect Immun* 2005; **73**: 778–83.

544 Bauer H, Ajello L, Adams E, Hernandez DU. Cerebral mucormycosis: Pathogenesis of the disease. *The American Journal of Medicine* 1955; **18**: 822–31.

545 Kaufman DD, Blake J. Degradation of atrazine by soil fungi. *Soil Biology and Biochemistry* 1970; **2**: 73–80.

546 Sugar D. Pear storage decay control through tree management and post-harvest treatment. Yakima, Washington: Washington State Horticultural Association, 1992.

547 Kelly PJ, Beati L, Mason PR, Matthewman LA, Roux V, Raoult D. Rickettsia africae sp. nov., the etiological agent of African tick bite fever. *Int J Syst Bacteriol* 1996; **46**: 611–4.

548 Parola P, Vestris G, Martinez D, Brochier B, Roux V, Raoult D. Tick-borne rickettiosis in Guadeloupe, the French West Indies: isolation of Rickettsia africae from Amblyomma variegatum ticks and serosurvey in humans, cattle, and goats. *Am J Trop Med Hyg* 1999; **60**: 888–93.

549 Paddock CD, Koss T, Eremeeva ME, Dasch GA, Zaki SR, Sumner JW. Isolation of Rickettsia akari from eschars of patients with rickettsialpox. *Am J Trop Med Hyg* 2006; **75**: 732–8.

550 Ogata H, Audic S, Renesto-Audiffren P, *et al.* Mechanisms of evolution in Rickettsia conorii and R. prowazekii. *Science* 2001; **293**: 2093–8.

551 Rovery C, Brouqui P, Raoult D. Questions on Mediterranean Spotted Fever a Century after Its Discovery. *Emerg Infect Dis* 2008; **14**: 1360–7.

552 Fang R, Houhamdi L, Raoult D. Detection of Rickettsia prowazekii in Body Lice and Their Feces by Using Monoclonal Antibodies. *Journal of Clinical Microbiology* 2002; **40**: 3358–63.

553 Walker DH, Ismail N. Emerging and re-emerging rickettsioses: endothelial cell infection and early disease events. *Nat Rev Microbiol* 2008; **6**: 375–86.

554 Kenney M, Eveland LK, Yermakov V, Kassouny DY. A case of Rictularia infection of man in New York. *Am J Trop Med Hyg* 1975; **24**: 596–9.

555 Lindquist WD, Li SY. Some nematodes of rats from Guam, M.I. and notes on a species of Reticularia. *J Parasitol* 1955; **41**: 194–7.

556 Yue MY, Jensen JM, Jordan HE, Jue. Spirurid Infections (Rictularia sp.) in Golden Marmosets (A), Leontopithecus Rosalia (syn. Leontideus Rosalia) from the Oklahoma City Zoo. *The Journal of Zoo Animal Medicine* 1980; **11**: 77.

557 Prober C. Sixth Disease and the Ubiquity of Human Herpesviruses. *N Engl J Med* 2005; **352**: 753–5.

558 Estes MK, Palmer EL, Obijeski JF. Rotaviruses: a review. *Curr Top Microbiol Immunol* 1983; **105**: 123–84.

559 Leung AKC, Kellner JD, Dele Davies H. Rotavirus gastroenteritis. *Adv Therapy* 2005; **22**: 476–87.

560 Gerba CP, Rose JB, Haas CN, Crabtree KD. Waterborne rotavirus: A risk assessment. *Water Research* 1996; **30**: 2929–40.

561 Frey TK. Molecular biology of rubella virus. *Adv Virus Res* 1994; **44**: 69–160.

562 Lambert N, Strebel P, Orenstein W, Icenogle J, Poland GA. Rubella. *Lancet* 2015; **385**: 2297–307.

563 Barr JA, Smith C, Marsh GA, Field H, Wang L-F. Evidence of bat origin for Menangle virus, a zoonotic paramyxovirus first isolated from diseased pigs. *Journal of General Virology* 2012; **93**: 2590–4.

564 Bowden TR, Westenberg M, Wang L-F, Eaton BT, Boyle DB. Molecular Characterization of Menangle Virus, a Novel Paramyxovirus which Infects Pigs, Fruit Bats, and Humans. *Virology* 2001; **283**: 358–73.

565 Kirkland P, Loveb R, Philbey A, Ross A, Davis R, Hart K. Epidemiology and control of Menangle virus in pigs. *Australian Veterinary Journal* 2001; **79**: 199–206.

566 Galazka AM, Robertson SE, Kraigher A. Mumps and mumps vaccine: a global review. *Bull World Health Organ* 1999; **77**: 3–14.

567 Greenberger PA. Hypersensitivity pneumonitis: A fibrosing alveolitis produced by inhalation of diverse antigens. *Journal of Allergy and Clinical Immunology* 2019; **143**: 1295–301.

568 Baradkar VP, Kumar S. Cutaneous zygomycosis due to Saksenaea vasiformis in an immunocompetent host. *Indian J Dermatol* 2009; **54**: 382–4.

569 Lawley TD, Bouley DM, Hoy YE, Gerke C, Relman DA, Monack DM. Host transmission of Salmonella enterica serovar Typhimurium is controlled by virulence factors and indigenous intestinal microbiota. *Infect Immun* 2008; **76**: 403–16.

570 Davies PR, Morrow WE, Jones FT, Deen J, Fedorka-Cray PJ, Harris IT. Prevalence of Salmonella in finishing swine raised in different production systems in North Carolina, USA. *Epidemiol Infect* 1997; **119**: 237–44.

571 Kidgell C, Reichard U, Wain J, *et al.* Salmonella typhi, the causative agent of typhoid fever, is approximately 50,000 years old. *Infect Genet Evol* 2002; **2**: 39–45.

572 Nauciel C, Espinasse-Maes F. Role of gamma interferon and tumor necrosis factor alpha in resistance to Salmonella typhimurium infection. *Infect Immun* 1992; **60**: 450–4.

573 Yu H, Bruno JG. Immunomagnetic-electrochemiluminescent detection of Escherichia coli O157 and Salmonella typhimurium in foods and environmental water samples. *Appl Environ Microbiol* 1996; **62**: 587–92.

574 Fayer R. Sarcocystis spp. in human infections. *Clin Microbiol Rev* 2004; **17**: 894–902, table of contents.

575 Latif B, Heo CC, Razuin R, Shamalaa DV, Tappe D. Autochthonous human schistosomiasis, Malaysia. *Emerging Infect Dis* 2013; **19**: 1340–1.

576 Jourdane J, Southgate VR, Pagès JR, Durand P, Tchuem Tchuenté LA. Recent studies on Schistosoma intercalatum: taxonomic status, puzzling distribution and transmission foci revisited. *Mem Inst Oswaldo Cruz* 2001; **96 Suppl**: 45–8.

577 Carlton EJ, Hsiang M, Zhang Y, Johnson S, Hubbard A, Spear RC. The impact of Schistosoma japonicum infection and treatment on ultrasound-detectable morbidity: a five-year cohort study in Southwest China. *PLoS Negl Trop Dis* 2010; **4**: e685.

578 Wolmarans CT, de Kock KN, van der Walt MP. An experimental Schistosoma mattheei infection in man. *Onderstepoort J Vet Res* 1990; **57**: 211–4.

579 Muth S, Sayasone S, Odermatt-Biays S, Phompida S, Duong S, Odermatt P. Schistosoma mekongi in Cambodia and Lao People's Democratic Republic. *Adv Parasitol* 2010; **72**: 179–203.

580 Horák P, Mikeš L, Lichtenbergová L, Skála V, Soldánová M, Brant SV. Avian Schistosomes and Outbreaks of Cercarial Dermatitis. *Clin Microbiol Rev* 2015; **28**: 165–90.

581 Samerpitak K, Van der Linde E, Choi H-J, *et al.* Taxonomy of Ochroconis, genus including opportunistic pathogens on humans and animals. *Fungal Diversity* 2014; **65**: 89–126.

582 Giraldo A, Sutton DA, Gené J, Fothergill AW, Cano J, Guarro J. Rare Arthroconidial Fungi in Clinical Samples: Scytalidium cuboideum and Arthropsis hispanica. *Mycopathologia* 2013; **175**: 115–21.

583 Bisiaux-Salauze B, Perez C, Sebald M, Petit JC. Bacteremias caused by Selenomonas artemidis and Selenomonas infelix. *J Clin Microbiol* 1990; **28**: 140–2.

584 Cruz P, Mehretu AM, Buttner MP, Trice T, Howard KM. Development of a polymerase chain reaction assay for the rapid detection of the oral pathogenic bacterium, Selenomonas noxia. *BMC Oral Health* 2015; **15**: 95.

585 Sekhsokh Y, Arsalane L, El Ouenass M, Doublali T, Bajjou T, Lahlou Amine I. Bactériémie à Serratia rubidaea. *Médecine et Maladies Infectieuses* 2007; **37**: 287–9.

586 Niyogi SK. Shigellosis. *J Microbiol* 2005; **43**: 133–43.

587 Legros D. Shigellosis: Report of a Workshop: held at ICDDR,B: Centre for Health and Population Research, Dhaka, Bangladesh, on 16-18 February 2004. *Journal of Health, Population and Nutrition* 2004; **22**: 445–9.

588 Whitley RJ. Herpesviruses. In: Baron S, ed. Medical Microbiology, 4th edn. Galveston (TX): University of Texas Medical Branch at Galveston, 1996. http://www.ncbi.nlm.nih.gov/books/NBK8157/ (accessed March 17, 2020).

589 Wald A, Corey L. Persistence in the population: epidemiology, transmission. In: Arvin A, Campadelli-Fiume G, Mocarski E, *et al.*, eds. Human Herpesviruses: Biology, Therapy, and Immunoprophylaxis. Cambridge: Cambridge University Press, 2007. http://www.ncbi.nlm.nih.gov/books/NBK47447/ (accessed March 17, 2020).

590 Lapson C. Rat-Bite Fever in Washington DC, Due to Spirilum Minus and Streptobacillus Moniliformis. CDC, 1941 https://stacks.cdc.gov/view/cdc/70079/cdc_70079_DS1.pdf.

591 Gaastra W, Boot R, Ho HTK, Lipman LJA. Rat bite fever. *Vet Microbiol* 2009; **133**: 211–28.

592 Kauffman CA, Bustamante B, Chapman SW, Pappas PG. Clinical Practice Guidelines for the Management of Sporotrichosis: 2007 Update by the Infectious Diseases Society of America. *Clin Infect Dis* 2007; **45**: 1255–65.

593 Archer GL. Staphylococcus aureus: a well-armed pathogen. *Clin Infect Dis* 1998; **26**: 1179–81.

594 Kanuparthy A, Challa T, Meegada S, Siddamreddy S, Muppidi V. Staphylococcus warneri: Skin Commensal and a Rare Cause of Urinary Tract Infection. *Cureus* 2020; published online May 28. DOI:10.7759/cureus.8337.

595 Brooke JS. Stenotrophomonas maltophilia: an emerging global opportunistic pathogen. *Clin Microbiol Rev* 2012; **25**: 2–41.

596 Elliott SP. Rat bite fever and Streptobacillus moniliformis. *Clin Microbiol Rev* 2007; **20**: 13–22.

597 Johansson L, Thulin P, Low DE, Norrby-Teglund A. Getting under the skin: the immunopathogenesis of Streptococcus pyogenes deep tissue infections. *Clin Infect Dis* 2010; **51**: 58–65.

598 Klein RS, Catalano MT, Edberg SC, Casey JI, Steigbigel NH. Streptococcus bovis septicemia and carcinoma of the colon. *Ann Intern Med* 1979; **91**: 560–2.

599 Clarridge JE, Attorri S, Musher DM, Hebert J, Dunbar S. Streptococcus intermedius, Streptococcus constellatus, and Streptococcus anginosus ('Streptococcus milleri Group') Are of Different Clinical Importance and Are Not Equally Associated with Abscess. *Clinical Infectious Diseases* 2001; **32**: 1511–5.

600 Weese JS, Jarlot C, Morley PS. Survival of Streptococcus equi on surfaces in an outdoor environment. *Can Vet J* 2009; **50**: 968–70.

601 Mosailova N, Truong J, Dietrich T, Ashurst J. *Streptococcus gordonii* : A Rare Cause of Infective Endocarditis. *Case Reports in Infectious Diseases* 2019; **2019**: 1–2.

602 Yombi J cyr, Belkhir L, Jonckheere S, *et al.* Streptococcus gordonii septic arthritis : two cases and review of literature. *BMC Infect Dis* 2012; **12**: 215.

603 Seipke RF, Kaltenpoth M, Hutchings MI. Streptomyces as symbionts: an emerging and widespread theme? *FEMS Microbiol Rev* 2012; **36**: 862–76.

604 Quintana ET, Wierzbicka K, Mackiewicz P, *et al.* Streptomyces sudanensis sp. nov., a new pathogen isolated from patients with actinomycetoma. *Antonie van Leeuwenhoek* 2008; **93**: 305–13.

605 Viney ME, Lok JB. The biology of Strongyloides spp. WormBook, 2018 http://www.ncbi.nlm.nih.gov/books/NBK19795/ (accessed March 16, 2020).

606 Réblová M, Seifert KA, Fournier J, Štěpánek V. Newly recognised lineages of perithecial ascomycetes: the new orders Conioscyphales and Pleurotheciales. *Persoonia* 2016; **37**: 57–81.

607 Costa PSG da, Mendes JM de C, Ribeiro GM. Tatumella ptyseos causing severe human infection: report of the first two Brazilian cases. *Braz J Infect Dis* 2008; **12**. DOI:10.1590/S1413-86702008000500017.

608 Markham WD, Key RD, Padhye AA, Ajello L. Phaeohyphomycotic cyst caused by Tetraploa aristata. *J Med Vet Mycol* 1990; **28**: 147–50.

609 Lvov DK, Karas FR, Tsyrkin YM, *et al.* Batken virus, a new arbovirus isolated from ticks and mosquitoes in Kirghiz S.S.R. *Arch Gesamte Virusforsch* 1974; **44**: 70–3.

610 Labuda M, Nuttall PA. Tick-borne viruses. *Parasitology* 2004; **129**: S221–45.

611 Magnaval JF, Glickman LT, Dorchies P, Morassin B. Highlights of human toxocariasis. *Korean J Parasitol* 2001; **39**: 1–11.

612 Tenter AM, Heckeroth AR, Weiss LM. Toxoplasma gondii: from animals to humans. *Int J Parasitol* 2000; **30**: 1217–58.

613 Stamm LV. Pinta: Latin America's Forgotten Disease? *Am J Trop Med Hyg* 2015; **93**: 901–3.

614 Aristone A. Syphilis: Etiology, Epidemiology, and Origin Theory. *Totem: The University of Western Ontario Journal of Anthropology* 2011; **3**. https://ir.lib.uwo.ca/totem/vol3/iss1/6.

615 Yao C, Prestwood AK, McGraw RA. Trichinella spiralis (T1) and Trichinella T5: a comparison using animal infectivity and molecular biology techniques. *J Parasitol* 1997; **83**: 88–95.

616 Chouaki T, Lavarde V, Lachaud L, Raccurt CP, Hennequin C. Invasive Infections Due to *Trichoderma* Species: Report of 2 Cases, Findings of In Vitro Susceptibility Testing, and Review of the Literature. *CLIN INFECT DIS* 2002; **35**: 1360–7.

617 Coleman JS, Gaydos CA, Witter F. Trichomonas vaginalis Vaginitis in Obstetrics and Gynecology Practice: New Concepts and Controversies. *Obstetrical & Gynecological Survey* 2013; **68**: 43–50.

618 Ames I. Dermatophytosis. *Inst Int Coop Anim Biol* 2013; **3**: 1–13.

619 Branscomb R. The Dermatophytoses. *Lab Med* 2005; **36**: 496–500.

620 Lamb SR, Rademaker M. Tinea due to Trichophyton violaceum and Trichophyton soudanense in Hamilton, New Zealand. *Australas J Dermatol* 2001; **42**: 260–3.

621 Stockdale PM, Mackenzie DW, Austwick PK. Arthroderma simii sp. nov., the perfect state of Trichophyton simii (Pinoy) comb. nov. *Sabouraudia* 1965; **4**: 112–23.

622 Saxena P, Kumar A, Shrivastava JN. Diversity of keratinophilic mycoflora in the soil of Agra (India). *Folia Microbiol (Praha)* 2004; **49**: 430–4.

623 Rippon JW, Eng A, Malkinson FD. Trichophyton simii Infection in the United States. *Arch Dermatol* 1968; **98**: 615–9.

624 Wolf DG, Falk R, Hacham M, *et al.* Multidrug-resistant Trichosporon asahii infection of nongranulocytopenic patients in three intensive care units. *J Clin Microbiol* 2001; **39**: 4420–5.

625 Passo CL, Pernice I, Celeste A, Perdichizzi G, Todaro-Luck F. Transmission of Trichosporon asahii oesophagitis by a contaminated endoscope. *Mycoses* 2001; **44**: 13–21.

626 Colombo AL, Padovan ACB, Chaves GM. Current knowledge of Trichosporon spp. and Trichosporonosis. *Clin Microbiol Rev* 2011; **24**: 682–700.

627 Erer B, Galimberti M, Lucarelli G, *et al.* Trichosporon beigelii : a life-threatening pathogen in immunocompromised hosts. *Bone Marrow Transplantation* 2000; **25**: 745–9.

628 Nakagawa T, Nakashima K, Takaiwa T, Negayama K. Trichosporon cutaneum (Trichosporon asahii) infection mimicking hand eczema in a patient with leukemia. *Journal of the American Academy of Dermatology* 2000; **42**: 929–31.

629 Callinan APL, Westcott JM. Vertical distribution of trichostrongylid larvae on herbage and in soil. *International Journal for Parasitology* 1986; **16**: 241–4.

630 Sevimli F. Checklist of small ruminant gastrointestinal nematodes and their geographical distribution in Turkey. *Turkish Journal of Veterinary and Animal Sciences* 2013; **37**: 369–79.

631 Bradbury R. An imported case of trichostrongylid infection in Tasmania and a review of human trichostrongylidiosis. *Annals of the ACTM: An International Journal of Tropical and Travel Medicine* 2006; **7**: 25.

632 Wall EC, Bhatnagar N, Watson J, Doherty T. An Unusual Case of Hypereosinophilia and Abdominal Pain: An Outbreak of Trichostrongylus Imported From New Zealand. *J Travel Med* 2011; **18**: 59–60.

633 McCarthy J, Moore TA. Emerging helminth zoonoses. *Int J Parasitol* 2000; **30**: 1351–60.

634 Brooker S, Clements ACA, Bundy D a. P. Global epidemiology, ecology and control of soil-transmitted helminth infections. *Adv Parasitol* 2006; **62**: 221–61.

635 Moraes RNR, Ribeiro MCT, Nogueira MCL, Cunha KC, Soares MMCN, Almeida MTG. First Report of Tritirachium oryzae Infection of Human Scalp. *Mycopathologia* 2010; **169**: 257–9.

636 Keating J, Yukich JO, Sutherland CS, Woods G, Tediosi F. Human African trypanosomiasis prevention, treatment and control costs: A systematic review. *Acta Tropica* 2015; **150**: 4–13.

637 Rassi A, Rassi A, Marin-Neto JA. Chagas heart disease: pathophysiologic mechanisms, prognostic factors and risk stratification. *Mem Inst Oswaldo Cruz* 2009; **104 Suppl 1**: 152–8.

638 Coura JR, Castro SL de. A Critical Review on Chagas Disease Chemotherapy. *Mem Inst Oswaldo Cruz* 2002; **97**: 3–24.

639 Safaei S, Fatahi-Bafghi M, Pouresmaeil O. Role of Tsukamurella species in human infections: first literature review. *New Microbes and New Infections* 2018; **22**: 6–12.

640 Arvin AM. Varicella-zoster virus. *Clin Microbiol Rev* 1996; **9**: 361–81.

641 Bergold GH, Munz K. Characterization of Piry virus. *Arch Gesamte Virusforsch* 1970; **31**: 152–67.

642 Tesh RB, Chaniotis BN, Johnson KM. Vesicular stomatitis virus (Indiana serotype): transovarial transmission by phlebotomine sandlies. *Science* 1972; **175**: 1477–9.

643 Reidl J, Klose KE. Vibrio cholerae and cholera: out of the water and into the host. *FEMS Microbiol Rev* 2002; **26**: 125–39.

644 Brayton PR, Bode RB, Colwell RR, *et al.* Vibrio cincinnatiensis sp. nov., a new human pathogen. *J Clin Microbiol* 1986; **23**: 104–8.

645 Yeung PSM, Boor KJ. Epidemiology, pathogenesis, and prevention of foodborne Vibrio parahaemolyticus infections. *Foodborne Pathog Dis* 2004; **1**: 74–88.

646 Strom MS, Paranjpye RN. Epidemiology and pathogenesis of Vibrio vulnificus. *Microbes Infect* 2000; **2**: 177–88.

647 Mishra SK, Ajello L, Ahearn DG, *et al.* Environmental mycology and its importance to public health. *Med Mycol* 1992; **30**: 287–305.

648 Edeson JFB, Wilson T. The Epidemiology of Filariasis Due to Wuchereria Bancrofti and Brugia Malayi. *Annu Rev Entomol* 1964; **9**: 245–68.

649 Downie AW, Taylor-Robinson CH, Caunt AE, Nelson GS, Manson-Bahr PEC, Matthews TCH. Tanapox: A New Disease Caused by a Pox Virus. *Br Med J* 1971; **1**: 363–8.

650 Cho CT, Wenner, Herbert A. Monkeypox Virus. *Bacteriological Reviews* 1973; **37**: 1–18.

651 Sabina Y, Rahman A, Ray RC, Montet D. Yersinia enterocolitica: Mode of Transmission, Molecular Insights of Virulence, and Pathogenesis of Infection. *J Pathog* 2011; **2011**: 429069.

652 Cafferkey MT, Sloane A, McCrae S, O'Morain CA. Yersinia frederiksenii infection and colonization in hospital staff. *Journal of Hospital Infection* 1993; **24**: 109–15.

653 Butler T, Islam M, Islam MR, *et al.* Isolation of Yersinia enterocolitica and Y. intermedia from fatal cases of diarrhoeal illness in Bangladesh. *Transactions of the Royal Society of Tropical Medicine and Hygiene* 1984; **78**: 449–50.

654 Perry RD, Fetherston JD. Yersinia pestis--etiologic agent of plague. *Clin Microbiol Rev* 1997; **10**: 35–66.

655 De Keukeleire S, De Bel A, Jansen Y, Janssens M, Wauters G, Piérard D. Yersinia ruckeri, an unusual microorganism isolated from a human wound infection. *New Microbes and New Infections* 2014; **2**: 134–5.

Table S2. SEM path model latent/composite and measurement variables: sample size (number of countries with data), indicator name, and description (Source: https://data.worldbank.org/indicator unless otherwise stated). Outer models are formative, meaning that the latent variables can be viewed as linear combinations of their respective measurement variable components.

Latent variable	Sample size (# countries, in yr 2015 unless noted)	World bank indicator code (NA if different source) for each measurement variable	Description
Elevation	150 in yr 2010	EN.POP.EL5M.ZS	Population living in areas where elevation is below 5 meters (% of total population)
Elevation	164 in yr1995	NA	High elevation population: % population in Koeppen-Geiger zone h
Political stability and absence of violence	181	PV.EST	Perceptions of the likelihood of political instability and/or politically-motivated violence, including terrorism.
Land area in agriculture	178	AG.LND.CROP.ZS	Permanent cropland (% of land area)
Land area in agriculture	180	AG.LND.AGRI.ZS	Agricultural land (% of land area)
Land area in agriculture	180	AG.LND.ARBL.ZS	Arable land (% of land area)
Biodiversity	180	AG.LND.FRST.ZS	Forest area (% of land area)
Biodiversity	181 in yr 2014	ER.PTD.TOTL.ZS	Terrestrial and marine protected areas (% of total territorial area)
Biodiversity	169 in yr 2012	NA	Species richness of birds*
Biodiversity	169 in yr 2012	NA	Species richness of mammals*
Biodiversity	169 in yr 2012	NA	Species richness of amphibians*
Latitude	181	NA	Absolute latitude in degrees
Health care access	177	SH.XPD.CHEX.PC.CD	Current health expenditure per capita (current US$)
Health care access	181	SH.IMM.MEAS	Immunization, measles (% of children ages 12-23 months)
Health care access	89	WHO Composite coverage index (%)	A weighted score reflecting coverage of eight RMNCH interventions along the continuum of care: demand for family planning satisfied (modern methods); antenatal care coverage (at least four visits); births attended by skilled health personnel; BCG immunization coverage among one-year-olds; measles immunization coverage among one-year-olds; DTP3 immunization coverage among one-year-olds; children aged less than five years with diarrhea receiving oral rehydration therapy and continued feeding; and children aged less than five years with pneumonia symptoms taken to a health facility. Source: http://apps.who.int/gho/data/node.imr.cci2030?lang=en
Rural poor livelihood	180	SH.STA.BASS.ZS	People using at least basic sanitation services (% of population)
Rural poor livelihood	181	SP.DYN.TFRT.IN	Fertility rate, total (births per woman)
Rural poor livelihood	180	SP.RUR.TOTL.ZS	Rural population (% of total population)

Agricultural yield in kcal per person	173 in yr2013	NA	FAO food balance sheet: http://www.fao.org/faostat/en/#data/FBS The total quantity of foodstuffs produced in a country added to the total quantity imported and adjusted to any change in stocks since the beginning of the reference period gives the supply. On the utilization side: separated by quantities exported, fed to livestock, used for seed, put to manufacture for food use and non-food uses, losses during storage and transportation, and food supplies available for human consumption. The per caput supply of each such food item available for human consumption is obtained by dividing the respective quantity by the population actually partaking of it. Data on per caput food supplies are expressed in terms of caloric value and protein and fat content.
Malnutrition	143	SN.ITK.DEFC.ZS	Prevalence of undernourishment (% of population)
Lifespan	181	SP.DYN.LE00.IN	Life expectancy at birth, total (years)
Wealth	58	SI.POV.DDAY	Poverty headcount ratio at $1.90 a day (2011 PPP) (% of population)
Wealth	173	NY.GNP.PCAP.PP.CD	GNI per capita, PPP (current international $)
Tropical climate	164 in yr1995	NA	Tropical population: % 1995 pop in Koeppen-Geiger tropics (Af+Am+Aw); source: https://www.pdx.edu/econ/country-geography-data
Tropical climate	164 in yr1995	NA	Temperate population: % 1995 pop in Koeppen-Geiger temperate zones (Cf+Cs+Df+DW); source: https://www.pdx.edu/econ/country-geography-data
Tropical climate	178 in yr1961-99	NA	Country_precipitationCRU: mean precipitation by country for the period 1961-1999. Values are in millimeters (mm); source: http://data.worldbank.org/developers/climate-data-api
Tropical climate	178 in yr1961-99	NA	Country_temperatureCRU: mean temperatures by country for the period 1961-1999. Values are in degrees Celsius; source: http://data.worldbank.org/developers/climate-data-api
Total land area	181	AG.LND.TOTL.K2	Land area is a country's total area, excluding area under inland water bodies, national claims to continental shelf, and exclusive economic zones. In most cases the definition of inland water bodies includes major rivers and lakes.

* Average species richness across all of a country's 10 x 10 km grid cells derived from (Jenkins, Pimm et al. 2013) as also presented in (Wood, McInturff et al. 2017)

Table S3. Global burden of environmentally mediated and direct-contact transmitted infectious diseases across cause categories. Disability Adjusted Life Years (DALYs) lost globally in 2015 due the category I.A: "Communicable, maternal, perinatal and nutritional conditions: Infectious and parasitic diseases" of the World Health Organization's Global Health Estimates (https://www.who.int/data/gho/data/themes/mortality-and-global-health-estimates)

Transmission category	WHO Global Health Estimates category	WHO category description	Global DALYs (thousands)	%total classifiable DALYs
Environmentally mediated infectious diseases	I.A.1	Tuberculosis (*M. bovis*)	1,569	0.5%
	I.A.4	Diarrheal disease (subset of environmentally-transmitted causes tracked: e.g. *Shigella* spp., *Salmonella* spp., *Vibrio cholera,* Norwalk virus)	46,456	13.9%
	I.A.5.d	Childhood cluster diseases: Tetanus	4,681	1.4%
	I.A.6	Meningitis (subset of environmentally-transmitted causes tracked: e.g. *Naegleria fowleri*, *Cryptococcus neoformans*, *Histoplasma capsulatum*)	7,562	2.3%
	I.A.7	Encephalitis (subset of environmentally-transmitted viruses tracked: e.g. Japanese encephalitis virus, Louping ill virus)	6,050	1.8%
	I.A.8.a	Acute Hepatitis A	1,044	0.3%
	I.A.8.d	Acute Hepatitis E	2,525	0.8%
	I.A.10.a	Parasitic and vectorborne disease: Malaria	38,520	11.5%
	I.A.10.b	Parasitic and vectorborne disease: Trypanosomiasis	372	0.1%
	I.A.10.c	Parasitic and vectorborne disease: Chagas	253	0.1%
	I.A.10.d	Parasitic and vectorborne disease: Schistosomiasis	3,541	1.1%
	I.A.10.e	Parasitic and vectorborne disease: Leishmaniasis	1,357	0.4%
	I.A.10.f	Parasitic and vectorborne disease: Lymphatic filariasis	2,071	0.6%
	I.A.10.g	Parasitic and vectorborne disease: Onchocerciasis	1,136	0.3%
	I.A.10.h	Parasitic and vectorborne disease: Cysticercosis	1,857	0.6%
	I.A.10.i	Parasitic and vectorborne disease: Echinococcosis	642	0.2%
	I.A.10.j	Parasitic and vectorborne disease: Dengue	2,613	0.8%
	I.A.10.k	Parasitic and vectorborne disease: Trachoma	279	0.1%
	I.A.10.l	Parasitic and vectorborne disease: Yellow fever	856	0.3%
	I.A.10.m	Parasitic and vectorborne disease: Rabies	1,672	0.5%
	I.A.11.a-c	Intestinal nematodes	3,395	1.0%
	I.A.11.d	Foodborne trematodes	1,066	0.3%
		**Subtotal: DALYs caused by environmentally transmitted infections**	**129,488**	**38.7%**
Non-environmentally mediated infectious diseases	I.A.1	Tuberculosis (*M. mycobacterium*)	54,468	16.3%
	I.A.2.a	STDs excluding HIV: Syphilis	8,065	2.4%
	I.A.2.b	STDs excluding HIV: Chlamydia	955	0.3%
	I.A.2.c	STDs excluding HIV: Gonorrhoea	1,187	0.4%
	I.A.2.d	STDs excluding HIV: Trichomoniasis	173	0.1%
	I.A.2.e	STDs excluding HIV: Genital Herpes		0.1%
	I.A.2.f	STDs excluding HIV: Other STDs	734	0.2%
	I.A.3	HIV/AIDS	91,907	27.4%
	I.A.4	Diarrheal disease (subset of contagious causes tracked: e.g. Rotavirus C/F, Human adenovirus A-F)	45,177	13.5%
	I.A.5.a	Childhood cluster diseases: Whooping cough	6,142	1.8%
	I.A.5.b	Childhood cluster diseases: Diptheria	223	0.1%
	I.A.5.c	Childhood cluster diseases: Measles	11,531	3.4%
	I.A.6	Meningitis (subset of contagious causes tracked: e.g. meningococcal meningitis, pneumococcal meningitis, *Haemophilus influenzae*)	20,373	6.1%
	I.A.8.b	Acute hepatitis B	6,416	1.9%
	I.A.8.c	Acute hepatitis C	1,270	0.4%
	I.A.10.h	Parasitic and vectorborne disease: Leprosy	257	0.1%
	I.A.10.j	Parasitic and vectorborne disease: Trachoma	299	0.1%
		**Subtotal: DALYs caused by non-environmentally transmitted infections**	**205,353**	**61.3%**
Unclassifiable DALYs	I.A.12	**Other infectious diseases (not clearly classifiable by DALYs):** mixed causes	**24,500**	--
Total DALYs		**Total DALYs**	**359,341**	**100%**

Table S4. Full and reduced PLS-SEM model structure (in matrix form): a "1" in the cell indicates a direct path (connecting "from" row "to" column) was hypothesized and therefore included in the full model. Asterisks indicate the subset of variables included in the reduced model after bootstrapping whereby we identified and excluded those paths with low support, i.e. those with p>0.1 in the full model. An underline underneath the 1 indicates that a path was included in the reduced model, but not shown in Figure 4, for simplicity, because it was a "dead-end" path, in that there was neither a direct nor indirect significant path that led to environmentally mediated or directly transmitted disease burdens (the outcomes of interest). See figure S2 for the reduced (final) model depicting all the asterisked paths.

	Land area in agriculture	Biodiversity	Directly transmitted	Environmentally mediated	Elevation	Agricultural yield	Health care access	Latitude	Life expectancy	Malnutrition	More tropical climate	Political stability	Rural livelihood	Total land area	Wealth
Land area in agriculture		1	1	1		1				1			1		
Biodiversity				<u>1</u>*											
Directly transmitted									<u>1</u>*						
Environmentally mediated									<u>1</u>*						
Elevation	1	1	1	1		1					1				1
Agricultural yield		1	1	1					1	1			1		
Health care access			1	<u>1</u>					<u>1</u>*	1			1*		
Latitude	1	1	1	1		<u>1</u>*			1		1*				1*
Life expectancy															
Malnutrition			<u>1</u>*	1					1						
More tropical climate	1	<u>1</u>*	1	1		<u>1</u>*							1*		1
Political stability			1	1			1*			1					1*
Rural livelihood			1*	1*					<u>1</u>*	<u>1</u>*					
Total land area	1	1	1	1		<u>1</u>*			1				1		1
Wealth	1	1	1	1*		1	1*		<u>1</u>*	1			1*		

Table S5. Reduced (final) model fits, R^2 for each variable fit

	R Square	R Square Adjusted
Ag yield in kCal per person	0.146	0.132
Biodiversity	0.244	0.239
**Direct-contact infectious burden**	**0.411**	**0.405**
**Environmentally mediated infectious burden**	**0.631**	**0.624**
Health care access	0.546	0.541
Life expectancy	0.905	0.902
Malnutrition (undernutrition)	0.349	0.346
More tropical climate	0.758	0.757
Rural livelihood – rurality, poor sanitation, high fertility	0.630	0.624
Wealth	0.359	0.352

Figure S1. Latitudinal range limits of direct-contact transmitted and environmentally mediated infectious diseases. Graphs show total number of diseases reported from each country (ISO codes), organized by increasing absolute latitude of the country centroids along the x axis. A) top panel shows total number of directly transmitted versus environmentally mediated diseases with >0 burden in each country (moving average spline), and B) bottom two panels show identities of directly transmitted (middle panel) and environmentally mediated (bottom panel) infectious diseases with >0 burden in each country, displayed as stacked bars. Diseases are represented in the stacked bar plots in alphabetical order from bottom to top, with the key below. Environmentally mediated infectious disease ranges appear more limited by increasing latitude (more of them are absent from countries at higher latitudes) than direct-contact transmitted diseases.

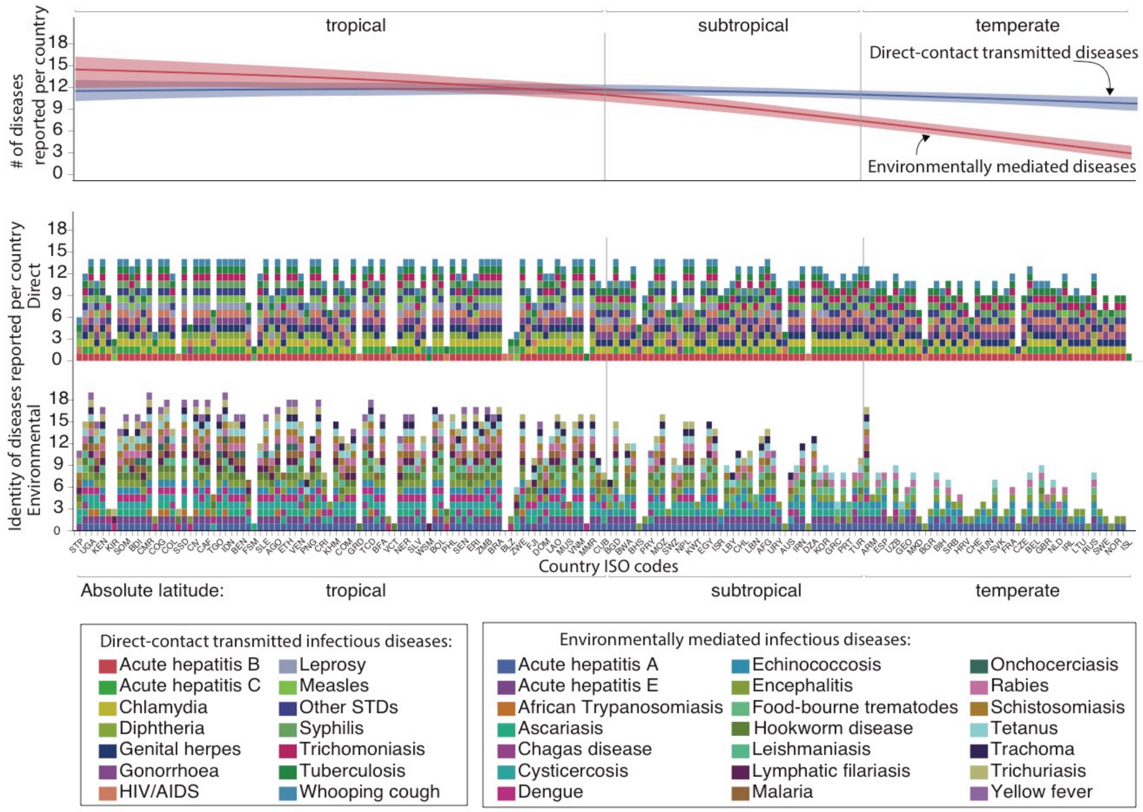

Figure S2. PLS-SEM results: reduced model all paths. Statistically significant paths links are shown in red for negative associations, black for positive associations, and grey for marginally significant (p >0.05 but <0.1) paths among the variables linked by those lines. Numbers along paths (and also path thickness) correspond to the weighted correlation coefficients which signify the strength of the association between two linked variables; total effects can be estimated by multiplying path coefficients along one or more segments, and summing across all possible paths. Total significant effects on disease burdens are summarized in Table 2; Paths with p>0.1 were removed from the full model to produce the final reduced model shown here (see Table 3 in the main text). A simplified version of this figure is shown in the main text Figure 4 (with only those paths that link to the main outcomes of interest, directly transmitted or environmentally mediated infectious diseases, with p<0.05). Artwork credit: N. Nova.

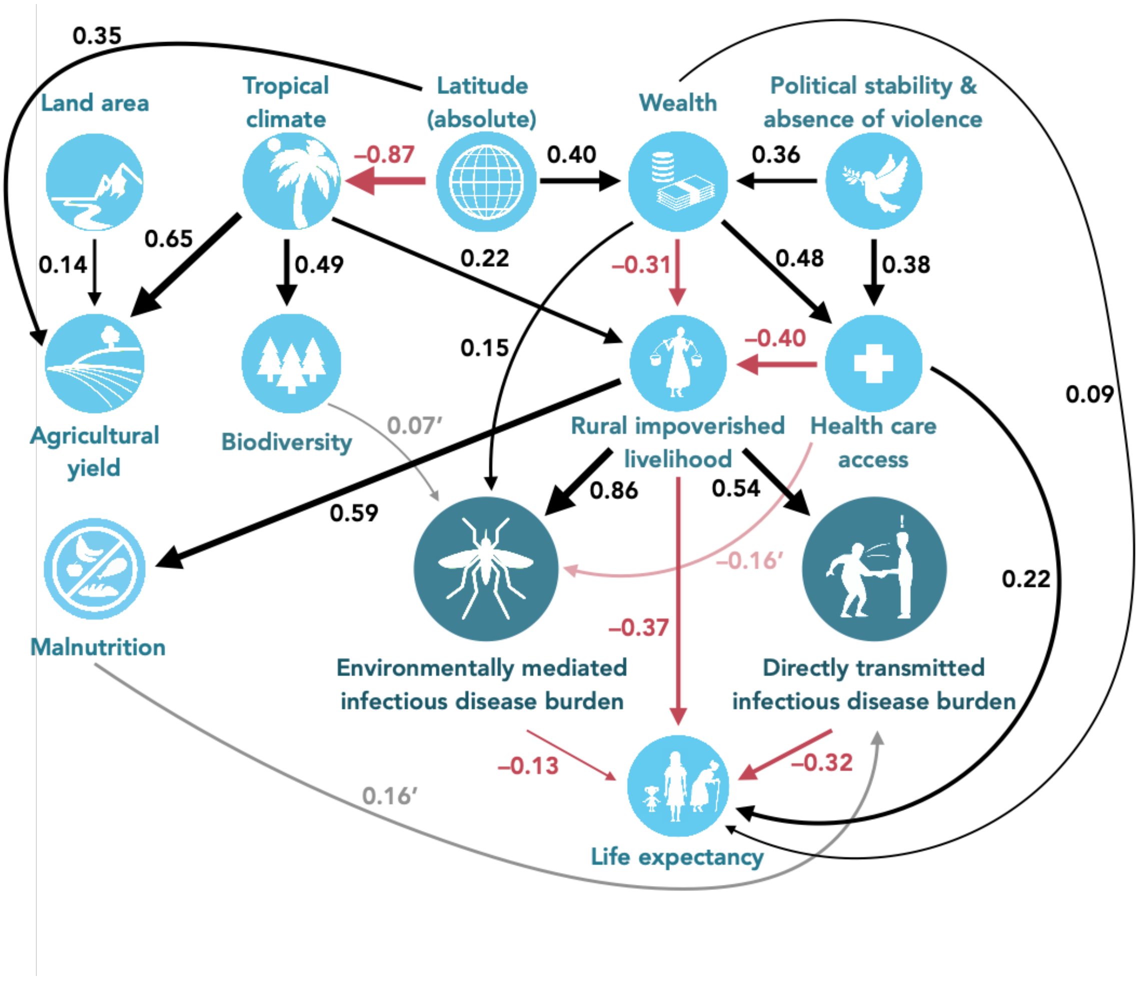

Supplement: Supplementary appendix [file mmc1.pdf]
